# Predicted mouse interactome and network-based interpretation of differentially expressed genes

**DOI:** 10.1371/journal.pone.0264174

**Published:** 2022-04-07

**Authors:** Hai-Bo Zhang, Xiao-Bao Ding, Jie Jin, Wen-Ping Guo, Qiao-Lei Yang, Peng-Cheng Chen, Heng Yao, Li Ruan, Yu-Tian Tao, Xin Chen

**Affiliations:** 1 Institute of Big Data and Artificial Intelligence in Medicine, School of Electronics & Information Engineering, Taizhou University, Taizhou, China; 2 Institute of Pharmaceutical Biotechnology, School of Medicine, Zhejiang University, Hangzhou, China; 3 Joint Institute for Genetics and Genome Medicine between Zhejiang University and University of Toronto, Zhejiang University, Hangzhou, China; Luxembourg Institute of Health, LUXEMBOURG

## Abstract

The house mouse or *Mus musculus* has become a premier mammalian model for genetic research due to its genetic and physiological similarities to humans. It brought mechanistic insights into numerous human diseases and has been routinely used to assess drug efficiency and toxicity, as well as to predict patient responses. To facilitate molecular mechanism studies in mouse, we present the Mouse Interactome Database (MID, Version 1), which includes 155,887 putative functional associations between mouse protein-coding genes inferred from functional association evidence integrated from 9 public databases. These putative functional associations are expected to cover 19.32% of all mouse protein interactions, and 26.02% of these function associations may represent protein interactions. On top of MID, we developed a gene set linkage analysis (GSLA) web tool to annotate potential functional impacts from observed differentially expressed genes. Two case studies show that the MID/GSLA system provided precise and informative annotations that other widely used gene set annotation tools, such as PANTHER and DAVID, did not. Both MID and GSLA are accessible through the website http://mouse.biomedtzc.cn.

## Introduction

Because of its close genetic and physiological similarity to human, the ease of the manipulation and analysis of its genome, the convenience of its breeding in the laboratory, the house mouse, *Mus musculus*, has emerged as a leading model of human biology and disease [1, 2]. Genomic studies have highlighted that the genome of mice is very similar to that of human. 99% of mouse protein coding genes have human orthologues [3–5]. These similarities to human, together with the development of powerful methods and tools for mouse research, have greatly expanded our understanding of human biology [1, 6]. However, because of technological limitations, a limited number of experimentally reported protein-protein interactions have been integrated into mouse databases. Therefore, an accurate prediction interactome with high coverage is valuable for mouse researchers.

In addition to databases integrated with interactions from experiments, studies of potential interaction prediction based on high-throughput technology have also been a focus area, including MouseNet [7], mentha [8], MIST [9], Hitpredict [10], and STRING [11]. However, the computational identification of potential interactions shows a high false-positive rate. A prediction approach was hence developed with indirect protein interactions, such as gene coexpression and gene colocalization [12]. These studies reported that the predicted protein interactions were more accurate than the high-throughput experimental data. Further studies demonstrated that it is possible to directly infer protein interactions from this indirect evidence alone [13]. To ensure the accuracy and effectiveness of protein interaction prediction using indirect evidence, various types of evidence have been thoroughly assessed [14, 15]. These investigations broaden our horizons on how to accurately predict protein interactions on a proteomic scale.

The process of attaching biological information to a set of simultaneously changed genes (genes that are differentially expressed, GDE) is knowns as functional annotation, which is a frequent component of bioinformatics analysis in omics research [16]. As the state of the art, functional annotation of GDE observed in an omics research relies on enrichment analysis [17]. Currently, a series of enrichment-based tools are widely used for the analysis of observed GDEs, including PANTHER [18], KEGG [19], DAVID [20], etc.

The enrichment-based strategy summarizes the observed GDEs to established biological concepts. This strategy is successful in many cases. However, when there is no established annotation term that can accurately describe these changes, enrichment-based approaches frequently report terms that are conceptually very general (such as GO: 0016020, membrane) or simply report no term. These results provided limited help for investigators to formulate further hypotheses and design studies to elucidate the mechanism underlying the observed GDE. On the other hand, even in cases that no established biological concept is available to accurately describe what these GDEs are, we may still use established biological concepts to describe what potential functional impacts may be collaboratively exerted by these GDEs. For instance, the observed GDEs may collectively interfere with the function of GO:1903393 (positive regulation of adherens junction organization), even when the GDEs themselves are not enriched with this term (an example is provided in Discussion).

To interpret the potential functional impacts of observed GDEs, we developed a web tool, gene set linkage analysis (GSLA), which complements the existing enrichment-based approaches, and are available for human and Arabidopsis transcriptome interpretations [21, 22]. The strategy of GSLA interpretation is that if a GDEs is frequently functionally associated with genes in a biological process, then the GDE is expected to interfere with this biological function. Successful interpretations by GSLA require a high-quality functional association network, such as the human interactome resource (HIR) and predicted Arabidopsis interactome resource (PAIR) that we developed for human and Arabidopsis GSLA [21, 23].

In this work, we developed a high-quality functional gene association network, the mouse interactome database (MID), for searching potential functional gene associations in mice. We also constructed the GSLA web tool for interpretation of mice transcriptomes. To infer high-quality functional associations between mice protein-coding genes, MID integrates six types of evidence from 9 public databases. All evidence used for inference predate the end of 2018. Newly reported experimentally confirmed protein-protein interactions (after 2018) were used to assess the inference accuracy. The current version of MID includes 155,887 gene associations. These gene associations are expected to cover 19.32% of the protein-protein interactions in mice, and 26.02% of the gene associations may represent protein interactions. The web interface for MID is available for users to investigate the functional associations among the protein-coding genes, and provides a GSLA web tool for interpretation of the collective functional impacts of mice GDEs. In the end, two case studies are provided to illustrate the use of the MID/GSLA system.

## Materials and methods

### Evidence data of functional gene interactions

**Fig 1:**
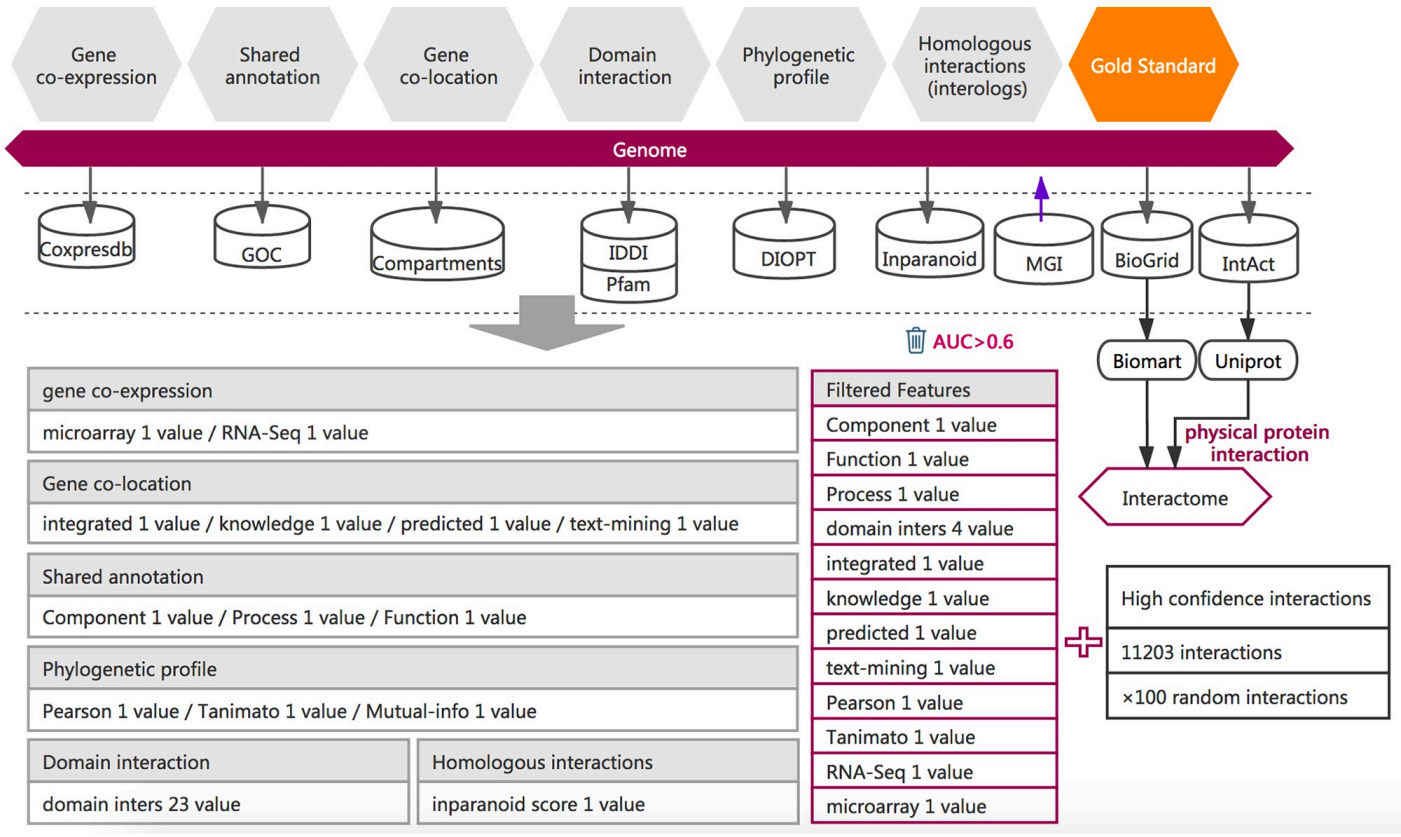
The workflow for the prediction of functional associations between mouse genes. The training dataset consists of 1). Positive examples. High-quality protein interactions that were reported in at least two databases were used as positive examples. 2). Negative examples. Random gene pairs that did not overlap with the positive examples (positive: negative = 1:100). 3) Functional association evidence. Six types of functional association evidence data were collected from 9 databases. 36 different mathematical characterizations of these six types of evidence data produce 36 feature values, which were described in S2 Table. A total of 15 high-quality features (AUC > 0.6) were selected as high-quality features and were used for functional association inference. Details of the feature computing methods can be found in the MID website (the section of Indirect Evidence).

Protein-protein interactions are considered evidence of strong functional associations. A total of 32,997 experimentally reported unique protein-protein interactions between mouse protein-coding genes were collected from BioGRID [24], and IntAct [25] (S1 Table). To ensure the quality of our collected protein-protein interactions reported by experiments, we removed interactions that were reported in less than two independent studies and those that were reported only in high-throughput experiments. After filtration, 11,203 protein-protein interactions with high quality were left for subsequent support vector machine model training so that we could obtain the predicted functional associations that are as strong as protein interactions. In this study, we used UniProt [26] and BioMart software [27] to convert different gene IDs to the unique MGI ID based on the reference gene ID from the MGI database [28] (Fig 1).

Apart from the experimentally confirmed protein-protein interactions, we also collected six types of functional association evidence from 7 public databases with the year set to before 2018. These evidence data include 17,738 expression profiles (COXPRESdb) [29], 402,516 gene annotations (GOC) [30], 104,093 domain interactions (IDDI and Pfam) [31, 32], 22,515 subcellular gene localizations (Compartments) [33], 22,380 phylogenetic profiles (DIOPT) [34], and inparalog/ortholog relationships between 15,115 mouse proteins and proteins from *A*. *thaliana*, *C*. *elegans*, *H*. *sapiens*, *D*. *melanogaster*, *R*. *Norvegicus*, *S*. *cerevisiae* and *S*. *pombe* to compute interologs [35]. 36 features belonging to six categories were computed based on these evidence data, each suggesting a certain kind of functional association (Fig 1 and S2 Table) [36, 37]. Detailed methods and equations can be found in website help (the indirect evidence section, http://mouse.biomedtzc.cn./#/help/feature).

### Computation of feature values

To characterize the functional associations between mouse protein-coding genes, 36 feature values were selected for computation (Fig 1 and S2 Table). The 36 feature values include 1 homologous interaction feature, 3 phylogenetic profile features, 23 domain interaction features, 4 subcellular co-localization features, 2 coexpression features and 3 shared annotation features (S3 Table).

### The calculation of functional gene interactome size

To calculate the fraction of protein interactions that were covered by these putative functional gene interactions, we used the following equation.

$${N_{interactome}\times Sensitivity+{(N}_{all-pairs}-N_{interactome})\times (1-specificity)=N}_{predict}$$

Here, *N*_*interactome*_ is the estimated number of mouse protein interactions; *N*_*predict*_ is the size of the predicted functional gene interactome; *N*_*all-pairs*_ is the number of all protein-coding gene pairs in mice; and the sensitivity and specificity measure the accuracies of the prediction model to predict the newly published (after 2018) protein interactions and random gene pairs.

### Evaluation of feature values

To evaluate the power of our selected 36 feature values to indicate functional associations, we used the area under the curve (AUC) of the receiver operating characteristic (ROC) curve. For the computation of protein-protein interaction predictions, each feature value will produce a series of sensitivities and specificities based on different cut-offs with the training dataset (collected before 2018). The sensitivity and specificity pairs of the ROC curve (X-axis, 1-specificity; Y-axis, sensitivity) were plotted corresponding to different cut-offs. In this study, the feature values with an AUC greater than 0.6 were considered informative to indicate functional associations (S1 Fig). A total of 15 features were finally selected for functional association prediction.

### Functional association inference between mouse protein-coding genes

To train and infer functional gene associations, we used the LIBSVM software package [38] with the above selected 11,203 high-confidence, experimentally-confirmed protein-protein interactions, which served as positive examples during the prediction model training. The collection date of these high-confidence protein interactions was reported before 2018. During the prediction model training, negative examples are also needed. In this study, the negative model involved the gene pairs that were randomly generated after removing the overlapping gene pairs with the positive examples. These randomly generated gene pairs may include rare false negatives. To reduce the impact from the low probability of randomly generated gene pairs with strong functional associations, the positive-to-negative ratio was set as 1:100 in the training dataset to assume that only a small fraction of random gene pairs could have strong functional associations as in the real-word scenario.

To train the SVM prediction model, we used the soft-margin Gaussian kernel algorithm. A 5-fold cross-validation method was implemented to evaluate the sensitivity and specificity, the optimal harmonic mean of which was targeted by the kernel width parameter σ and soft margin parameter C. The optimized σ and C were used to train the prediction model, which was then validated by the experimentally reported protein-protein interactions published after 2018 and the randomly generated negative examples. Finally, our optimized model reported 155,887 functional associations with a sensitivity of 19.32% and a specificity of 99.95%. Table 1 shows how well different predicted interactomes included the newly published protein interactions. For this assessment, only those predicted interactomes were included (i.e., STRING, the predicted interactions in MID, MouseNet [7], and MIST [9]). Datasets comprising of only experimentally reported interactions were not included, as they are sources of our newly published gold-standard protein interactions. In this comparison, only MID showed a balance between sensitivity and reliability.

**Table 1:** Evaluation of the predicted interactions in available mice interactomes.

Interactome	Sensitivity	Reliability
STRING	52.17%	1.06%
MIST	28.99%	5.17%
MID	19.32%	26.02%
MouseNet	21.26%	5.23%

Applying this model to all mouse protein-coding gene pairs produced 144,477 inferred functional associations. These inferred functional interactions together with the 11,410 known protein interactions make the MID dataset, which consists of 155,887 interactions. Solving this equation that described in the methods section, we obtained the estimated mouse protein interactome size of 1.95 x 10^5^. Based on the estimated interactome size (1.95 x 10^5^) and the estimated sensitivity (19.32%, the lower one of training stage sensitivity 19.40% and evaluation stage sensitivity 19.32%), the predicted interactions in MID is expected to include 144,477 rue protein interactions. Therefore, 26.02% of the MID functional interactions (37,592 out of 144,477) are expected to represent protein interactions.

### Website construction

The LNMP system is an integrated system that was used to deploy the online database. The LNMP system includes Linux, Nginx, MySQL, and PHP. We used the MySQL database to store data. The web interface of the online database was developed using the Laravel framework using PHP. The front-end of the online database was implemented with the Vue.js script library, which implements single page application (SPA). Vue.js is an open source JavaScript library designed for SPA web interface creation. Cytoscape [39] was used for the visualization of the functional association networks.

### Microarray and RNA-seq data analysis

From the GEO database [40, 41], we retrieved the microarray dataset GSE39989 and RNA-seq dataset GSE135282. The microarray dataset GSE39989 compared gene expression between wild type and *Olfm4*-knockout mice in the prostate tissue [42]. Five biological replications were used for the *Olfm4* (+/+) or *Olfm4*-knockout (-/-) prostate RNA extracted from five individual mice. The RNA-seq dataset GSE135282 showed 560 genes were up-regulated and 297 genes were down-regulated in *Piezo1*-knockout (*Piezo1*^*fl/fl*^) mice relative to the wild type. Four biological repeats were performed for both the wild type and *Piezo1*-knockout mice.

In this study, we used the online tool GEO2R [43–45] to re-analyse these two dataset with default parameters. The top 250 transcriptionally changed genes were selected for annotation. The microarray dataset was selected based on the P value (P Value < 0.05) and the RNA-seq dataset was based on the FDR value.

## Results

### Evaluation of the predicted functional gene association network

To evaluate the quality of the predicted functional gene association network of MID, we measured its capacity to group functionally related genes together. This capacity is evaluated as the accuracy of using a gene’s network neighbours to predict the gene’s function, i.e., the “guilt-by-association” prediction of gene functions. We evaluated the newly inferred mouse interactome (MID) together with five other available interactomes, including MouseNet [7], mentha [8], MIST [9], Hitpredict [10], and STRING [11]. For each gene in each interactome, its GO biological process annotations were predicted as the terms enriched in the annotations of its first-degree network neighbours. Here, the term enrichment tool PANTHER [18] was used to find enriched annotation terms.

The data used to predict functional gene interactions in MID were collected before 2018 (Dec 31,2017). A total of 7,935 genes with new annotations (added after Dec 31, 2017) were collected from the GO database [46, 47] to evaluate the prediction accuracy. These genes had a total of 327,092 annotations, of which 40,949 annotations were newly added. We relied on these genes and their annotations to evaluate the gene function prediction performance.

We used the precision-recall curve to measure the overall accuracy of new annotation prediction across six interactomes. Here, precision means the proportion of annotations predicted by PANTHER that were consistent with the total 327,092 annotations. Recall means the proportion of PANTHER-reported annotations that were successfully covered 40,949 newly added annotations. Each PANTHER-predicted annotation has an enrichment significance (P-value). Therefore, when different cut-offs on P-values were applied, the number of annotations predicted by PANTHER will change accordingly. More reported annotations would result from a higher cut-off, which would lead to higher recall but lower precision. In contrast, if a lower cut-off was used, fewer annotations would be predicted, leading to more reliable predictions and higher precision. In general, the precision-recall curve is a cut-off-independent approach that shows the advantage of providing a more comprehensive view of the capability of an interactome to predict new gene annotations. An interactome with a higher AUC may be better to support “guilt-by-association” prediction of gene function.

**Fig 2:**
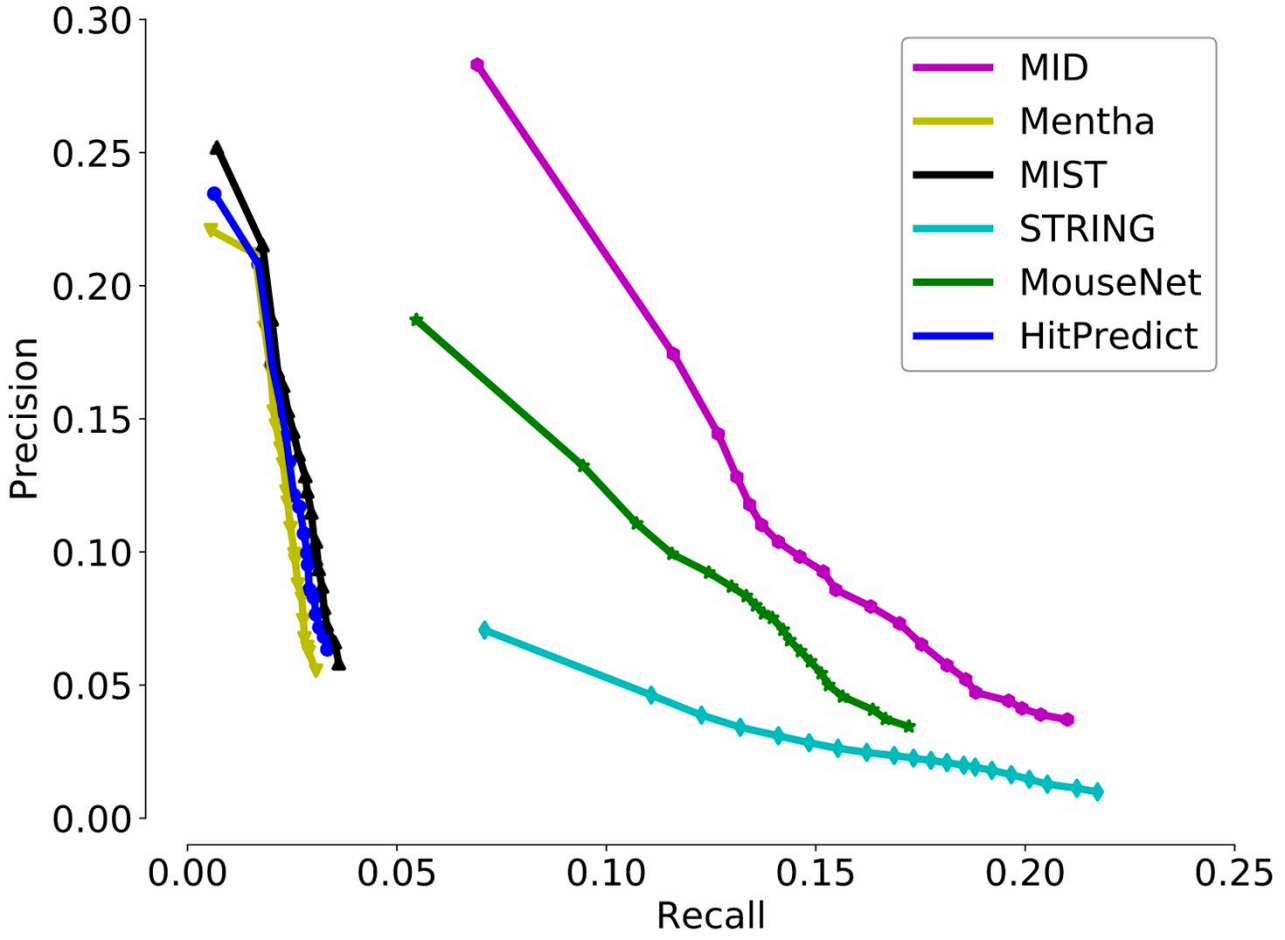
Quality assessment of six interactomes. To assess the quality of our predicted functional association network, MID, we evaluated how well it groups functionally related genes together. Precision measures the fraction of correct annotations predicted using an interactome, while recall measures the fraction of new annotations successfully predicted using an interactome.

The precision-recall curves of the six interactomes are shown in Fig 2. MID shows the best performance in the prediction of new annotations with the evidence of its curves located above others. When MID reached the high-recall region, it still maintains the highest precision. Although the curves of the STRING and MouseNet interactomes reached the high-recall region, their precisions were low; especially for STRING, its precision did not increase as much as that of other interactomes in the low-recall region. This observation indicates that the STRING interactome may contain a high proportion of weak functional gene associations. However, except for STRING and MouseNet, the other interactomes did not reach the high-recall region. In conclusion, only MID shows balanced coverage and precision during gene function prediction when compared to the other five interactomes.

### The website interface of MID/GSLA

**Fig 3:**
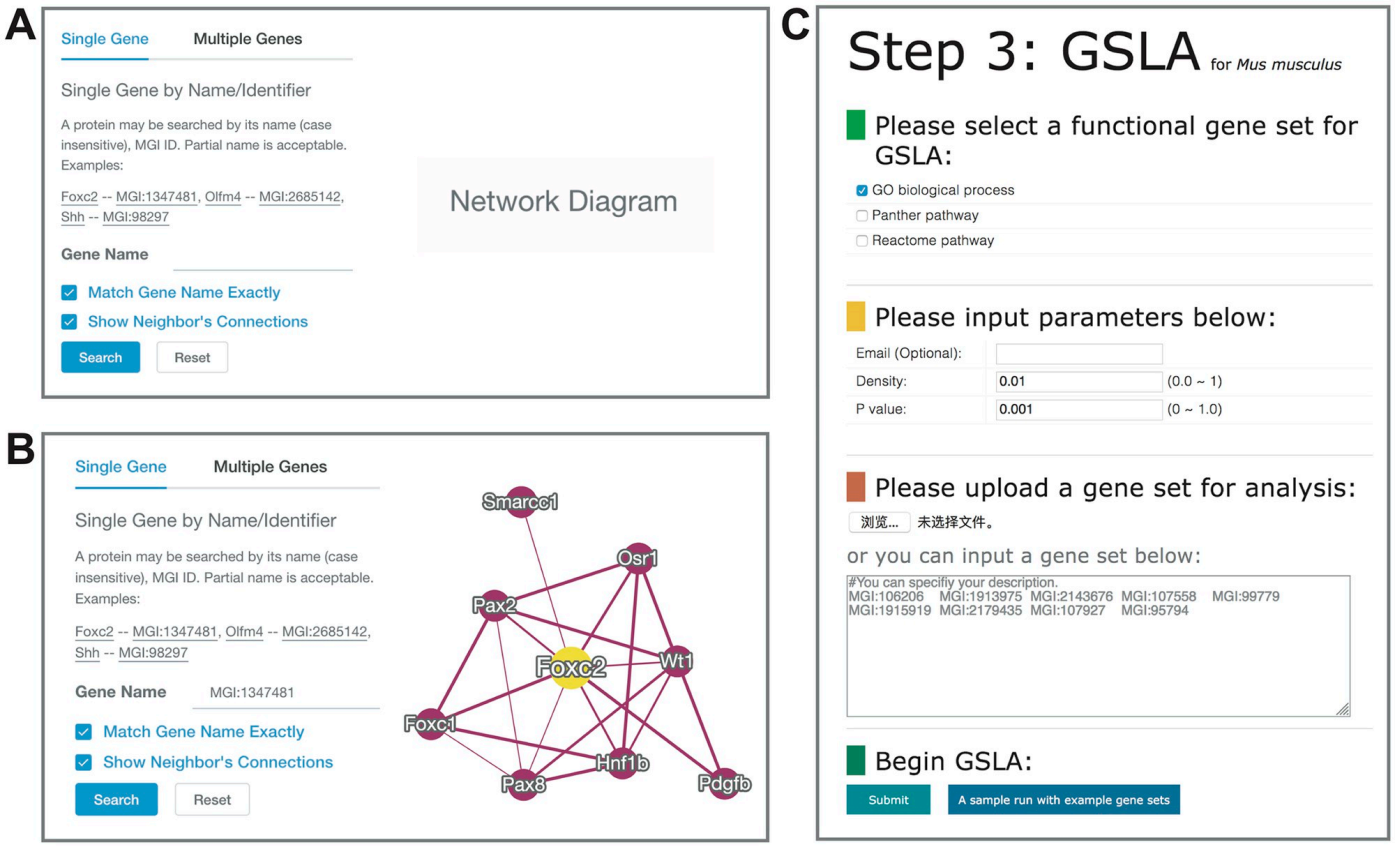
MID website. (A) Single gene search and multiple gene search interface. (B) Search result page. The functional associations between the query genes are illustrated in a graphical view. Right clicking on an interaction in the diagram will show its details. (C) The gene set linkage analysis webtool, GSLA interface. GSLA prefers to use MGI ID because the internal sever works only with MGI ID, it will map other ID systems (MGI ID, gene name, UniProt ID, Ensembl gene ID, Ensembl protein ID, and NCBI Entrez ID) to MGI IDs.

We provide two search modes in the MID website: single gene search and multiple gene search (Fig 3A). The single search option reports all inferred functional gene interactions containing the query gene, while the multiple search option reports the whole functional gene interactions between two query genes. In the MID website, both the gene name and MGI ID are offered for users to query their genes of interest. The resulting putative functional interactions are listed in tabular form (Fig 3B). A graphical view of these functional interaction networks is presented on the right side of the query interface. Moreover, in the network diagram, users can right-click on their interested interaction, which will show the feature values used in our prediction model for this interaction. If users click on a node of the interested gene, the detailed annotations of this selected gene will be shown. Users can download all putative functional gene interactions. The functional gene interaction network also provides a download link for users.

**Fig 4:**
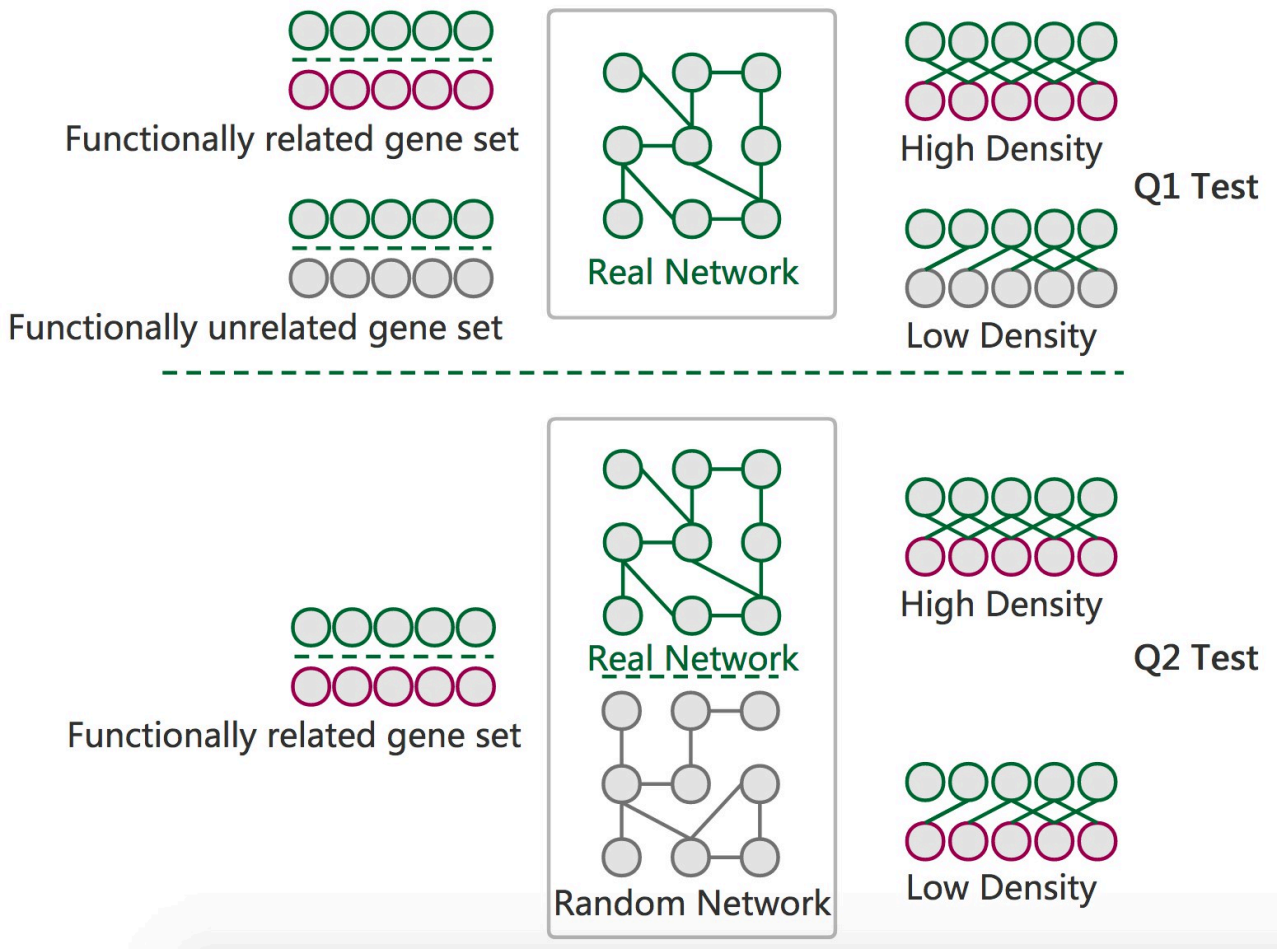
The GSLA interpretation strategy. GSLA uses two hypothesis tests to identify biologically significant functional associations between two gene sets. Q1 evaluates whether the inter-gene-set interaction density between two gene sets is higher than that between random gene pairs. Q2 evaluates whether the dense functional interactions between gene sets can only be observed within the biologically correct network, rather than in randomly generated interactomes with the same node degree distribution.

Previously, we developed the GSLA tool as a transcriptomic analysis tool for potential functional impact predictions of Arabidopsis based on the observed GDEs [22]. The strategy of GSLA evaluates whether a set of changed genes have more frequent functional interactions with genes that comprise a biological process or biological function. Here, we used two hypotheses (Q1 and Q2) to measure the significance of the functional associations between two gene sets (Fig 4). Q1 measures whether the inter-gene set gene association density between functionally associated gene sets is higher than the background gene association density between random gene sets. Q2 measures whether the functionally associated gene sets with high density can be only observed in the biologically correct functional gene interaction network (our knowledge of molecular mechanisms). In other words, Q2 assumes that the density in MID is higher than that in a random functional gene interaction network consisting of the same genes, with each gene having the same number of neighbours. Therefore, from a biological perspective, Q1 evaluates the strength of a functional association between two gene sets, while Q2 verifies that the observed strong functional association is the result of a biologically correct network topology that represents our knowledge of the molecular mechanisms rather than the result of the compositions of these two gene sets. In fact, some genes, such as hubs, may have substantially more neighbours in the interactome than others. Gene sets may easily have much more inter-gene set functional interactions that contain a number of hubs relative to other gene sets without hubs. Q2 is therefore used to control this confounding factor of gene set composition. In general, the two hypotheses, Q1 and Q2, are different but also complementary. They work together to make the functional impact prediction of GSLA more sensitive and more specific.

The default significance cut-offs for GSLA to report a gene set interaction are density>0.01 (Q1) and p<0.001 (Q2).

The GSLA web service is provided on the MID website and is based on GSLA, which is used to interpret the potential functional impacts of the observed GDEs in the mouse transcriptomic experiments. The main website interface of GSLA is presented in Fig 3C. When users submit a set of GDEs, GSLA can recognize six types of mouse gene IDs, including MGI ID, gene name, UniProt ID, Ensembl gene ID, Ensembl protein ID, and NCBI Entrez ID. GSLA prefers to use MGI ID because the internal sever works only with MGI ID. While MGI IDs provide a framework for unification of various gene IDs, most experiments are performed with the Ensembl and RefSeq gene IDs. Therefore, the GSLA web service provides a functionality that automatically converts other ID systems (MGI ID, gene name, UniProt ID, Ensembl gene ID, Ensembl protein ID, and NCBI Entrez ID) to MGI IDs, so that the analysis can be performed on the predicted mouse functional gene interactome based on MGI IDs. To avoid a user’s query loss, it is suggested that users provide GDEs directly as MGI IDs. The cut-offs for Q1 and Q2 (density and p) of the GSLA web tool can be adjusted by users (Fig 3C). Before submission, an email address is needed for receiving the analysis results, the top ten lines of which are the analysis parameters. S4 Table shows the identified functionally associated biological processes, functional gene interactions between the GDEs and genes in the query GDEs. Finally, the top 50–200 GDEs as a query dataset is suggested for users to obtain specific and focused functional impact annotations.

### Using the MID/GSLA system to re-analyse the *Olfm4*-knockout mice microarray dataset

Prostate cancer is common in males and is the second leading cause of cancer-related death in men in the United States [48]. The roles and molecular mechanisms in human prostate cancer progression are not completely understood. The olfactomedin 4 (OLFM4) gene in humans has been documented to express normally in prostate tissue but reduced in prostate cancer cells [49]. To explore the effects of OLFM4 on the progression of human prostate cancer, Li et al. utilized Olfm4-knockout mice to investigate the function of *Olfm4* in murine tissues [42]. They discovered that the Hedgehog signalling pathway was significantly upregulated with *Olfm4*-knockout, and the loss of *Olfm4* promoted progression of prostatic neoplasms. Li et al. also found that OLFM4 protein interacts with sonic hedgehog protein [42]. These discoveries were consistent with previous results that Hedgehog signalling mediates prostate ductal morphogenesis and prostate cancer cell metastasis [42, 50–52]. Together, these data suggest that olfactomedin 4 plays an important role in the regulation of prostate cancer progression.

**Fig 5:**
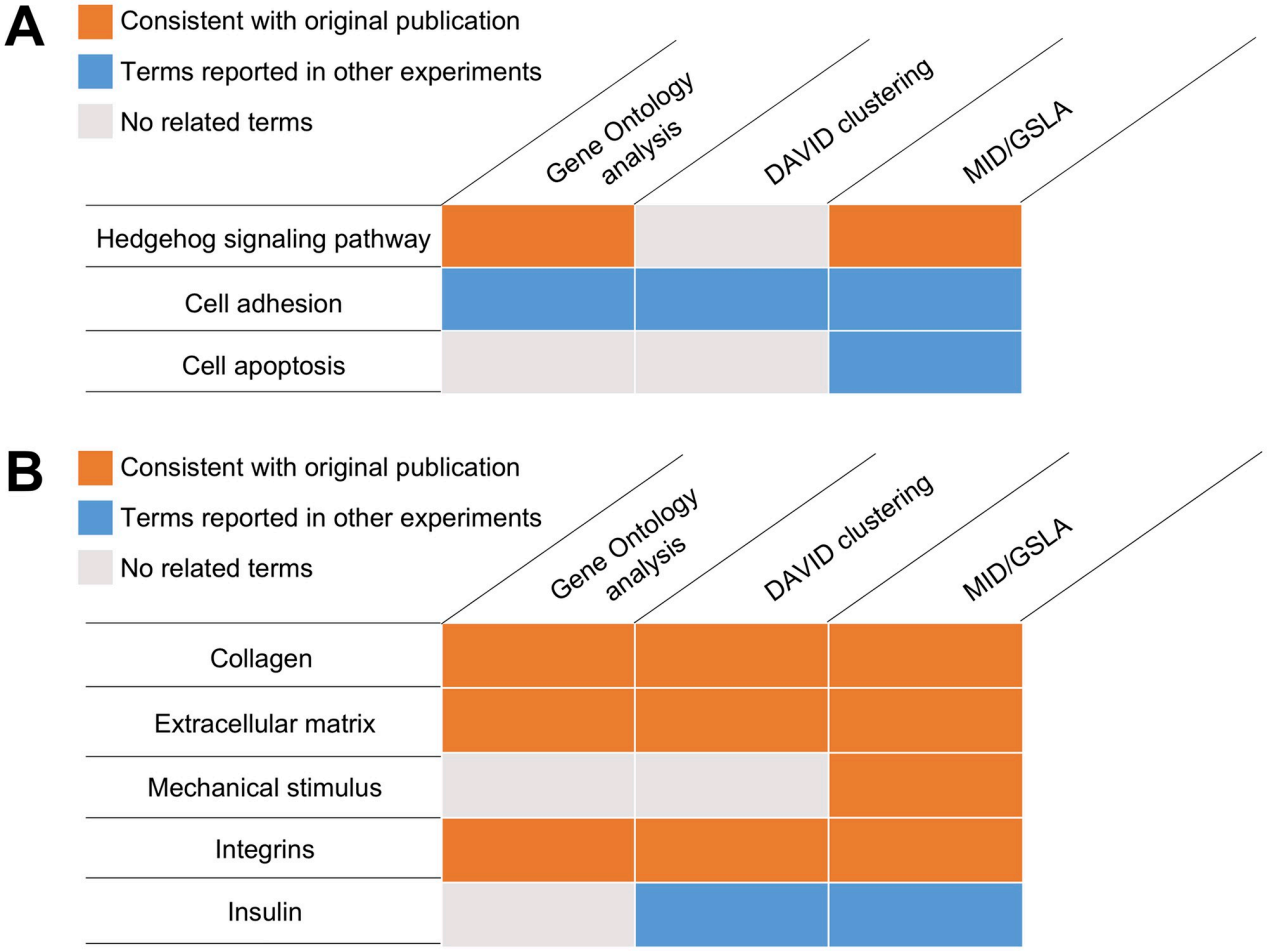
The functional categories of the terms reported by MID/GSLA, DAVID, and PANTHER (GO ontology analysis). The annotations produced by MID/GSLA are more comprehensive and informative for further mechanistic study.

Three gene set annotation tools, MID/GSLA, DAVID [20], and GO enrichment analysis [46, 47] were compared for their usefulness to derive functional insights from genes that changed expression in *Olfm4*-knockout mice (GEO database: GSE39989) [42]. DAVID, a widely used tool that relies on a term clustering technology, reported a total of 261 terms in 42 clusters (S5 Table). Among these terms, Hedgehog signalling and related pathways were not found (Fig 5A). Both DAVID and GO enrichment analysis identified cell adhesion related pathways (Fig 5A), though these pathways were not the major functional impacts subsequent *Olfm4*-knockout, as stated in the original publication [42]. In contrast, MID/GSLA reported 13 terms (S4 Table). In addition to the Hedgehog signalling and cell adhesion-related pathways, MID/GSLA also identified cell apoptosis-related pathways, suggesting its involvement in cell survival regulation (Fig 5A). GW112 (also known as OLFM4 [42]) is associated with GRIM-19, which is involved in regulating cellular apoptosis [53]. Apart from this, compared to the wild type, GW112 knockdown cells showed a more prominent signal of genomic DNA fragmentation, which is a hallmark of apoptosis [54]. In this case study, compared to the other two widely used annotation tools, our GSLA web tool presents more comprehensive and inspiring annotations for molecular investigators.

### Using the MID/GSLA system to re-analyse the *Piezo1* deficiency mice RNA-seq dataset

Throughout the lifetime of a mammal, natural bone is constantly renewed and remodeled. This complex process involves both the osteoblasts mediated bone formation phase and osteoclasts mediated bone resorption phase [55–57]. The balance between bone formation and resorption is essential for bone health and fracture healing [58]. Bone remodeling is affected by mechanical loading, which is essential for the development of robust weight-bearing bones [59].

To understand the mechanism of how mechanical loading coordinates bone remodeling, Wang et al. generated *Piezo1*-knockout mice and discovered that *Piezo1*-deficiency in osteoblasts cells lead to decreased bone mass, increased bone resorption, and spontaneous fractures after weight bearing [60]. In addition, *Piezo1* deficiency mice displayed a resistance to further bone loss and osteoclast accumulation, suggesting that the PIEZO1 mediated osteoblast-osteoclast crosslink responses to mechanical loads. Mechanistically, *Piezo1* deficiency impaired the production of COL2 and COL9 through decreasing YAP nuclear translocation, which in turn regulates a number of bone matrix proteins including collagens. Their study also suggested that integrins may be a candidate that mediates matrix bridging and osteoclast regulation. Wang et al. performed RNA-Seq of the tibial and femoral cortical bones of the WT and *Piezo1*-knockout mice (GEO database, GSE135282) [60]. Among a total of 19,201 expressed genes, they reported that 560 genes were up-regulated, and 297 genes were down-regulated (fold change > 1.5, p value <0.05) in *Piezo1*-knockout cortical bones.

In this study, the top 250 transcriptionally changed genes were chosen for analysis by DAVID, PANTHER and MID/GSLA. As shown in Fig 5B, the three gene set annotation tools all reported collagen, extracellular matrix, and integrins related biological processes, which are consistent with the terms reported in the original paper (S6–S8 Tables). Apart from these similar results, our MID/GSLA tool uniquely reported mechanical stimulus process related terms, which are intuitively true considering the experiment design (Fig 5B and S8 Table). In addition, MID/GSLA and DAVID reported insulin related pathways. A later study demonstrated that PIEZO1 plays a role in cell swelling induced insulin release [61]. In summary, in this case study, compared to the other two annotation tools, the interpretations made by MID/GSLA are, again, more comprehensive and informative.

## Discussion

As an important disease model, many studies have focused on building the molecular interaction network of *Mus musculus*. To facilitate the hypothesis formulation for molecular investigators, a comprehensive and accurate reference interactome is needed that can serve as a framework to summarize individual gene changes as high-level biological process changes. To date, many mouse interactome databases have been developed. Some of them contain experimentally reported molecular interactions, such as BioGRID [24] and IntAct [25]. Others integrate the predicted interactions, including STRING [11] and MIST [9]. In general, it is considered that experimentally reported interactions are more reliable than interactions that are predicted. However, protein interactions reported in high-throughput experiments are well known to include many false positives. Currently, these high-throughput interactions make the majority of existing interactome databases. In addition, because of the identification method, some *in-vitro* interactions do not have *in-vivo* significance, for example, they are not from the same subcellular compartments in normal physiology.

The negatively correlated accuracy measurements, sensitivity and specificity, are used to evaluate the quality of predicted functional gene associations. An inferred interactome cannot improve its sensitivity and specificity at the same time. Low sensitivity leads to less effective capturing of the true functional interactome, and therefore an insufficient basis for functional annotation of the observed GDE. In contrast, low specificity results in high level of noise in the interactome, leading to a high level of false positive annotations in interactome-based functional annotation of GDEs. Therefore, a high-quality functional gene interactome requires balanced sensitivity and specificity.

On the other hand, available predicted interactions show different sensitivity-specificity characteristics. STRING is a widely used predicted interactome. It has 9,536,624 predicted mouse interactions that are expected to cover a large proportion of mouse protein interactome (52.17%). The fraction of these interactions representing true protein interactions is expected to be low, only 1.06%, as shown in the results section. In contrast, the MID interactome showed balanced coverage and reliability (19.32% coverage and 26.02% reliability if assessed as a protein interaction network), if compared to other existing mice interactomes. Therefore, MID complements existing resources and provides a suitable basis for GSLA annotation of GDEs in mice.

To this date, a variety of tools have been developed for omics data interpretation, including PANTHER [18], KEGG [19], and DAVID [20] etc. Most of them were based on the annotation enrichment strategy. These tools use existing concepts (biological processes or functions) to describe the observed omics changes. However, when the observation (i.e. the actual biological process) cannot be accurately described by an existing concept, these tools tend to report no biological process or very general biological processes, which do not help researchers to understand the data or to suggest directions for further investigation. On the other hand, doing innovative research typically means to explore previously uncharted areas of life mechanisms, where there are no well-established concepts to accurately describe the observed changes.

To meet this challenge, we developed the gene set linkage analysis (GSLA) method, which relies on a functional association network to evaluate whether an observed omics change will collectively interfere with functions of known biological processes. Even when an omics change itself cannot be accurately described by an existing concept; its functional impact may still be described by well-established concepts. The creation of MID enables the application of GSLA for functional impact predictions in mice. The density of functional gene interactions between the component genes in two gene sets can be evaluated by GSLA, which is able to identify significant functional associations between two gene sets. Based on this strategy, we required a high-quality reference interactome in mice with balanced coverage and reliability. The previously developed interactomes cannot serve this purpose, as we discussed above. In this study, two case studies included a mice microarray dataset and a mice RNA-seq dataset were analysed based on the MID (Fig 5). Comparisons were performed between MID/GSLA and other two well documented interpretation tools (DAVID and PANTHER), the results of which inferred a more comprehensive and informative ability of MID/GSLA. In these cases, other enrichment-based tools cannot give instructive annotations, while MID/GSLA can still help researchers to better understand the biological significance of these GDEs. Moreover, the functional association resource provided in MID is a useful reference for investigators to interpret the molecular mechanisms of their genes of interest.

## Supporting information

S1 Fig The receiver operating characteristic curves of 15 feature values. The 15 features with areas under the curve above 0.6 were selected for use in functional gene association prediction.
(JPG)

S1 Table Number of protein interactions and their component proteins collected from IntAct and BioGrid. (PDF)

S2 Table Functional association evidence data and the methods used to compute feature values from these data. (PDF)

S3 Table Assessment of feature qualities. (PDF)

S4 Table Functional annotations reported by MID/GSLA for the top 250 transcriptionally changed genes between the *Olfm4* mutant and wild type. (PDF)

S5 Table Functional annotations reported by DAVID for the top 250 transcriptionally changed genes between the *Olfm4* mutant and wild type. (PDF)

S6 Table Functional annotations reported by GO enrichment analysis tool for the top 250 transcriptionally changed genes between the wild type and *Piezo1-*knockout mice. (PDF)

S7 Table Functional annotations reported by DAVID for the top 250 transcriptionally changed genes between the wild type and *Piezo1*-knockout mice. (PDF)

S8 Table Functional annotations reported by MID/GSLA for the top 250 transcriptionally changed genes between the wild type and *Piezo1*-knockout mice. (PDF)
