# Supplementary figures and images for: Predicted mouse interactome and network-based interpretation of differentially expressed genes
Source: PLoS One. 2022 Apr 7;17(4):e0264174. doi: 10.1371/journal.pone.0264174 (PMC8989236; doi:10.1371/journal.pone.0264174)

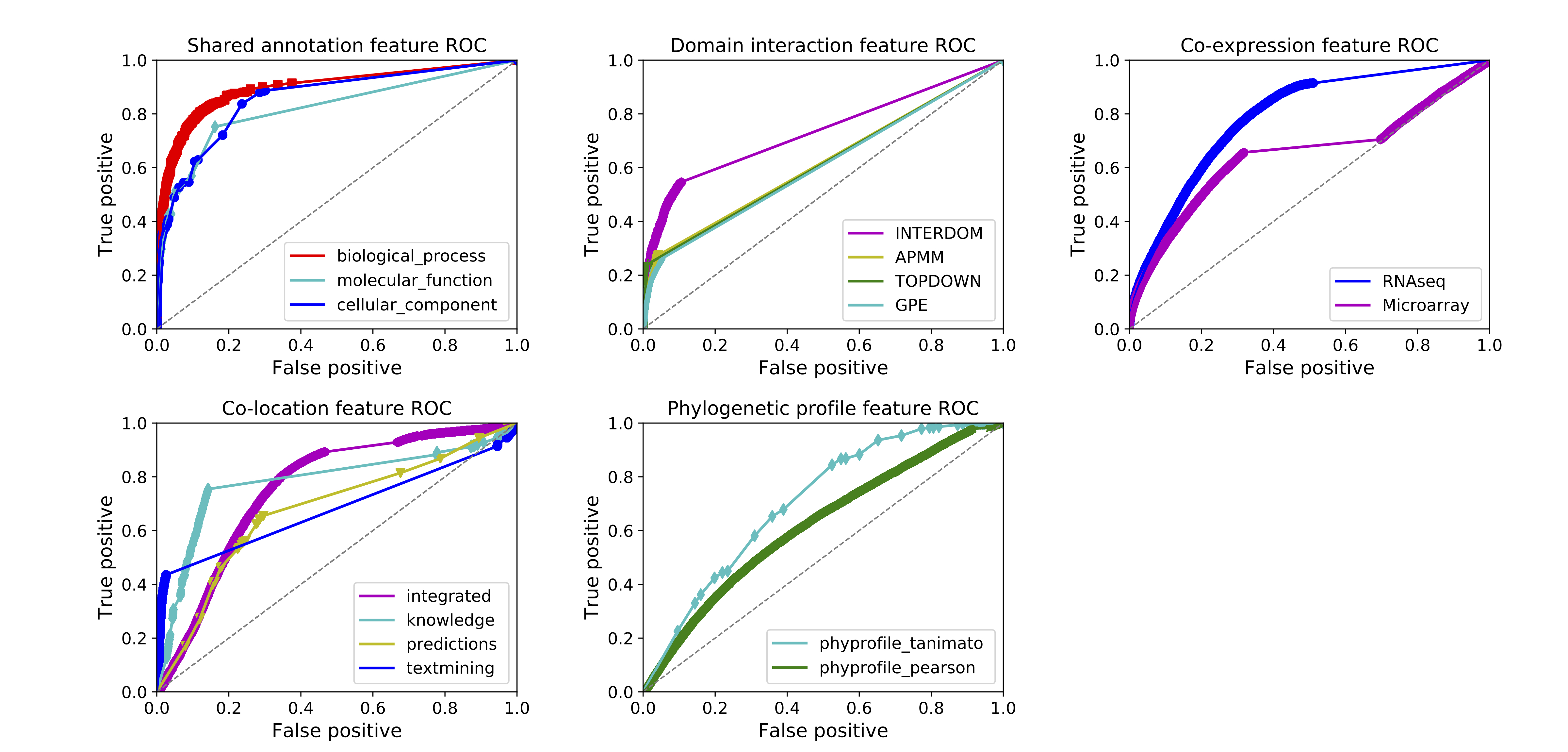

Supplement: S1 Fig — The 15 features with areas under the curve above 0.6 were selected for use in functional gene association prediction. (JPG) [file pone.0264174.s001.jpg]
