# Supplementary material for: Predicted mouse interactome and network-based interpretation of differentially expressed genes
Source: PLoS One. 2022 Apr 7;17(4):e0264174. doi: 10.1371/journal.pone.0264174 (PMC8989236; doi:10.1371/journal.pone.0264174)
Supplement: S1 Table — (PDF) [file pone.0264174.s002.pdf]

Table S1.Number of protein interactions and their component proteins collected from IntAct and BioGrid.

Database	All interactions		High confidence interactions*		Date
	Number of interactions	Number of Proteins	Number of interactions	Number of Proteins	
BioGrid	14277	5499	7234	3629	Dec 25th, 2017
Intact	20508	8811	5259	3164	Dec 12th, 2017
Total	32997	10500	11203	4951	

*High confidence interactions: interactions reported in more than two independent studies, at least one of which is low-throughput experiment.

•
