# Supplementary material for: Predicted mouse interactome and network-based interpretation of differentially expressed genes
Source: PLoS One. 2022 Apr 7;17(4):e0264174. doi: 10.1371/journal.pone.0264174 (PMC8989236; doi:10.1371/journal.pone.0264174)
Supplement: S2 Table — (PDF) [file pone.0264174.s003.pdf]

Table S2. Functional association evidence data and the methods used to compute feature values from these data.

Feature	Data source/ Data set		Statistics
Shared Annotation	GOC	Cellular component<br>Biological process<br>Molecular function	Maximum Shared Annotation Score
Homologous Interactions	Inparanoid Score		Maximum
Domain Interaction	IDDI	3DID<br>IPFAM<br>PINS<br>TW<br>HIMAP<br>DOMAINGA<br>PVALUE<br>IPPRI<br>RCDP<br>DIPD<br>RDFF<br>DPEA<br>ME<br>PE<br>DIMA_STRING<br>KGIDDI<br>LLZ<br>GPE<br>DIMA_DPROF<br>APMM<br>TOPDOWN<br>DIMA_DPEA<br>INTERDOM	Maximum Shared Domain Score
Co-localization	Compartments	Knowledge channel<br>Experiments channel<br>Text mining channel<br>Predictions channel	Cosine Similarity Score
Co-expression	COXPRESdb	Microarray<br>RNASeq	Pearson's Correlation Score
Phylogenetic Profile	DIOPT		Mutual Information Score<br>Pearson's Correlation Score<br>Tanimato Correlation Score
