# Supplementary material for: Predicted mouse interactome and network-based interpretation of differentially expressed genes
Source: PLoS One. 2022 Apr 7;17(4):e0264174. doi: 10.1371/journal.pone.0264174 (PMC8989236; doi:10.1371/journal.pone.0264174)
Supplement: S3 Table — (PDF) [file pone.0264174.s004.pdf]

Table S3. Assessment of feature qualities.

Evidence	type Feature	AUC of ROC	AUC > 0.6
Shared Annotation	biological_process_value	0.898	Yes
	molecular_function_value	0.821	Yes
	cellular_component_value	0.858	Yes
Coexpression	RNAseq_value	0.788	Yes
	Microarray_value	0.648	Yes
Phylogenetic profile	phyprofile_tanimato_valu	0.706	Yes
	phyprofile_pearson_value	0.620	Yes
	phyprofile_mutual_value	0.535	No
Colocation	integrated_value	0.755	Yes
	knowledge_value	0.782	Yes
	predictions_value	0.675	Yes
	textmining_value	0.679	Yes
Domain Interactions	INTERDOM_value	0.732	Yes
	APMM_value	0.618	Yes
	TOPDOWN_value	0.614	Yes
	GPE_value	0.608	Yes
	PINS_value	0.549	No
	LLZ_value	0.513	No
	RDFF_value	0.547	No
	DIPD_value	0.530	No
	3DID_value	0.573	No
	IPPRI_value	0.525	No
	ME_value	0.575	No
	KGIDDI_value	0.580	No
	IPFAM_value	0.556	No
	DOMAINGA_value	0.504	No
	RCDP_value	0.516	No
	DIMA_DPEA_value	0.597	No
	DIMA_DPROF_value	0.531	No
	PVALUE_value	0.513	No
	TW_value	0.504	No
	HIMAP_value	0.516	No
	PE_value	0.547	No
	DIMA_STRING_value	0.547	No
	DPEA_value	0.514	No
Homologous interactions	inparanoid_value	0.591	No
