# Supplementary material for: Predicted mouse interactome and network-based interpretation of differentially expressed genes
Source: PLoS One. 2022 Apr 7;17(4):e0264174. doi: 10.1371/journal.pone.0264174 (PMC8989236; doi:10.1371/journal.pone.0264174)
Supplement: S4 Table — (PDF) [file pone.0264174.s005.pdf]

Table S4. Functional annotations reported by MID/GSLA for the top 250 transcriptionally changed genes between the Olfm4 mutant and wild type.

Job Number: GSLA08236562515329

Cutoff used:

Q1: density >= 0.01

Q2: p <= 0.001

Meaning:

Q1: inter-geneset interaction density is greater than expected.

Q2: the observed interaction density can only be observed in the biologically correct interactome topology.

Categories selected: GO Panther pathway Reactome pathway

Species: Mus musculus

Interaction dataset selected: MID

#Subjob Number: GSLA08236562515329_0

#Description: #TOP-250-up

#Quaried GeneSet: Pnliprp1,Pnliprp2,Spag1,Pnliprp2,BC018473,Phyhip,4930480K23Rik,Cry1,Nudt18,Fam160b2,Sdc2,Tmem263,Med1,Hal,Dtd2,Rb1,St6galnac2,Hist3h2a,Cpq,St6galnac1,Epn2,2610044O15Rik8,Gja1,Nt5dc3,Hist3h2bb-ps///Hist3h2ba,Ntn4,D030063E12,Mtmr9,Lipa,Ctr9,9230110C19Rik,Lta4h,Pum3,Fst,Hbp1,Nt5dc3,Snhg18,Gnai3,Trim29,A230046K03Rik,Derl1,Ank3,6030458C11Rik,Sf3b5,Lipa,Usp45,Dsc1,Fam83g,1110059E24Rik,Ccdc3,Lipa,1810064F22Rik,D17892,Gfra2,Gja1,Grhl2,Prkca,Pwp1,Rassf9,Rp2,1190002N15Rik,A930033H14Rik,Atp10a,Cfap54,G2e3,Atp10a,Cfap54,Riok1,Steap2,Prr5l,Timm44,Tmem18,Zfp455,Kitl,Cpe,Aldh18a1,Aldh1l2,Clec3b,Trim30a,Cldn1,Spag9,Actr6,Idi1,Mtmr9,Ap4s1,Lgals7,Lsm12,Lrrn4,Zfp800,Trim12a,Ces2f,Trpc6,Zfp944,Rock1,Usp53,Cpd,1110059G02Rik///Sntb1,A430103D13Rik,Tchh,Dnajc3,Hist3h2a,Sdc2,2010107G23Rik,2610044O15Rik8,Pdzd8,Qk,Slc11a2,Wdr95,Crim1,Zfx,Ikbkg,Ormdl1,Slc48a1,Clock,Dyrk2,Trim34a,Yipf4,Cdc42,Fam91a1,Rai14,Reep3,Rnf187,Slc16a11,Enpp1,Mtmr9,Phf6,Rcbtb2,9930024M15Rik,Ckap4,Fbxo11,Lypd8,A930033H14Rik,Ankrd26,Atp7a,Sgms2,Spin4,Vav3,Gm16794,Kdm5d,Epsti1,Ppp1cb,C1qtnf3,Dmxl1,Fbn1,Gm38524///Xkr6,Prkar1b,St6galnac1,Vldlr,Prr5l,Sdc1,Zfp277,Ckap4,Ptpdc1,Whsc1,Hbs1l,Igfbp6,Txlnb,2210409E12Rik///Coil,Pgr,Rcbtb2,Tbc1d15,Anxa13,Dmkn,Gm41724,Usp45,Atxn7l1,Ankrd10,Crym,Idh1,Marcks,Mgat2,Plcxd2,Plxdc1,Tnks2,Mpp6,Prg4,Trim5,Pias2,Tm7sf3,Npl,Trim25,2900011G08Rik,Herpud2,Pcsk5,Rassf9,Rcor1,Zfp800,Atp10a,Ednrb,Plekha3,Tpbg,Gca,Gtpbp4,Chrdl1,Mfap3l,4930512M02Rik,LOC100862324///Fam205a3///LOC100862043///Fam205a4///Fam205a2///Fam205a1,Npas2,Smim10l1,Trim30d,Trpm3,Usp6nl,1810014B01Rik,4930592A05Rik,Acsl4,AF529169,Cbln3,Dpy19l4,Gfra2,L3hypdh,Lce3c,Mtdh,Proser2,Prss23,Scpep1,Soat1,Tspan13,Wdr77,Abhd3,Cdyl,Gtf2e1,Pou6f1,Prss28,Stt3a,Timp3,1200007C13Rik,4930448K20Rik,Hist1h4m///Hist1h4b///Hist1h4a///Hist4h4///Hist1h4n///Hist1h4k///Hist1h4j///Hist1h4i///Hist1h4f///Hist1h4d///Hist1h4c///Hist2h4///Hist1h4h,Myh9,Psmc6,Rab2a,Lats2,Lepr,Rnf225,Thoc1,Tspyl1,1110059G02Rik///Sntb1,Maf,Ppp1cb,Srsf7,LOC102643254///Rps6-ps4///Rps6,Marcks,Tubb5,Bicd1,Esm1,Kdm1b,Klhl7,LOC102643254///Rps6-ps4///Rps6,Mmp19,Mtm1

<table>
<tr><th>Term type</th><th>Term</th><th>Description</th><th>Term size</th><th>P value</th><th>Density</th><th>Interaction number</th><th>Overlap gene number</th><th>Overlap gene(s)</th><th>Interactions</th></tr>
<tr><td>GO biological process</td><td>GO:0002934</td><td>desmosome organization</td><td>10</td><td>5.00E-04</td><td>0.01</td><td>21</td><td>1</td><td>Prkca</td><td>Myh9-MGI:96650|Mtm1-MGI:109611|Med1-MGI:1096393|Ank3-MGI:96650|Ank3-MGI:109611|Ank3-MGI:1914701|Ank3-MGI:1196466|Cldn1-MGI:1096393|Cldn1-MGI:1914701|Gja1-MGI:1096393|Gja1-MGI:96650|Gja1-MGI:109611|Gja1-MGI:1914701|Clock-MGI:1096393|Clock-Prkca|Pgr-MGI:1096393|Prkca-MGI:1096393|Prkca-MGI:1891830|Rock1-MGI:1096393|Rock1-Prkca|Rock1-MGI:1914701</td></tr>
<tr><td>GO biological process</td><td>GO:0016264</td><td>gap junction assembly</td><td>6</td><td>0</td><td>0.01031</td><td>13</td><td>0</td><td></td><td>Pcsk5-MGI:88274|Cdc42-MGI:88274|Ank3-MGI:95716|Ank3-MGI:88274|Ank3-MGI:95718|Ank3-MGI:1914701|Cldn1-MGI:1914701|Gja1-MGI:88274|Gja1-MGI:95718|Gja1-MGI:1914701|Gja1-MGI:95720|Rock1-MGI:88274|Rock1-MGI:1914701</td></tr>
<tr><td>GO biological process</td><td>GO:0030865</td><td>cortical cytoskeleton organization</td><td>31</td><td>5.00E-04</td><td>0.01182</td><td>77</td><td>1</td><td>Rock1</td><td>Cdc42-MGI:1337100|Cdc42-MGI:1345964|Cdc42-MGI:1335094|Cdc42-MGI:107231|Cdc42-MGI:109520|Cdc42-MGI:1349423|Cdc42-MGI:99435|Cdc42-MGI:101765|Cdc42-MGI:98931|Myh9-MGI:1337100|Myh9-MGI:1099832|Myh9-MGI:2137706|Myh9-MGI:104809|Myh9-MGI:1335094|Myh9-MGI:109520|Myh9-Rock1|Myh9-MGI:107926|Myh9-MGI:99435|Myh9-MGI:98931|Lats2-MGI:109520|Lats2-Rock1|Lats2-MGI:107926|Rab2a-MGI:1931553|Vav3-MGI:1337100|Vav3-MGI:1860485|Vav3-MGI:2137706|Vav3-MGI:1926945|Vav3-MGI:1345964|Vav3-MGI:104809|Vav3-MGI:107231|Vav3-MGI:109520|Vav3-Rock1|Vav3-MGI:107926|Vav3-MGI:1349423|Vav3-MGI:101765|Ank3-MGI:1860485|Ank3-MGI:2137706|Ank3-MGI:107231|Ank3-MGI:107926|Ank3-MGI:101765|Tubb5-MGI:98931|Spag9-MGI:99435|Marcks-MGI:2137706|Cldn1-Rock1|Gnai3-MGI:107231|Gja1-MGI:2137706|Gja1-Rock1|Gja1-MGI:107926|Trpc6-MGI:1926945|Trpc6-MGI:2179435|Thoc1-MGI:1338071|Clock-MGI:1338071|Rai14-MGI:107926|Dnajc3-MGI:88252|Ikbkg-MGI:1338071|Ikbkg-MGI:104798|Pgr-MGI:107926|Pgr-MGI:1338071|Pgr-MGI:101765|Prkca-Rock1|Prkca-MGI:107926|Prkca-MGI:101765|Rock1-MGI:103008|Rock1-MGI:1337100|Rock1-MGI:1099832|Rock1-MGI:1860485|Rock1-MGI:2137706|Rock1-MGI:1926945|Rock1-MGI:1345964|Rock1-MGI:104809|Rock1-MGI:107231|Rock1-MGI:109520|Rock1-MGI:107926|Rock1-MGI:1338071|Rock1-MGI:99435|Rock1-MGI:98931|Fst-MGI:104798</td></tr>
</table>

<table>
<tr><th></th><th></th><th></th><th></th><th></th><th></th><th></th><th></th><th></th><th></th></tr>
<tr><td>GO biological process</td><td>GO:0033142</td><td>progesterone receptor binding</td><td>4</td><td>0</td><td>0.02261</td><td>19</td><td>0</td><td></td><td>Cry1-MGI:1276533|Pias2-MGI:1276533|Pias2-MGI:1201691|Pias2-MGI:1316652|Npas2-MGI:1276533|Npas2-MGI:1276523|Med1-MGI:1276533|Med1-MGI:1201691|Med1-MGI:1316652|Rb1-MGI:1316652|Mtdh-MGI:1201691|Lepr-MGI:1276533|Lepr-MGI:1316652|Clock-MGI:1276533|Clock-MGI:1201691|Clock-MGI:1276523|Ikbkg-MGI:1201691|Pgr-MGI:1276533|Pgr-MGI:1276523</td></tr>
<tr><td>GO biological process</td><td>GO:0043296</td><td>apical junction complex</td><td>19</td><td>6.00E-04</td><td>0.01027</td><td>41</td><td>0</td><td></td><td>Cdc42-MGI:88276|Cdc42-MGI:1096342|Cdc42-MGI:1314653|Myh9-MGI:1351655|Myh9-MGI:88354|Myh9-MGI:1927237|Myh9-MGI:1096342|Fbn1-MGI:98759|Pias2-MGI:88276|Zfx-MGI:88276|Med1-MGI:88276|Vav3-MGI:98759|Vav3-MGI:1351655|Vav3-MGI:1314653|Ank3-MGI:98759|Ank3-MGI:88354|Ank3-MGI:88276|Ctr9-MGI:88276|Tubb5-MGI:1096342|Maf-MGI:88276|Cldn1-MGI:98759|Cldn1-MGI:88354|Cldn1-MGI:1927237|Cldn1-MGI:1314653|Rb1-MGI:88276|Gja1-MGI:98759|Gja1-MGI:88354|Gja1-MGI:88276|Mtdh-MGI:88276|Gtf2e1-MGI:88276|Ednrb-MGI:88276|Ednrb-MGI:1096342|Kitl-MGI:88276|Ikbkg-MGI:1096342|Prkca-MGI:109211|Rock1-MGI:98759|Rock1-MGI:1351655|Rock1-MGI:1927237|Rock1-MGI:88276|Rock1-MGI:1096342|Rock1-MGI:1314653</td></tr>
<tr><td>GO biological process</td><td>GO:0051016</td><td>barbed-end actin filament capping</td><td>10</td><td>0.001</td><td>0.01095</td><td>23</td><td>0</td><td></td><td>Cdc42-MGI:104684|Cdc42-MGI:700006|Cdc42-MGI:104652|Myh9-MGI:97887|Myh9-MGI:104684|Myh9-MGI:1100520|Myh9-MGI:700006|Myh9-MGI:106227|Myh9-MGI:1346078|Myh9-MGI:87918|Myh9-MGI:104652|Myh9-MGI:87919|Vav3-MGI:104684|Ank3-MGI:97887|Ank3-MGI:104684|Marcks-MGI:87918|Marcks-MGI:104652|Gca-MGI:104652|Rock1-MGI:97887|Rock1-MGI:104684|Rock1-MGI:1100520|Rock1-MGI:87918|Rock1-MGI:87919</td></tr>
<tr><td>GO biological process</td><td>GO:0086073</td><td>bundle of His cell-Purkinje myocyte adhesion involved in cell communication</td><td>6</td><td>7.00E-04</td><td>0.01031</td><td>13</td><td>0</td><td></td><td>Myh9-MGI:96650|Mtm1-MGI:109611|Ank3-MGI:96650|Ank3-MGI:109611|Ank3-MGI:1914701|Ank3-MGI:1196466|Ank3-MGI:2661445|Cldn1-MGI:1914701|Gja1-MGI:96650|Gja1-MGI:109611|Gja1-MGI:1914701|Gja1-MGI:2661445|Rock1-MGI:1914701</td></tr>
<tr><td>GO biological process</td><td>GO:1901890</td><td>positive regulation of cell junction assembly</td><td>33</td><td>8.00E-04</td><td>0.01024</td><td>71</td><td>2</td><td>Cldn1|Rock1</td><td>Pcsk5-MGI:98957|Pcsk5-MGI:96683|Pcsk5-MGI:1349164|Pcsk5-MGI:98664|Cdc42-MGI:101757|Cdc42-MGI:1352757|Cdc42-MGI:97845|Cdc42-MGI:87859|Myh9-MGI:106206|Myh9-MGI:101757|Myh9-MGI:1352757|Myh9-MGI:97845|Myh9-Rock1|Lats2-Rock1|Pias2-MGI:1201674|Vav3-MGI:1352757|Vav3-MGI:98747|Vav3-MGI:97845|Vav3-Rock1|Vav3-MGI:1858233|Vav3-MGI:98664|Ank3-MGI:102709|Ank3-MGI:98664|Sdc1-MGI:1349164|Marcks-MGI:101757|Maf-MGI:1201674|Sdc2-MGI:1349164|Cldn1-MGI:103006|Cldn1-MGI:106206|Cldn1-MGI:2384210|Cldn1-MGI:101815|Cldn1-MGI:98747|Cldn1-Rock1|Cldn1-MGI:1917258|Cldn1-MGI:1858233|Cldn1-MGI:98664|Rb1-MGI:87859|Rb1-MGI:1201674|Gja1-Cldn1|Gja1-MGI:97845|Gja1-MGI:102709|Gja1-Rock1|Gja1-MGI:1349164|Gja1-MGI:1858233|Ednrb-MGI:97845|Ednrb-MGI:87963|Clock-MGI:1201674|Gfra2-MGI:1352757|Kitl-MGI:103178|Pgr-MGI:98957|Pgr-MGI:1201674|Prkca-Rock1|Rock1-MGI:103006|Rock1-MGI:1339468|Rock1-MGI:106206|Rock1-Cldn1|Rock1-MGI:101757|Rock1-MGI:2384210|Rock1-MGI:101815|Rock1-MGI:1349394|Rock1-MGI:1352757|Rock1-MGI:97845|Rock1-MGI:103178|Rock1-MGI:1195263|Rock1-MGI:96683|Rock1-MGI:1349164|Rock1-MGI:1858233|Rock1-MGI:95278|Rock1-MGI:87859|Rock1-MGI:1201674|Rock1-MGI:98664</td></tr>
</table>

<table>
<tr><td>GO biological process</td><td>GO:1903393</td><td>positive regulation of adherens junction organization</td><td>31</td><td>0</td><td>0.01029</td><td>67</td><td>1</td><td>Rock1</td><td>Pcsk5-MGI:98957|Pcsk5-MGI:96683|Pcsk5-MGI:1349164|Pcsk5-MGI:98664|Cdc42-MGI:101757|Cdc42-MGI:1352757|Cdc42-MGI:97845|Cdc42-MGI:99435|Cdc42-MGI:87859|Myh9-MGI:106206|Myh9-MGI:101757|Myh9-MGI:87918|Myh9-MGI:1352757|Myh9-MGI:97845|Myh9-Rock1|Myh9-MGI:99435|Lats2-Rock1|Pias2-MGI:1201674|Vav3-MGI:1352757|Vav3-MGI:98747|Vav3-MGI:97845|Vav3-Rock1|Vav3-MGI:106271|Vav3-MGI:98664|Ank3-MGI:98664|Spag9-MGI:99435|Sdc1-MGI:1349164|Marcks-MGI:101757|Marcks-MGI:87918|Maf-MGI:1201674|Sdc2-MGI:1349164|Cldn1-MGI:103006|Cldn1-MGI:106206|Cldn1-MGI:101815|Cldn1-MGI:98747|Cldn1-Rock1|Cldn1-MGI:98664|Rb1-MGI:87859|Rb1-MGI:1201674|Gja1-MGI:97845|Gja1-Rock1|Gja1-MGI:1349164|Ednrb-MGI:97845|Clock-MGI:1201674|Gfra2-MGI:1352757|Kitl-MGI:103178|Pgr-MGI:98957|Pgr-MGI:1201674|Prkca-Rock1|Rock1-MGI:103006|Rock1-MGI:1339468|Rock1-MGI:106206|Rock1-MGI:101757|Rock1-MGI:101815|Rock1-MGI:1349394|Rock1-MGI:87918|Rock1-MGI:1352757|Rock1-MGI:97845|Rock1-MGI:103178|Rock1-MGI:1195263|Rock1-MGI:99435|Rock1-MGI:96683|Rock1-MGI:1349164|Rock1-MGI:106271|Rock1-MGI:87859|Rock1-MGI:98664|Rock1-MGI:1201674</td></tr>
<tr><td>GO biological process</td><td>GO:2000810</td><td>regulation of bicellular tight junction assembly</td><td>18</td><td>0</td><td>0.0164</td><td>62</td><td>3</td><td>Cldn1|Gja1|Rock1</td><td>Cdc42-MGI:106612|Myh9-Rock1|Myh9-MGI:107926|Myh9-MGI:106612|Lats2-Rock1|Lats2-MGI:107926|Zfx-MGI:98330|Med1-MGI:1096393|Vav3-MGI:97600|Vav3-Rock1|Vav3-MGI:107926|Vav3-MGI:1858233|Vav3-MGI:106612|Ank3-Gja1|Ank3-MGI:107926|Ank3-MGI:1914701|Ank3-MGI:106612|Cldn1-MGI:1096393|Cldn1-Gja1|Cldn1-MGI:2384210|Cldn1-Rock1|Cldn1-MGI:1858233|Cldn1-MGI:1914701|Cldn1-MGI:106612|Gja1-MGI:1096393|Gja1-Cldn1|Gja1-MGI:98330|Gja1-Rock1|Gja1-MGI:107926|Gja1-MGI:1858233|Gja1-MGI:1914701|Gja1-MGI:106612|Ormdl1-MGI:97592|Thoc1-MGI:1338071|Clock-MGI:1096393|Clock-MGI:98330|Clock-MGI:1338071|Rai14-MGI:107926|Ikbkg-MGI:1338071|Ikbkg-MGI:104798|Pgr-MGI:1096393|Pgr-MGI:98330|Pgr-MGI:107926|Pgr-MGI:1338071|Prkca-MGI:1096393|Prkca-MGI:97600|Prkca-Rock1|Prkca-MGI:107926|Prkca-MGI:97592|Rock1-MGI:1096393|Rock1-Cldn1|Rock1-Gja1|Rock1-MGI:97600|Rock1-MGI:2384210|Rock1-MGI:107926|Rock1-MGI:1338071|Rock1-MGI:1858233|Rock1-MGI:1914701|Rock1-MGI:95278|Rock1-MGI:106612|Rock1-MGI:97592|Fst-MGI:104798</td></tr>
<tr><td>Panther pathway</td><td>P00025</td><td>Hedgehog signaling pathway</td><td>16</td><td>0</td><td>0.01458</td><td>49</td><td>1</td><td>Prkar1b</td><td>Pcsk5-MGI:108075|Cdc42-MGI:95729|Cdc42-MGI:95727|Cdc42-MGI:1861437|Cdc42-MGI:104878|Cry1-MGI:1351660|Cry1-MGI:1338871|Myh9-MGI:1861437|Myh9-MGI:104878|Lats2-MGI:1861437|Lats2-MGI:1098280|Pias2-MGI:1861437|Pias2-MGI:1098280|Zfx-MGI:95727|Zfx-MGI:1098280|Med1-MGI:95727|Med1-MGI:1338871|Med1-MGI:1098280|Hist3h2a-MGI:1098280|Dyrk2-MGI:1861437|Ank3-MGI:1861437|Maf-MGI:1098280|Prkar1b-MGI:108025|Prkar1b-MGI:97760|Prkar1b-MGI:104878|Rb1-MGI:1351660|Rb1-MGI:1098280|Gja1-MGI:108075|Gtf2e1-MGI:1098280|Cdyl-MGI:1098280|Ednrb-MGI:95729|Ednrb-MGI:1861437|Clock-MGI:1351660|Clock-MGI:1338871|Clock-MGI:1098280|Kitl-MGI:108075|Ikbkg-MGI:1861437|Ikbkg-MGI:1338871|Pgr-MGI:95729|Pgr-MGI:95727|Pgr-MGI:1861437|Pgr-MGI:1338871|Pgr-MGI:1098280|Pgr-MGI:105373|Prkca-MGI:1861437|Rock1-MGI:1351660|Rock1-MGI:1861437|Rock1-MGI:104878|Rock1-MGI:105373</td></tr>
</table>

<table>
<tr><td>Reactome pathway</td><td>R-MMU-111465</td><td>Apoptotic cleavage of cellular proteins</td><td>36</td><td>1.00E-04</td><td>0.01097</td><td>83</td><td>1</td><td>Rock1</td><td>Cdc42-MGI:97601|Cdc42-MGI:95481|Cdc42-MGI:88276|Cdc42-MGI:2385007|Cdc42-MGI:700006|Cdc42-MGI:98932|Myh9-MGI:87918|Myh9-MGI:95481|Myh9-MGI:1277961|Myh9-MGI:95851|Myh9-MGI:88354|Myh9-Rock1|Myh9-MGI:2385007|Myh9-MGI:700006|Mtm1-MGI:97180|Mtm1-MGI:1277961|Mtm1-MGI:109611|Mtm1-MGI:98932|Lats2-Rock1|Fbn1-MGI:98759|Fbn1-MGI:1341872|Pias2-MGI:88276|Zfx-MGI:88276|Med1-MGI:88276|Enpp1-MGI:95481|Vav3-MGI:98759|Vav3-MGI:1101778|Vav3-MGI:95481|Vav3-MGI:1277961|Vav3-MGI:1341872|Vav3-Rock1|Vav3-MGI:2385007|Ank3-MGI:98759|Ank3-MGI:1196466|Ank3-MGI:95481|Ank3-MGI:1277961|Ank3-MGI:88276|Ank3-MGI:1341872|Ank3-MGI:88354|Ank3-MGI:2385007|Ank3-MGI:109611|Ctr9-MGI:88276|Marcks-MGI:87918|Maf-MGI:88276|Cldn1-MGI:98759|Cldn1-MGI:106183|Cldn1-MGI:1341872|Cldn1-MGI:88354|Cldn1-Rock1|Derl1-MGI:1350933|Srsf7-MGI:1891824|Rb1-MGI:88276|Rb1-MGI:105084|Rb1-MGI:96795|Gja1-MGI:98759|Gja1-MGI:97598|Gja1-MGI:88276|Gja1-MGI:96794|Gja1-MGI:88354|Gja1-Rock1|Gja1-MGI:109611|Mtdh-MGI:88276|Mtdh-MGI:1197009|Gtf2e1-MGI:88276|Ednrb-MGI:88276|Kitl-MGI:88276|Dnajc3-MGI:1350933|Ikbkg-MGI:1197009|Ikbkg-MGI:107739|Pgr-MGI:97601|Pgr-MGI:105084|Prkca-MGI:97601|Prkca-MGI:97598|Prkca-Rock1|Rock1-MGI:98759|Rock1-MGI:97601|Rock1-MGI:87918|Rock1-MGI:95481|Rock1-MGI:97598|Rock1-MGI:88276|Rock1-MGI:1341872|Rock1-MGI:109383|Rock1-MGI:107739</td></tr>
<tr><td>Reactome pathway</td><td>R-MMU-351906</td><td>Apoptotic cleavage of cell adhesion proteins</td><td>11</td><td>3.00E-04</td><td>0.01558</td><td>36</td><td>0</td><td></td><td>Cdc42-MGI:88276|Myh9-MGI:88354|Mtm1-MGI:109611|Fbn1-MGI:1341872|Fbn1-MGI:98759|Pias2-MGI:88276|Zfx-MGI:88276|Med1-MGI:88276|Vav3-MGI:1341872|Vav3-MGI:98759|Ank3-MGI:1341872|Ank3-MGI:98759|Ank3-MGI:88354|Ank3-MGI:88276|Ank3-MGI:109611|Ank3-MGI:1196466|Ctr9-MGI:88276|Maf-MGI:88276|Cldn1-MGI:1341872|Cldn1-MGI:98759|Cldn1-MGI:88354|Cldn1-MGI:106183|Rb1-MGI:88276|Gja1-MGI:98759|Gja1-MGI:88354|Gja1-MGI:88276|Gja1-MGI:109611|Mtdh-MGI:88276|Gtf2e1-MGI:88276|Ednrb-MGI:88276|Kitl-MGI:88276|Ikbkg-MGI:107739|Rock1-MGI:1341872|Rock1-MGI:98759|Rock1-MGI:107739|Rock1-MGI:88276</td></tr>
</table>
