# Supplementary material for: Predicted mouse interactome and network-based interpretation of differentially expressed genes
Source: PLoS One. 2022 Apr 7;17(4):e0264174. doi: 10.1371/journal.pone.0264174 (PMC8989236; doi:10.1371/journal.pone.0264174)
Supplement: S5 Table — (PDF) [file pone.0264174.s006.pdf]

Table S5.Functional annotations reported by DAVID for the top 250 transcriptionally changed genes between the Olfm4 mutant and wild type.

Annotation Cluster 1 Enrichment Score: 4.200673450581423

Category	Term	Count	%	PValue	Genes	List Total	Pop Hits	Pop Total	Fold Enrichment	Bonferroni	Benjamini	FDR
UP_KEYWORDS	Metal-binding	54	24.88479263	2.12E-05	ATP10A, NT5DC3, RNF187, LATS2, KDM1B, PGR, GTF2E1, TRIM5, G2E3, KDM5D, TCHH, PRKCA, NUDT18, ROCK1, MMP19, ZFX, PPP1CB, RNF225, TNKS2, TRIM12A, CPE, PIAS2, CPD, STEAP2, FBXO11, 2610044O15RIK8, GNAI3, ENPP1, CPQ, RIOK1, TIMP3, ZFP455, TRIM30A, NPAS2, TRIM30D, IDH1, PNLIPRP1, PNLIPRP2, VAV3, TRIM29, TRIM34A, WHSC1, TRIM25, ATP7A, PDZD8, SRSF7, ZFP944, IKBKG, ZFP800, LTA4H, IDI1, USP45, GCA, PHF6	203	3395	22680	1.777055508	0.004807775	0.00240678	0.027157897
GOTERM_MF_DIRECT	GO:0008270~zinc ion binding	26	11.98156682	2.17E-05	ENPP1, RNF187, SLC11A2, PGR, KDM1B, TRIM30A, TRIM5, G2E3, TRIM30D, KDM5D, PRKCA, TRIM29, TRIM34A, MMP19, WHSC1, TRIM25, RNF225, TRIM12A, SRSF7, CPE, PIAS2, LTA4H, CPD, USP45, FBXO11, PHF6	164	1075	17446	2.572864436	0.008233055	0.00823305	0.030046297
UP_KEYWORDS	Zinc	38	17.51152074	4.34E-05	2610044O15RIK8, ENPP1, CPQ, RNF187, TIMP3, ZFP455, KDM1B, PGR, TRIM5, TRIM30A, GTF2E1, G2E3, TRIM30D, KDM5D, PRKCA, VAV3, ROCK1, TRIM29, TRIM34A, MMP19, ZFX, TRIM25, WHSC1, RNF225, TNKS2, PDZD8, TRIM12A, ZFP944, CPE, SRSF7, IKBKG, ZFP800, PIAS2, LTA4H, CPD, USP45, PHF6, FBXO11	203	2099	22680	2.022638038	0.009794031	0.00327539	0.055455086
GOTERM_MF_DIRECT	GO:0046872~metal ion binding	52	23.96313364	1.52E-04	ATP10A, NT5DC3, RNF187, LATS2, KDM1B, PGR, GTF2E1, G2E3, ZFP277, KDM5D, PRKCA, NUDT18, ROCK1, MMP19, ZFX, PPP1CB, RNF225, TNKS2, TRIM12A, CPE, PIAS2, CPD, STEAP2, FBXO11, 2610044O15RIK8, GNAI3, ENPP1, CPQ, RIOK1, TIMP3, ZFP455, TRIM30A, NPAS2, IDH1, PNLIPRP1, PNLIPRP2, VAV3, TRIM29, TRIM34A, WHSC1, TRIM25, ATP7A, PDZD8, SRSF7, ZFP944, IKBKG, ZFP800, LTA4H, IDI1, USP45, GCA, PHF6	164	3355	17446	1.648780488	0.056115093	0.02846261	0.209703317
UP_KEYWORDS	Zinc-finger	29	13.3640553	3.28E-04	2610044O15RIK8, RNF187, ZFP455, KDM1B, PGR, GTF2E1, TRIM30A, TRIM5, G2E3, TRIM30D, KDM5D, PRKCA, VAV3, ROCK1, TRIM29, TRIM34A, ZFX, TRIM25, WHSC1, RNF225, TRIM12A, SRSF7, ZFP944, IKBKG, ZFP800, PIAS2, USP45, FBXO11, PHF6	203	1565	22680	2.07028754	0.071716919	0.01843264	0.41853874

Annotation Cluster 2 Enrichment Score: 2.275954974106878

Category	Term	Count	%	PValue	Genes	List Total	Pop Hits	Pop Total	Fold Enrichment	Bonferroni	Benjamini	FDR
INTERPRO	IPR000315:Zinc finger, B-box	7	3.225806452	4.82E-05	TRIM5, TRIM30A, TRIM12A, TRIM30D, TRIM29, TRIM34A, TRIM25	190	71	20594	10.68628614	0.023657615	0.02365762	0.069288315
SMART	SM00336:BBOX	6	2.764976959	5.66E-04	TRIM5, TRIM30A, TRIM12A, TRIM30D, TRIM29, TRIM34A	117	61	10425	8.764186633	0.073628324	0.07362832	0.661966811
INTERPRO	IPR017907:Zinc finger, RING-type, conserved site	8	3.686635945	7.82E-04	TRIM5, TRIM30A, TRIM12A, TRIM30D, TRIM34A, TRIM25, RNF187, RNF225	190	163	20594	5.31972877	0.322268545	0.17675553	1.119855685
INTERPRO	IPR013083:Zinc finger, RING/FYVE/PHD-type	13	5.99078341	8.70E-04	TRIM5, GTF2E1, TRIM30A, G2E3, TRIM12A, TRIM30D, TRIM34A, WHSC1, TRIM25, RNF187, USP45, RNF225, KDM5D	190	445	20594	3.166434063	0.351186511	0.13428829	1.24460281
INTERPRO	IPR003879:Butyrophylin-like	5	2.304147465	0.00185725	TRIM5, TRIM30A, TRIM30D, TRIM34A, TRIM25	190	57	20594	9.507848569	0.603036646	0.20624293	2.639283782

INTERPRO	IPR001841:Zinc finger, RING-type	9	4.147465438	0.00502377	TRIM5, TRIM30A, TRIM12A, TRIM30D, TRIM34A, WHSC1, TRIM25, RNF187, RNF225	190	286	20594	3.410857563	0.918169609	0.39384607	6.990201457
INTERPRO	IPR003877:SPla/RYanodine receptor SPRY	5	2.304147465	0.00552181	TRIM5, TRIM30A, TRIM30D, TRIM34A, TRIM25	190	77	20594	7.038277512	0.936196323	0.36786823	7.657826789
INTERPRO	IPR001870:B30.2/SPRY domain	5	2.304147465	0.007187108	TRIM5, TRIM30A, TRIM30D, TRIM34A, TRIM25	190	83	20594	6.529486367	0.972260296	0.36116643	9.857925701
UP_SEQ_FEATURE	zinc finger region:B box-type	4	1.843317972	0.0085946	TRIM30A, TRIM12A, TRIM29, TRIM34A	182	42	18012	9.425431711	0.998149951	0.99814995	12.26763142
SMART	SM00449:SPRY	5	2.304147465	0.009646492	TRIM5, TRIM30A, TRIM30D, TRIM34A, TRIM25	117	75	10425	5.94017094	0.729801482	0.48019377	10.74222534
SMART	SM00184:RING	8	3.686635945	0.01549805	TRIM5, TRIM30A, TRIM12A, TRIM30D, TRIM34A, TRIM25, RNF187, RNF225	117	234	10425	3.046241508	0.878594497	0.50483935	16.73295051
INTERPRO	IPR013320:Concanavalin A-like lectin/glucanase, subgroup	6	2.764976959	0.045422468	TRIM5, TRIM30A, TRIM30D, TRIM34A, LGALS7, TRIM25	190	211	20594	3.082165128	1	0.87758527	48.77062946
UP_SEQ_FEATURE	domain:B30.2/SPRY	3	1.382488479	0.118658304	TRIM30A, TRIM34A, TRIM25	182	59	18012	5.032222015	1	0.98998824	85.26846319
UP_SEQ_FEATURE	zinc finger region:RING-type	4	1.843317972	0.287602268	TRIM30A, TRIM12A, TRIM34A, TRIM25	182	186	18012	2.12832329	1	0.99989444	99.41534696
Annotation Cluster 3	Enrichment Score: 1.8479216774636271											
Category	Term	Count	%	PValue	Genes	List Total	Pop Hits	Pop Total	Fold Enrichment	Bonferroni	Benjamini	FDR
INTERPRO	IPR009030:Insulin-like growth factor binding protein, N-terminal	6	2.764976959	0.007020081	IGFBP6, FBN1, ESM1, PCSK5, CRIM1, VLDLR	190	130	20594	5.002591093	0.96984137	0.3935806	9.639479993
UP_SEQ_FEATURE	domain:IGFBP N-terminal	3	1.382488479	0.013823727	IGFBP6, ESM1, CRIM1	182	18	18012	16.49450549	0.999960838	0.96604096	19.02787083
INTERPRO	IPR000867:Insulin-like growth factor-binding protein, IGFBP	3	1.382488479	0.014280316	IGFBP6, ESM1, CRIM1	190	20	20594	16.25842105	0.999213943	0.54809154	18.69394267
GOTERM_BP_DIRECT	GO:0001558~regulation of cell growth	4	1.843317972	0.016474059	IGFBP6, RB1, ESM1, CRIM1	168	58	18082	7.422824302	0.999999984	0.98881685	23.28622914
SMART	SM00121:IB	3	1.382488479	0.018551597	IGFBP6, ESM1, CRIM1	117	19	10425	14.06882591	0.920180538	0.46847071	19.71089277
GOTERM_MF_DIRECT	GO:0005520~insulin-like growth factor binding	3	1.382488479	0.019301138	IGFBP6, ESM1, CRIM1	164	23	17446	13.87539767	0.999404213	0.56179558	23.65591337
Annotation Cluster 4	Enrichment Score: 1.6589524792215078											
Category	Term	Count	%	PValue	Genes	List Total	Pop Hits	Pop Total	Fold Enrichment	Bonferroni	Benjamini	FDR
UP_KEYWORDS	Carboxypeptidase	4	1.843317972	0.003398985	SCPEP1, CPE, CPQ, CPD	203	34	22680	13.14401623	0.538320277	0.10453486	4.262405228
GOTERM_MF_DIRECT	GO:0004180~carboxypeptidase activity	4	1.843317972	0.004208427	SCPEP1, CPE, CPQ, CPD	164	35	17446	12.15749129	0.7994705	0.33081751	5.673359925
GOTERM_MF_DIRECT	GO:0004185~serine-type carboxypeptidase activity	3	1.382488479	0.005384727	SCPEP1, CPE, CPD	164	12	17446	26.5945122	0.872179032	0.29025825	7.204861723
GOTERM_MF_DIRECT	GO:0016787~hydrolase activity	25	11.52073733	0.00840313	SCPEP1, NUDT18, PNLIPRP2, LIPA, ENPP1, CPQ, ATP10A, MMP19, SPAG1, ABHD3, NT5DC3, DTD2, PTPDC1, PPP1CB, ATP7A, MTM1, PSMC6, CPE, PRSS28, LTA4H, CPD, PRSS23, IDI1, PCSK5, USP45	164	1533	17446	1.734801839	0.959849491	0.36827612	11.02980552
UP_KEYWORDS	Hydrolase	25	11.52073733	0.011763518	SCPEP1, NUDT18, PNLIPRP2, LIPA, ENPP1, CPQ, ATP10A, MMP19, SPAG1, ABHD3, NT5DC3, DTD2, PTPDC1, PPP1CB, ATP7A, MTM1, CPE, PRSS28, LTA4H, CPD, PRSS23, CES2F, PCSK5, USP45, PLCXD2	203	1646	22680	1.696903675	0.931857146	0.20056156	14.04869202
GOTERM_BP_DIRECT	GO:0043171~peptide catabolic process	3	1.382488479	0.017351199	CPE, CPQ, LTA4H	168	22	18082	14.67694805	0.999999994	0.97735364	24.37078162
UP_KEYWORDS	Metalloprotease	5	2.304147465	0.039239151	CPE, CPQ, MMP19, LTA4H, CPD	203	143	22680	3.906438389	0.999886845	0.39638562	40.07775261
GOTERM_BP_DIRECT	GO:0006508~proteolysis	11	5.069124424	0.043966533	SCPEP1, CPE, CPQ, PRSS28, MMP19, LTA4H, CPD, PRSS23, PCSK5, GCA, USP45	168	582	18082	2.034261987	1	0.98799775	51.20423169
GOTERM_MF_DIRECT	GO:0008233~peptidase activity	10	4.608294931	0.053410957	SCPEP1, CPE, CPQ, PRSS28, MMP19, LTA4H, CPD, PRSS23, PCSK5, USP45	164	516	17446	2.061590093	0.999999999	0.7519716	53.24217484

UP_KEYWORDS	Protease	10	4.608294931	0.054307049	SCPEP1, CPE, CPQ, PRSS28, MMP19, LTA4H, CPD, PRSS23, PCSK5, USP45	203	542	22680	2.061330958	0.999996872	0.42367887	51.04933676
GOTERM_MF_DIRECT	GO:0008237~metallopeptidase activity	5	2.304147465	0.063383259	CPE, CPQ, MMP19, LTA4H, CPD	164	160	17446	3.324314024	1	0.78971024	59.62115492
UP_KEYWORDS	Zymogen	4	1.843317972	0.29534306	CPE, CPQ, MMP19, PCSK5	203	213	22680	2.098105877	1	0.77041504	98.86473583

Annotation Cluster 5	Enrichment Score: 1.4029285031607746											
Category	Term	Count	%	PValue	Genes	List Total	Pop Hits	Pop Total	Fold Enrichment	Bonferroni	Benjamini	FDR
GOTERM_CC_DIRECT	GO:0005576~extracellular region	31	14.28571429	6.69E-04	SCPEP1, ENPP1, LEPR, CPQ, LYPD8, FST, IGFBP6, ESM1, TIMP3, TBC1D15, C1QTNF3, DMKN, PCSK5, CRIM1, PNLIPRP1, PNLIPRP2, PRG4, FBN1, MMP19, NTN4, LGALS7, KITL, CBLN3, 1190002N15RIK, SDC1, CCDC3, CHRDL1, CPE, CLEC3B, PRSS28, PRSS23	182	1753	19662	1.910451784	0.147790226	0.05191159	0.85939647
UP_KEYWORDS	Secreted	29	13.3640553	0.001037542	SCPEP1, ENPP1, LEPR, CPQ, LYPD8, FST, IGFBP6, ESM1, TIMP3, C1QTNF3, DMKN, PCSK5, PNLIPRP1, PNLIPRP2, PRG4, FBN1, MMP19, LGALS7, NTN4, KITL, CBLN3, 1190002N15RIK, SDC1, CCDC3, CHRDL1, CPE, CLEC3B, PRSS28, PRSS23	203	1685	22680	1.922848665	0.20993843	0.04603556	1.319293255
GOTERM_CC_DIRECT	GO:0005615~extracellular space	24	11.05990783	0.011219865	PNLIPRP1, PNLIPRP2, ENPP1, PRG4, LEPR, LYPD8, CPQ, IGFBP6, FBN1, LGALS7, TIMP3, KITL, CBLN3, 1190002N15RIK, C1QTNF3, CPE, CLEC3B, DMKN, PRSS28, ANXA13, CPD, CES2F, PCSK5, VLDLR	182	1504	19662	1.723930325	0.93257264	0.41686752	13.54469105
UP_KEYWORDS	Glycoprotein	47	21.65898618	0.017365577	SCPEP1, TM7SF3, ENPP1, CPQ, LYPD8, LEPR, FST, IGFBP6, ESM1, SDC2, SLC11A2, EDNRB, STT3A, DMKN, MFAP3L, PCSK5, CRIM1, PHYHIP, PNLIPRP2, LIPA, TRPC6, LRRN4, PRG4, AF529169, FBN1, MMP19, NTN4, TSPAN13, TPBG, KITL, ST6GALNAC2, ST6GALNAC1, ATP7A, CBLN3, MGAT2, SDC1, CCDC3, CHRDL1, CPE, PLXDC1, PRSS28, DSC1, CPD, PRSS23, CLOCK, GFRA2, VLDLR	203	3815	22680	1.376417951	0.981250999	0.26353491	20.07798758
UP_SEQ_FEATURE	signal peptide	41	18.89400922	0.058064893	SCPEP1, TM7SF3, CPQ, LYPD8, LEPR, FST, IGFBP6, ESM1, TIMP3, SDC2, EDNRB, C1QTNF3, DMKN, MFAP3L, DNAJC3, PCSK5, CRIM1, PNLIPRP1, PNLIPRP2, LIPA, LRRN4, PRG4, FBN1, MMP19, NTN4, TPBG, KITL, CBLN3, 1190002N15RIK, SDC1, CCDC3, CHRDL1, CPE, CLEC3B, PLXDC1, PRSS28, DSC1, CPD, PRSS23, GFRA2, VLDLR	182	3124	18012	1.298863109	1	0.99213498	59.62691761
UP_SEQ_FEATURE	glycosylation site:N-linked (GlcNAc...)	41	18.89400922	0.241125708	SCPEP1, TM7SF3, ENPP1, CPQ, LYPD8, LEPR, FST, SLC11A2, EDNRB, STT3A, MFAP3L, PCSK5, CRIM1, PHYHIP, PNLIPRP2, LIPA, TRPC6, LRRN4, PRG4, AF529169, FBN1, MMP19, NTN4, TSPAN13, TPBG, KITL, ST6GALNAC2, ST6GALNAC1, ATP7A, CBLN3, MGAT2, SDC1, CHRDL1, CPE, PLXDC1, PRSS28, DSC1, CPD, PRSS23, GFRA2, VLDLR	182	3563	18012	1.138829175	1	0.99956334	98.47570363
UP_KEYWORDS	Signal	45	20.73732719	0.291639002	SCPEP1, TM7SF3, CPQ, LYPD8, LEPR, FST, IGFBP6, DPY19L4, ESM1, TIMP3, SDC2, EDNRB, C1QTNF3, DMKN, MFAP3L, DNAJC3, PCSK5, CRIM1, PNLIPRP1, PNLIPRP2, LIPA, LRRN4, PRG4, FBN1, MMP19, NTN4, TSPAN13, RB1, TPBG, KITL, CBLN3, 1190002N15RIK, SDC1, CCDC3, CHRDL1, CPE, CLEC3B, PLXDC1, PRSS28, DSC1, CPD, PRSS23, CES2F, GFRA2, VLDLR	203	4543	22680	1.106666565	1	0.77163066	98.78597777

UP_KEYWORDS	Disulfide bond	28	12.90322581	0.598173225	ENPP1, LEPR, FST, IGFBP6, GJA1, TIMP3, EDNRB, MFAP3L, DNAJC3, SOAT1, PNLIPRP1, PNLIPRP2, PRG4, MMP19, FBN1, NTN4, KITL, TPBG, ST6GALNAC2, ST6GALNAC1, CBLN3, CLEC3B, PRKAR1B, PRSS28, IKBKG, CLDN1, PRSS23, VLDLR	203	3124	22680	1.001368714	1	0.91239057	99.99914048
UP_SEQ_FEATURE	disulfide bond	24	11.05990783	0.715755169	PNLIPRP1, PNLIPRP2, ENPP1, PRG4, LEPR, FST, IGFBP6, FBN1, MMP19, NTN4, GJA1, TIMP3, KITL, ST6GALNAC2, ST6GALNAC1, CBLN3, EDNRB, CLEC3B, PRKAR1B, PRSS28, IKBKG, MFAP3L, PRSS23, VLDLR	182	2510	18012	0.946298323	1	1	99.99999948
Annotation Cluster 6	Enrichment Score: 1.3282671860094175												
Category	Term	Count	%	PValue	Genes	List Total	Pop Hits	Pop Total	Fold Enrichment	Bonferroni	Benjamini	FDR
KEGG_PATHWAY	mmu04670:Leukocyte transendothelial migration	6	2.764976959	0.004820885	PRKCA, CDC42, GNAI3, VAV3, ROCK1, CLDN1	73	118	7691	5.357093104	0.551659431	0.33041762	5.70284416
INTERPRO	IPR002219:Protein kinase C-like, phorbol ester/diacylglycerol binding	4	1.843317972	0.022818808	PRKCA, PDZD8, VAV3, ROCK1	190	66	20594	6.569059011	0.999989586	0.68248632	28.2603212
SMART	SM00109:C1	4	1.843317972	0.034247223	PRKCA, PDZD8, VAV3, ROCK1	117	64	10425	5.568910256	0.990944648	0.60971571	33.53806064
KEGG_PATHWAY	mmu04666:Fc gamma R-mediated phagocytosis	4	1.843317972	0.04374038	PRKCA, CDC42, VAV3, MARCKS	73	84	7691	5.016960209	0.999403534	0.84372252	41.9260904
KEGG_PATHWAY	mmu04510:Focal adhesion	5	2.304147465	0.128385849	PRKCA, CDC42, VAV3, ROCK1, PPP1CB	73	207	7691	2.544834888	1	0.8270236	81.16812875
GOTERM_BP_DIRECT	GO:0035556~intracellular signal transduction	5	2.304147465	0.506934777	PRKCA, PDZD8, VAV3, ROCK1, LATS2	168	400	18082	1.345386905	1	0.9999971	99.99874325
Annotation Cluster 7	Enrichment Score: 1.2935650383316635												
Category	Term	Count	%	PValue	Genes	List Total	Pop Hits	Pop Total	Fold Enrichment	Bonferroni	Benjamini	FDR
GOTERM_MF_DIRECT	GO:0001047~core promoter binding	5	2.304147465	0.0053359	NPAS2, RB1, CRY1, CLOCK, MED1	164	75	17446	7.091869919	0.869765824	0.3348111	7.141751609
GOTERM_BP_DIRECT	GO:0032922~circadian regulation of gene expression	4	1.843317972	0.018834995	NPAS2, CRY1, PPP1CB, CLOCK	168	61	18082	7.057767369	0.999999999	0.96757975	26.17278851
KEGG_PATHWAY	mmu04710:Circadian rhythm	3	1.382488479	0.033743585	NPAS2, CRY1, CLOCK	73	31	7691	10.19575784	0.996647747	0.85033775	34.10371326
GOTERM_BP_DIRECT	GO:0042752~regulation of circadian rhythm	3	1.382488479	0.083328506	NPAS2, CRY1, PPP1CB	168	52	18082	6.209478022	1	0.99464666	75.05450449
UP_KEYWORDS	Biological rhythms	4	1.843317972	0.102784368	NPAS2, CRY1, PPP1CB, CLOCK	203	126	22680	3.54679803	1	0.49535223	75.03200349
GOTERM_BP_DIRECT	GO:0048511~rhythmic process	4	1.843317972	0.115221905	NPAS2, CRY1, PPP1CB, CLOCK	168	128	18082	3.363467262	1	0.9968457	85.82371816
GOTERM_BP_DIRECT	GO:0007623~circadian rhythm	3	1.382488479	0.2632841	NPAS2, CRY1, CLOCK	168	108	18082	2.989748677	1	0.99945451	99.23735376
Annotation Cluster 8	Enrichment Score: 1.2888975220763432												
Category	Term	Count	%	PValue	Genes	List Total	Pop Hits	Pop Total	Fold Enrichment	Bonferroni	Benjamini	FDR
UP_KEYWORDS	Lipoprotein	14	6.451612903	0.022893316	RAB2A, GNAI3, SGMS2, RP2, CKAP4, LYPD8, MMP19, PGR, EDNRB, CDC42, ANXA13, MARCKS, CPD, GFRA2	203	780	22680	2.00530504	0.994790087	0.31306371	25.64291338
UP_KEYWORDS	Palmitate	7	3.225806452	0.055649503	PGR, EDNRB, GNAI3, SGMS2, RP2, CKAP4, CPD	203	304	22680	2.572595281	0.999997734	0.41816259	51.93092669
UP_SEQ_FEATURE	lipid moiety-binding region:S-palmitoyl cysteine	5	2.304147465	0.106693374	EDNRB, GNAI3, SGMS2, RP2, CPD	182	179	18012	2.764442262	1	0.99207884	81.92644646
Annotation Cluster 9	Enrichment Score: 1.2148484567512345												
Category	Term	Count	%	PValue	Genes	List Total	Pop Hits	Pop Total	Fold Enrichment	Bonferroni	Benjamini	FDR

GOTERM_BP_DIRECT	GO:0018279~protein N-linked glycosylation via asparagine	4	1.843317972	0.002876258	MGAT2, STT3A, ST6GALNAC2, ST6GALNAC1	168	31	18082	13.88786482	0.955692235	0.95569223	4.492610681
GOTERM_MF_DIRECT	GO:0016757~transferase activity, transferring glycosyl groups	6	2.764976959	0.045841357	MGAT2, STT3A, DPY19L4, ST6GALNAC2, ST6GALNAC1, TNKS2	164	208	17446	3.068597561	0.999999983	0.74722945	47.78918687
UP_KEYWORDS	Glycosyltransferase	6	2.764976959	0.051299099	MGAT2, STT3A, DPY19L4, ST6GALNAC2, ST6GALNAC1, TNKS2	203	225	22680	2.979310345	0.999993568	0.43404908	49.01963565
GOTERM_BP_DIRECT	GO:0006486~protein glycosylation	3	1.382488479	0.290656699	STT3A, ST6GALNAC2, ST6GALNAC1	168	116	18082	2.783559113	1	0.99968963	99.5832167
UP_SEQ_FEATURE	topological domain:Lumenal	6	2.764976959	0.428728492	MGAT2, MTDH, STT3A, DERL1, ST6GALNAC2, ST6GALNAC1	182	427	18012	1.390637466	1	0.99998809	99.97943571
Annotation Cluster 10	Enrichment Score: 1.195825607333584											
Category	Term	Count	%	PValue	Genes	List Total	Pop Hits	Pop Total	Fold Enrichment	Bonferroni	Benjamini	FDR
GOTERM_CC_DIRECT	GO:0005783~endoplasmic reticulum	21	9.677419355	0.019763125	PRKCA, SOAT1, RAB2A, DERL1, MTDH, SLC16A11, ORMDL1, CKAP4, CPQ, ATP10A, GJA1, REEP3, TPBG, PGR, CBLN3, CCDC3, STT3A, PUM3, YIPF4, DNAJC3, ACSL4	182	1323	19662	1.714809001	0.991525431	0.54847036	22.70001504
UP_KEYWORDS	Endoplasmic reticulum	15	6.912442396	0.062708418	SOAT1, RAB2A, DERL1, MTDH, SLC16A11, CKAP4, CPQ, ORMDL1, ATP10A, GJA1, REEP3, CBLN3, STT3A, ACSL4, DNAJC3	203	997	22680	1.680904783	0.999999587	0.40845974	56.33051818
GOTERM_CC_DIRECT	GO:0005789~endoplasmic reticulum membrane	10	4.608294931	0.208613167	RAB2A, SOAT1, MTDH, DERL1, SLC16A11, ORMDL1, CKAP4, ATP10A, ACSL4, REEP3	182	710	19662	1.521591085	1	0.86426995	95.10985424
Annotation Cluster 11	Enrichment Score: 1.0516202570899469											
Category	Term	Count	%	PValue	Genes	List Total	Pop Hits	Pop Total	Fold Enrichment	Bonferroni	Benjamini	FDR
UP_SEQ_FEATURE	zinc finger region:PHD-type 1	3	1.382488479	0.03199127	G2E3, WHSC1, KDM5D	182	28	18012	10.60361068	1	0.96616024	38.92073406
INTERPRO	IPR001965:Zinc finger, PHD-type	4	1.843317972	0.051403675	G2E3, WHSC1, PHF6, KDM5D	190	91	20594	4.76437247	1	0.88759398	53.20036549
SMART	SM00249:PHD	4	1.843317972	0.080779817	G2E3, WHSC1, PHF6, KDM5D	117	91	10425	3.916596224	0.999988475	0.8497066	62.74869196
INTERPRO	IPR019786:Zinc finger, PHD-type, conserved site	3	1.382488479	0.116914357	G2E3, WHSC1, KDM5D	190	64	20594	5.080756579	1	0.97897613	83.28616071
INTERPRO	IPR011011:Zinc finger, FYVE/PHD-type	3	1.382488479	0.35538767	G2E3, WHSC1, KDM5D	190	136	20594	2.390944272	1	0.99996933	99.81963821
Annotation Cluster 12	Enrichment Score: 1.045139120825286											
Category	Term	Count	%	PValue	Genes	List Total	Pop Hits	Pop Total	Fold Enrichment	Bonferroni	Benjamini	FDR
KEGG_PATHWAY	mmu05205:Proteoglycans in cancer	8	3.686635945	0.00277497	PRKCA, CDC42, SDC1, ROCK1, ANK3, TIMP3, PPP1CB, SDC2	73	203	7691	4.151967069	0.369527291	0.36952729	3.320124668
KEGG_PATHWAY	mmu04670:Leukocyte transendothelial migration	6	2.764976959	0.004820885	PRKCA, CDC42, GNAI3, VAV3, ROCK1, CLDN1	73	118	7691	5.357093104	0.551659431	0.33041762	5.70284416
KEGG_PATHWAY	mmu04022:cGMP-PKG signaling pathway	5	2.304147465	0.066006206	EDNRB, GNAI3, TRPC6, ROCK1, PPP1CB	73	163	7691	3.231784184	0.999988057	0.80197095	56.38293817
KEGG_PATHWAY	mmu04071:Sphingolipid signaling pathway	4	1.843317972	0.109831813	PRKCA, GNAI3, SGMS2, ROCK1	73	124	7691	3.398585948	0.999999996	0.88303918	75.67536543
KEGG_PATHWAY	mmu04062:Chemokine signaling pathway	5	2.304147465	0.110963558	CDC42, GNAI3, VAV3, ROCK1, IKBKG	73	196	7691	2.687657255	0.999999997	0.85807309	76.04848729
KEGG_PATHWAY	mmu04360:Axon guidance	4	1.843317972	0.119869672	CDC42, GNAI3, ROCK1, NTN4	73	129	7691	3.26685781	0.999999999	0.85439925	78.80649746
KEGG_PATHWAY	mmu04510:Focal adhesion	5	2.304147465	0.128385849	PRKCA, CDC42, VAV3, ROCK1, PPP1CB	73	207	7691	2.544834888	1	0.8270236	81.16812875

KEGG_PATHWAY	mmu04921:Oxytocin signaling pathway	4	1.843317972	0.165383194	PRKCA, GNAI3, ROCK1, PPP1CB	73	150	7691	2.809497717	1	0.84673996	88.88249119
KEGG_PATHWAY	mmu04024:cAMP signaling pathway	4	1.843317972	0.280452547	GNAI3, VAV3, ROCK1, PPP1CB	73	197	7691	2.13921146	1	0.9258538	98.1670063
KEGG_PATHWAY	mmu04810:Regulation of actin cytoskeleton	4	1.843317972	0.321618477	CDC42, VAV3, ROCK1, PPP1CB	73	213	7691	1.978519519	1	0.93170925	99.10406172
KEGG_PATHWAY	mmu04270:Vascular smooth muscle contraction	3	1.382488479	0.334224629	PRKCA, ROCK1, PPP1CB	73	127	7691	2.488728293	1	0.92552415	99.28666359
KEGG_PATHWAY	mmu04611:Platelet activation	3	1.382488479	0.347932855	GNAI3, ROCK1, PPP1CB	73	131	7691	2.412736589	1	0.92784912	99.44600331
Annotation Cluster 13	Enrichment Score: 0.9637849446661065											
Category	Term	Count	%	PValue	Genes	List Total	Pop Hits	Pop Total	Fold Enrichment	Bonferroni	Benjamini	FDR
UP_KEYWORDS	Cholesterol metabolism	3	1.382488479	0.086265048	SOAT1, IDI1, VLDLR	203	55	22680	6.094043887	0.999999999	0.4726869	68.46795662
UP_KEYWORDS	Sterol metabolism	3	1.382488479	0.105587063	SOAT1, IDI1, VLDLR	203	62	22680	5.406006674	1	0.48654339	76.01165145
UP_KEYWORDS	Steroid metabolism	3	1.382488479	0.140993662	SOAT1, IDI1, VLDLR	203	74	22680	4.529356943	1	0.57788636	85.69197002
Annotation Cluster 14	Enrichment Score: 0.9324776259452378											
Category	Term	Count	%	PValue	Genes	List Total	Pop Hits	Pop Total	Fold Enrichment	Bonferroni	Benjamini	FDR
KEGG_PATHWAY	mmu04916:Melanogenesis	4	1.843317972	0.065290714	PRKCA, EDNRB, GNAI3, KITL	73	99	7691	4.256814723	0.999986438	0.84557483	55.97520377
KEGG_PATHWAY	mmu05200:Pathways in cancer	8	3.686635945	0.075318647	PRKCA, EDNRB, CDC42, GNAI3, ROCK1, IKBKG, RB1, KITL	73	395	7691	2.133795734	0.999997737	0.80305903	61.38308727
KEGG_PATHWAY	mmu04015:Rap1 signaling pathway	4	1.843317972	0.324200138	PRKCA, CDC42, GNAI3, KITL	73	214	7691	1.969274101	1	0.92587031	99.14462318
Annotation Cluster 15	Enrichment Score: 0.9165527789762681											
Category	Term	Count	%	PValue	Genes	List Total	Pop Hits	Pop Total	Fold Enrichment	Bonferroni	Benjamini	FDR
UP_KEYWORDS	GTP-binding	8	3.686635945	0.029593041	RAB2A, CDC42, GTPBP4, GNAI3, RP2, HBS1L, TUBB5, SPAG1	203	332	22680	2.692147902	0.998907212	0.34700706	31.90829417
GOTERM_MF_DIRECT	GO:0003924~GTPase activity	6	2.764976959	0.046603399	RAB2A, CDC42, GTPBP4, GNAI3, HBS1L, TUBB5	164	209	17446	3.053915276	0.999999987	0.72713627	48.36372393
GOTERM_MF_DIRECT	GO:0005525~GTP binding	8	3.686635945	0.068394645	RAB2A, CDC42, GTPBP4, GNAI3, RP2, HBS1L, TUBB5, SPAG1	164	383	17446	2.221995797	1	0.79562193	62.51254308
UP_SEQ_FEATURE	nucleotide phosphate-binding region:GTP	7	3.225806452	0.102649762	RAB2A, CDC42, GTPBP4, GNAI3, HBS1L, TUBB5, SPAG1	182	319	18012	2.171690379	1	0.99644644	80.64541871
INTERPRO	IPR005225:Small GTP-binding protein domain	3	1.382488479	0.445826564	RAB2A, CDC42, GTPBP4	190	165	20594	1.970717703	1	0.99999199	99.97951125
INTERPRO	IPR027417:P-loop containing nucleoside triphosphate hydrolase	8	3.686635945	0.733719638	RAB2A, CDC42, GTPBP4, PSMC6, GNAI3, HBS1L, MPP6, MYH9	190	909	20594	0.953922761	1	1	99.99999946
Annotation Cluster 16	Enrichment Score: 0.8582635651499091											
Category	Term	Count	%	PValue	Genes	List Total	Pop Hits	Pop Total	Fold Enrichment	Bonferroni	Benjamini	FDR

UP_KEYWORDS	Nucleus	51	23.50230415	0.056267572	POU6F1, TMEM18, MTDH, RNF187, SF3B5, LATS2, PGR, KDM1B, TSPYL1, KLHL7, TRIM30A, GTF2E1, G2E3, NPAS2, CDYL, ACTR6, WDR77, PUM3, HBP1, HIST3H2A, MFAP3L, DYRK2, CRY1, FAM83G, KDM5D, MAF, PRKCA, GTPBP4, RCOR1, ZFX, LGALS7, RAI14, WHSC1, RB1, GRHL2, PPP1CB, PWP1, QK, CTR9, TNKS2, PSMC6, SRSF7, IKBKG, ZFP800, PIAS2, PLCXD2, CLOCK, MED1, THOC1, FBXO11, PHF6	203	4534	22680	1.256711741	0.999998047	0.40894511	52.331872
UP_KEYWORDS	Transcription	24	11.05990783	0.067809295	MAF, POU6F1, TMEM18, RCOR1, ZFX, WHSC1, RB1, GRHL2, CTR9, KDM1B, PGR, GTF2E1, TRIM30A, NPAS2, CDYL, IKBKG, ZFP800, PIAS2, HBP1, CRY1, CLOCK, THOC1, PHF6, MED1	203	1859	22680	1.442377251	0.99999988	0.41216865	59.27528921
UP_KEYWORDS	Transcription regulation	23	10.59907834	0.080504316	MAF, POU6F1, RCOR1, ZFX, WHSC1, RB1, GRHL2, CTR9, KDM1B, PGR, GTF2E1, TRIM30A, NPAS2, CDYL, IKBKG, ZFP800, PIAS2, HBP1, CRY1, CLOCK, THOC1, PHF6, MED1	203	1799	22680	1.428379751	0.999999995	0.45913398	65.82791059
GOTERM_BP_DIRECT	GO:0006351~transcription, DNA-templated	24	11.05990783	0.100770504	MAF, POU6F1, TMEM18, RCOR1, ZFX, WHSC1, RB1, GRHL2, CTR9, KDM1B, PGR, GTF2E1, TRIM30A, NPAS2, CDYL, IKBKG, ZFP800, PIAS2, HBP1, CRY1, CLOCK, THOC1, PHF6, MED1	168	1885	18082	1.370367563	1	0.99580014	81.64102511
UP_KEYWORDS	DNA-binding	19	8.755760369	0.185221354	MAF, POU6F1, TMEM18, ZFX, WHSC1, RB1, GRHL2, PGR, TRIM30A, NPAS2, ZFP800, PIAS2, HIST3H2A, HBP1, PUM3, CLOCK, THOC1, PHF6, MED1	203	1604	22680	1.323415599	1	0.66948557	92.72417936
GOTERM_MF_DIRECT	GO:0003677~DNA binding	22	10.13824885	0.200325293	MAF, POU6F1, TMEM18, RCOR1, ZFX, WHSC1, RB1, GRHL2, KDM1B, PGR, TRIM30A, NPAS2, ZFP800, PIAS2, PUM3, HIST3H2A, HBP1, CLOCK, THOC1, PHF6, KDM5D, MED1	164	1847	17446	1.267090998	1	0.95225496	95.47703793
GOTERM_BP_DIRECT	GO:0006355~regulation of transcription, DNA-templated	26	11.98156682	0.206433734	2610044O15RIK8, POU6F1, ZFP455, KDM1B, PGR, GTF2E1, TRIM30A, NPAS2, CDYL, HBP1, CRY1, MAF, RCOR1, ZFX, WHSC1, RB1, GRHL2, CTR9, ZFP944, IKBKG, ZFP800, PIAS2, CLOCK, THOC1, MED1, PHF6	168	2279	18082	1.227909066	1	0.99921352	97.50247345
GOTERM_BP_DIRECT	GO:0045944~positive regulation of transcription from RNA polymerase II promoter	10	4.608294931	0.574095271	MAF, PGR, NPAS2, IKBKG, PIAS2, RB1, GRHL2, CLOCK, CTR9, MED1	168	995	18082	1.081718114	1	0.99999946	99.99987854
Annotation Cluster 17	Enrichment Score: 0.8555163128961274											
Category	Term	Count	%	PValue	Genes	List Total	Pop Hits	Pop Total	Fold Enrichment	Bonferroni	Benjamini	FDR
INTERPRO	IPR009030:Insulin-like growth factor binding protein, N-terminal	6	2.764976959	0.007020081	IGFBP6, FBN1, ESM1, PCSK5, CRIM1, VLDLR	190	130	20594	5.002591093	0.96984137	0.3935806	9.639479993
SMART	SM00181:EGF	3	1.382488479	0.601669974	FBN1, PCSK5, VLDLR	117	181	10425	1.476838079	1	0.99998759	99.99794237
INTERPRO	IPR000742:Epidermal growth factor-like domain	3	1.382488479	0.642318779	FBN1, PCSK5, VLDLR	190	237	20594	1.372018654	1	0.99999993	99.99996236
Annotation Cluster 18	Enrichment Score: 0.8324974007035632											
Category	Term	Count	%	PValue	Genes	List Total	Pop Hits	Pop Total	Fold Enrichment	Bonferroni	Benjamini	FDR
GOTERM_MF_DIRECT	GO:0001047~core promoter binding	5	2.304147465	0.0053359	NPAS2, RB1, CRY1, CLOCK, MED1	164	75	17446	7.091869919	0.869765824	0.3348111	7.141751609
GOTERM_MF_DIRECT	GO:0008134~transcription factor binding	6	2.764976959	0.216433854	RCOR1, PIAS2, RB1, CRY1, CLOCK, MED1	164	342	17446	1.866281558	1	0.95941427	96.58785488

GOTERM_CC_DIRECT	GO:0005667~transcription factor complex	4	1.843317972	0.446832833	NPAS2, RCOR1, RB1, CLOCK	182	267	19662	1.618471416	1	0.94099979	99.95179237
GOTERM_BP_DIRECT	GO:0045892~negative regulation of transcription, DNA-templated	4	1.843317972	0.906426217	RCOR1, RB1, CRY1, CLOCK	168	579	18082	0.743564438	1	1	100
Annotation Cluster 19	Enrichment Score: 0.8281228451964939											
Category	Term	Count	%	PValue	Genes	List Total	Pop Hits	Pop Total	Fold Enrichment	Bonferroni	Benjamini	FDR
INTERPRO	IPR002110:Ankyrin repeat	6	2.764976959	0.063815712	ANKRD26, TRPC6, ANK3, RAI14, ANKRD10, TNKS2	190	232	20594	2.803176044	1	0.91962476	61.27954155
UP_KEYWORDS	ANK repeat	6	2.764976959	0.064295867	ANKRD26, TRPC6, ANK3, RAI14, ANKRD10, TNKS2	203	240	22680	2.793103448	0.999999719	0.40559221	57.26734923
INTERPRO	IPR020683:Ankyrin repeat-containing domain	6	2.764976959	0.072712043	ANKRD26, TRPC6, ANK3, RAI14, ANKRD10, TNKS2	190	242	20594	2.687342323	1	0.93143239	66.24979563
SMART	SM00248:ANK	6	2.764976959	0.105971734	ANKRD26, TRPC6, ANK3, RAI14, ANKRD10, TNKS2	117	225	10425	2.376068376	0.999999729	0.84897378	73.10594481
UP_SEQ_FEATURE	repeat:ANK 3	4	1.843317972	0.218235339	ANKRD26, TRPC6, RAI14, ANKRD10	182	160	18012	2.474175824	1	0.99923775	97.60800534
UP_SEQ_FEATURE	repeat:ANK 2	4	1.843317972	0.306630685	ANKRD26, TRPC6, RAI14, ANKRD10	182	193	18012	2.051130217	1	0.99992766	99.6121906
UP_SEQ_FEATURE	repeat:ANK 1	4	1.843317972	0.306630685	ANKRD26, TRPC6, RAI14, ANKRD10	182	193	18012	2.051130217	1	0.99992766	99.6121906
UP_SEQ_FEATURE	repeat:ANK 4	3	1.382488479	0.36555596	ANKRD26, RAI14, ANKRD10	182	127	18012	2.337803928	1	0.9999685	99.89912601
Annotation Cluster 20	Enrichment Score: 0.8011403629390492											
Category	Term	Count	%	PValue	Genes	List Total	Pop Hits	Pop Total	Fold Enrichment	Bonferroni	Benjamini	FDR
UP_KEYWORDS	Tight junction	4	1.843317972	0.03805189	USP53, MTDH, PLXDC1, CLDN1	203	83	22680	5.384295804	0.99985023	0.40430368	39.12346936
GOTERM_CC_DIRECT	GO:0016328~lateral plasma membrane	3	1.382488479	0.088404358	ANK3, CLDN1, GJA1	182	54	19662	6.001831502	1	0.81761898	69.69663598
GOTERM_CC_DIRECT	GO:0005923~bicellular tight junction	4	1.843317972	0.120412194	MTDH, ANK3, PLXDC1, CLDN1	182	131	19662	3.298716551	1	0.81796638	80.89003804
UP_KEYWORDS	Cell junction	8	3.686635945	0.373714862	CBLN3, USP53, MTDH, ANK3, PLXDC1, CLDN1, GJA1, DSC1	203	661	22680	1.352183212	1	0.80489623	99.74881388
GOTERM_CC_DIRECT	GO:0030054~cell junction	7	3.225806452	0.651984762	CBLN3, MTDH, ANK3, PLXDC1, CLDN1, GJA1, DSC1	182	718	19662	1.053246197	1	0.98708576	99.99987779
Annotation Cluster 21	Enrichment Score: 0.73337270972125											
Category	Term	Count	%	PValue	Genes	List Total	Pop Hits	Pop Total	Fold Enrichment	Bonferroni	Benjamini	FDR
UP_SEQ_FEATURE	active site:Charge relay system	7	3.225806452	0.017629967	PNLIPRP1, PNLIPRP2, LIPA, PRSS28, ABHD3, PRSS23, PCSK5	182	205	18012	3.379362101	0.999997664	0.92523292	23.63912094
GOTERM_MF_DIRECT	GO:0004252~serine-type endopeptidase activity	4	1.843317972	0.319303916	PRSS28, MMP19, PRSS23, PCSK5	164	212	17446	2.007132996	1	0.98480933	99.51412115
GOTERM_MF_DIRECT	GO:0008236~serine-type peptidase activity	3	1.382488479	0.452903564	PRSS28, PRSS23, PCSK5	164	163	17446	1.957878198	1	0.99631947	99.97642881
UP_KEYWORDS	Serine protease	3	1.382488479	0.45713837	PRSS28, PRSS23, PCSK5	203	173	22680	1.937412796	1	0.84648846	99.95966011
Annotation Cluster 22	Enrichment Score: 0.6937209744032324											
Category	Term	Count	%	PValue	Genes	List Total	Pop Hits	Pop Total	Fold Enrichment	Bonferroni	Benjamini	FDR
KEGG_PATHWAY	mmu04728:Dopaminergic synapse	4	1.843317972	0.130237103	PRKCA, GNAI3, PPP1CB, CLOCK	73	134	7691	3.144960131	1	0.80880896	81.64841808
KEGG_PATHWAY	mmu04921:Oxytocin signaling pathway	4	1.843317972	0.165383194	PRKCA, GNAI3, ROCK1, PPP1CB	73	150	7691	2.809497717	1	0.84673996	88.88249119
KEGG_PATHWAY	mmu04261:Adrenergic signaling in cardiomyocytes	3	1.382488479	0.385133215	PRKCA, GNAI3, PPP1CB	73	142	7691	2.225834459	1	0.9197765	99.72865289

Annotation Cluster 23	Enrichment Score: 0.6235517016258065											
Category	Term	Count	%	PValue	Genes	List Total	Pop Hits	Pop Total	Fold Enrichment	Bonferroni	Benjamini	FDR
UP_SEQ_FEATURE	repeat:TPR 8	3	1.382488479	0.058398261	SPAG1, DNAJC3, CTR9	182	39	18012	7.61284869	1	0.98755707	59.84302903
UP_SEQ_FEATURE	repeat:TPR 7	3	1.382488479	0.093036601	SPAG1, DNAJC3, CTR9	182	51	18012	5.821590175	1	0.99845347	77.25150337
UP_SEQ_FEATURE	repeat:TPR 6	3	1.382488479	0.112095981	SPAG1, DNAJC3, CTR9	182	57	18012	5.208791209	1	0.99189368	83.51468272
UP_SEQ_FEATURE	repeat:TPR 5	3	1.382488479	0.135459555	SPAG1, DNAJC3, CTR9	182	64	18012	4.63907967	1	0.99195927	88.99717289
UP_SEQ_FEATURE	repeat:TPR 4	3	1.382488479	0.214065567	SPAG1, DNAJC3, CTR9	182	86	18012	3.452338359	1	0.99933571	97.40707497
INTERPRO	IPR013026:Tetratricopeptide repeat-containing domain	3	1.382488479	0.30171086	SPAG1, DNAJC3, CTR9	190	120	20594	2.709736842	1	0.99995061	99.42990942
INTERPRO	IPR019734:Tetratricopeptide repeat	3	1.382488479	0.321969477	SPAG1, DNAJC3, CTR9	190	126	20594	2.580701754	1	0.99993595	99.62677103
UP_SEQ_FEATURE	repeat:TPR 3	3	1.382488479	0.358318777	SPAG1, DNAJC3, CTR9	182	125	18012	2.375208791	1	0.99997056	99.88019635
UP_KEYWORDS	TPR repeat	3	1.382488479	0.367621315	SPAG1, DNAJC3, CTR9	203	144	22680	2.327586207	1	0.80317009	99.71568879
SMART	SM00028:TPR	3	1.382488479	0.382973042	SPAG1, DNAJC3, CTR9	117	119	10425	2.246283129	1	0.99928458	99.6519937
UP_SEQ_FEATURE	repeat:TPR 2	3	1.382488479	0.40124354	SPAG1, DNAJC3, CTR9	182	137	18012	2.167161306	1	0.99998325	99.958068
UP_SEQ_FEATURE	repeat:TPR 1	3	1.382488479	0.40124354	SPAG1, DNAJC3, CTR9	182	137	18012	2.167161306	1	0.99998325	99.958068
INTERPRO	IPR011990:Tetratricopeptide-like helical	3	1.382488479	0.562040256	SPAG1, DNAJC3, CTR9	190	204	20594	1.593962848	1	0.99999957	99.99930678

Annotation Cluster 24	Enrichment Score: 0.6139769913390101											
Category	Term	Count	%	PValue	Genes	List Total	Pop Hits	Pop Total	Fold Enrichment	Bonferroni	Benjamini	FDR
GOTERM_CC_DIRECT	GO:0031012~extracellular matrix	6	2.764976959	0.134779206	CKAP4, FBN1, MMP19, TUBB5, MYH9, TIMP3	182	294	19662	2.204754429	1	0.80749377	84.54734042
GOTERM_CC_DIRECT	GO:0005604~basement membrane	3	1.382488479	0.224644759	FBN1, NTN4, TIMP3	182	97	19662	3.341225785	1	0.85936732	96.24444027
GOTERM_CC_DIRECT	GO:0005578~proteinaceous extracellular matrix	5	2.304147465	0.331967484	CLEC3B, FBN1, MMP19, NTN4, TIMP3	182	316	19662	1.70938239	1	0.92616076	99.45036817
UP_KEYWORDS	Extracellular matrix	4	1.843317972	0.348238917	FBN1, MMP19, NTN4, TIMP3	203	235	22680	1.901687454	1	0.80201397	99.58165683

Annotation Cluster 25	Enrichment Score: 0.5561598616100307											
Category	Term	Count	%	PValue	Genes	List Total	Pop Hits	Pop Total	Fold Enrichment	Bonferroni	Benjamini	FDR
GOTERM_CC_DIRECT	GO:0005769~early endosome	5	2.304147465	0.15586583	SLC11A2, DERL1, GJA1, A230046K03RIK, STEAP2	182	226	19662	2.39010989	1	0.82808281	88.75951923
UP_KEYWORDS	Endosome	6	2.764976959	0.357152128	SLC11A2, MTM1, SLC48A1, RASSF9, A230046K03RIK, STEAP2	203	443	22680	1.513193742	1	0.80165172	99.64922829
GOTERM_CC_DIRECT	GO:0005768~endosome	7	3.225806452	0.385404035	SLC11A2, MTM1, SLC48A1, RASSF9, GJA1, A230046K03RIK, STEAP2	182	544	19662	1.39013009	1	0.92463383	99.81248856

Annotation Cluster 26	Enrichment Score: 0.5195608585659518											
Category	Term	Count	%	PValue	Genes	List Total	Pop Hits	Pop Total	Fold Enrichment	Bonferroni	Benjamini	FDR
GOTERM_MF_DIRECT	GO:0004842~ubiquitin-protein transferase activity	6	2.764976959	0.190168276	KLHL7, TRIM5, G2E3, TRIM25, RNF187, FBXO11	164	326	17446	1.957878198	1	0.95982679	94.61312905
UP_KEYWORDS	Ubl conjugation pathway	8	3.686635945	0.33091415	KLHL7, G2E3, PIAS2, TRIM25, RNF187, DYRK2, USP45, FBXO11	203	631	22680	1.416470845	1	0.786917	99.41481273
GOTERM_MF_DIRECT	GO:0016874~ligase activity	5	2.304147465	0.438999491	G2E3, PIAS2, TRIM25, RNF187, ACSL4	164	362	17446	1.469310066	1	0.99593674	99.96663204

Annotation Cluster 27	Enrichment Score: 0.4688344027411168											
Category	Term	Count	%	PValue	Genes	List Total	Pop Hits	Pop Total	Fold Enrichment	Bonferroni	Benjamini	FDR

GOTERM_MF_DIRECT	GO:0016791~phosphatase activity	3	1.382488479	0.302170782	MTM1, PTPDC1, PPP1CB	164	118	17446	2.704526664	1	0.98225519	99.3144438
UP_KEYWORDS	Protein phosphatase	3	1.382488479	0.322498424	MTM1, PTPDC1, PPP1CB	203	130	22680	2.578249337	1	0.78211971	99.31333621
GOTERM_BP_DIRECT	GO:0006470~protein dephosphorylation	3	1.382488479	0.36494186	MTM1, PTPDC1, PPP1CB	168	138	18082	2.339803313	1	0.99994594	99.92867826
GOTERM_MF_DIRECT	GO:0004721~phosphoprotein phosphatase activity	3	1.382488479	0.374678387	MTM1, PTPDC1, PPP1CB	164	139	17446	2.29592911	1	0.9909704	99.84996736

Annotation Cluster 28	Enrichment Score: 0.44957759925481633											
Category	Term	Count	%	PValue	Genes	List Total	Pop Hits	Pop Total	Fold Enrichment	Bonferroni	Benjamini	FDR
KEGG_PATHWAY	mmu05200:Pathways in cancer	8	3.686635945	0.075318647	PRKCA, EDNRB, CDC42, GNAI3, ROCK1, IKBKG, RB1, KITL	73	395	7691	2.133795734	0.999997737	0.80305903	61.38308727
KEGG_PATHWAY	mmu04014:Ras signaling pathway	4	1.843317972	0.36289844	PRKCA, CDC42, IKBKG, KITL	73	229	7691	1.840282347	1	0.92427079	99.58218601
KEGG_PATHWAY	mmu04010:MAPK signaling pathway	3	1.382488479	0.686877414	PRKCA, CDC42, IKBKG	73	251	7691	1.259237025	1	0.99286238	99.99992543
KEGG_PATHWAY	mmu04151:PI3K-Akt signaling pathway	3	1.382488479	0.847466923	PRKCA, IKBKG, KITL	73	351	7691	0.900480037	1	0.99950612	99.99999999

Annotation Cluster 29	Enrichment Score: 0.4415603664914874											
Category	Term	Count	%	PValue	Genes	List Total	Pop Hits	Pop Total	Fold Enrichment	Bonferroni	Benjamini	FDR
UP_KEYWORDS	Magnesium	12	5.529953917	0.007258558	ATP7A, NUDT18, GNAI3, ROCK1, ATP10A, IDH1, NT5DC3, DYRK2, IDI1, RIOK1, ACSL4, LATS2	203	521	22680	2.573300682	0.808659697	0.15242011	8.898992736
INTERPRO	IPR000961:AGC-kinase, C-terminal	3	1.382488479	0.085194132	PRKCA, ROCK1, LATS2	190	53	20594	6.135253227	1	0.94767624	72.22897431
SMART	SM00133:S_TK_X	3	1.382488479	0.089230935	PRKCA, ROCK1, LATS2	117	45	10425	5.94017094	0.999996688	0.83512321	66.5716875
UP_KEYWORDS	Transferase	21	9.677419355	0.100999022	PRKCA, SOAT1, ALDH18A1, ROCK1, SGMS2, DPY19L4, TRIM25, WHSC1, RIOK1, ST6GALNAC2, LATS2, ST6GALNAC1, TNKS2, MGAT2, G2E3, CDYL, STT3A, PRKAR1B, IKBKG, DYRK2, CLOCK	203	1654	22680	1.418504774	1	0.50877428	74.38886806
UP_KEYWORDS	Nucleotide-binding	22	10.13824885	0.101800003	PRKCA, RAB2A, GTPBP4, ALDH18A1, GNAI3, ROCK1, RP2, ATP10A, HBS1L, SPAG1, RIOK1, MYH9, TIMM44, LATS2, ATP7A, CDC42, PSMC6, PRKAR1B, TUBB5, DYRK2, CRY1, ACSL4	203	1754	22680	1.401328982	1	0.50158521	74.67927099
UP_SEQ_FEATURE	domain:AGC-kinase C-terminal	3	1.382488479	0.105633715	PRKCA, ROCK1, LATS2	182	55	18012	5.398201798	1	0.99559801	81.59862836
GOTERM_MF_DIRECT	GO:0000166~nucleotide binding	23	10.59907834	0.193776887	PRKCA, RAB2A, GTPBP4, ALDH18A1, GNAI3, ROCK1, RP2, ATP10A, HBS1L, SPAG1, RIOK1, MYH9, TIMM44, LATS2, ATP7A, CDC42, PSMC6, SRSF7, PRKAR1B, TUBB5, DYRK2, CRY1, ACSL4	164	1936	17446	1.263788803	1	0.95741962	94.93621351
GOTERM_MF_DIRECT	GO:0016301~kinase activity	9	4.147465438	0.295323325	PRKCA, ALDH18A1, SGMS2, ROCK1, PRKAR1B, IKBKG, DYRK2, RIOK1, LATS2	164	674	17446	1.420478396	1	0.98242181	99.21517249
UP_KEYWORDS	Kinase	9	4.147465438	0.295706941	PRKCA, ALDH18A1, SGMS2, ROCK1, PRKAR1B, IKBKG, DYRK2, RIOK1, LATS2	203	707	22680	1.422230893	1	0.76469194	98.87221318
UP_KEYWORDS	Apoptosis	6	2.764976959	0.450342416	PRKCA, G2E3, ROCK1, LGALS7, DYRK2, THOC1	203	489	22680	1.370848318	1	0.8484456	99.9527002
UP_KEYWORDS	Serine/threonine-protein kinase	5	2.304147465	0.487156567	PRKCA, ROCK1, DYRK2, RIOK1, LATS2	203	405	22680	1.379310345	1	0.85678734	99.98051597
GOTERM_BP_DIRECT	GO:0016310~phosphorylation	7	3.225806452	0.499368459	PRKCA, ALDH18A1, SGMS2, ROCK1, DYRK2, RIOK1, LATS2	168	612	18082	1.231072985	1	0.99999691	99.99839751
GOTERM_BP_DIRECT	GO:0035556~intracellular signal transduction	5	2.304147465	0.506934777	PRKCA, PDZD8, VAV3, ROCK1, LATS2	168	400	18082	1.345386905	1	0.9999971	99.99874325
GOTERM_MF_DIRECT	GO:0004674~protein serine/threonine kinase activity	5	2.304147465	0.569637877	PRKCA, ROCK1, DYRK2, RIOK1, LATS2	164	428	17446	1.242734215	1	0.99907276	99.99915106

INTERPRO	IPR008271:Serine/threonine-protein kinase, active site	4	1.843317972	0.592212528	PRKCA, ROCK1, DYRK2, LATS2	190	333	20594	1.30197566	1	0.99999965	99.99975178
UP_KEYWORDS	ATP-binding	12	5.529953917	0.675028584	PRKCA, ATP7A, PSMC6, ALDH18A1, ROCK1, ATP10A, DYRK2, MYH9, RIOK1, ACSL4, TIMM44, LATS2	203	1363	22680	0.983631442	1	0.93942477	99.99994314
INTERPRO	IPR017441:Protein kinase, ATP binding site	4	1.843317972	0.704105179	PRKCA, ROCK1, DYRK2, LATS2	190	394	20594	1.100400748	1	0.99999999	99.99999754
UP_SEQ_FEATURE	domain:Protein kinase	5	2.304147465	0.746563262	PRKCA, ROCK1, DYRK2, RIOK1, LATS2	182	502	18012	0.98572742	1	1	99.99999991
INTERPRO	IPR011009:Protein kinase-like domain	5	2.304147465	0.75425529	PRKCA, ROCK1, DYRK2, RIOK1, LATS2	190	556	20594	0.974725483	1	1	99.99999983
GOTERM_MF_DIRECT	GO:0005524~ATP binding	13	5.99078341	0.759403674	PRKCA, ATP7A, PSMC6, ALDH18A1, ENPP1, ROCK1, ATP10A, DYRK2, RIOK1, MYH9, ACSL4, TIMM44, LATS2	164	1507	17446	0.917660673	1	0.99992682	99.99999973
GOTERM_BP_DIRECT	GO:0006468~protein phosphorylation	5	2.304147465	0.782937975	PRKCA, ROCK1, PRKAR1B, DYRK2, LATS2	168	576	18082	0.934296462	1	1	100
SMART	SM00220:S_TKc	4	1.843317972	0.800448894	PRKCA, ROCK1, DYRK2, LATS2	117	380	10425	0.937921727	1	0.99999995	99.99999938
UP_SEQ_FEATURE	binding site:ATP	5	2.304147465	0.84155419	PRKCA, ROCK1, DYRK2, RIOK1, LATS2	182	583	18012	0.848773868	1	1	100
UP_SEQ_FEATURE	active site:Proton acceptor	6	2.764976959	0.845676812	PRKCA, L3HYPDH, ROCK1, DYRK2, RIOK1, LATS2	182	710	18012	0.836341124	1	1	100
INTERPRO	IPR000719:Protein kinase, catalytic domain	4	1.843317972	0.854955828	PRKCA, ROCK1, DYRK2, LATS2	190	515	20594	0.84185999	1	1	100
GOTERM_MF_DIRECT	GO:0004672~protein kinase activity	4	1.843317972	0.877133437	PRKCA, ROCK1, DYRK2, LATS2	164	531	17446	0.801341234	1	0.99999868	100
UP_SEQ_FEATURE	nucleotide phosphate-binding region:ATP	7	3.225806452	0.926255081	PRKCA, PSMC6, ROCK1, DYRK2, MYH9, TIMM44, LATS2	182	963	18012	0.719386532	1	1	100

Annotation Cluster 30	Enrichment Score: 0.4210557501635246											
Category	Term	Count	%	PValue	Genes	List Total	Pop Hits	Pop Total	Fold Enrichment	Bonferroni	Benjamini	FDR
GOTERM_CC_DIRECT	GO:0016020~membrane	79	36.40552995	0.021674932	TMEM18, TM7SF3, SGMS2, SLC16A11, RP2, LEPR, ORMDL1, LYPD8, ATP10A, HBS1L, GJA1, DPY19L4, SDC2, CDC42, EDNRB, ANK3, DNAJC3, PRKCA, GTPBP4, ROCK1, MMP19, MPP6, MYH9, GRHL2, TIMM44, BICD1, RNF225, ST6GALNAC2, ST6GALNAC1, TNKS2, MGAT2, SDC1, CPE, PLXDC1, CLDN1, CPD, STEAP2, MED1, ALDH18A1, DERL1, MTDH, GNAI3, ENPP1, ABHD3, SLC11A2, MTM1, SLC48A1, STT3A, C1QTNF3, MFAP3L, ACSL4, PCSK5, CRIM1, SOAT1, RAB2A, 2010107G23RIK, TRPC6, LRRN4, CKAP4, AF529169, TSPAN13, AP4S1, HERPUD2, REEP3, KITL, TPBG, ATP7A, PLEKHA3, PSMC6, PDZD8, TMEM263, PRKAR1B, ANXA13, MARCKS, DSC1, YIPF4, GFRA2, GCA, VLDLR	182	6998	19662	1.21957765	0.99468528	0.52677397	24.62228433
UP_SEQ_FEATURE	topological domain:Cytoplasmic	32	14.74654378	0.367667	TMEM18, DERL1, MTDH, SGMS2, ENPP1, SLC16A11, LEPR, ATP10A, GJA1, SDC2, SLC11A2, EDNRB, STT3A, MFAP3L, ACSL4, PCSK5, CRIM1, TRPC6, LRRN4, TSPAN13, KITL, TPBG, ST6GALNAC2, ST6GALNAC1, ATP7A, MGAT2, SDC1, PLXDC1, CLDN1, DSC1, CPD, VLDLR	182	2880	18012	1.0996337	1	0.99995994	99.90409709

UP_SEQ_FEATURE	transmembrane region	46	21.19815668	0.413358129	MTDH, DERL1, TMEM18, TM7SF3, ENPP1, SGMS2, SLC16A11, ORMDL1, LEPR, ATP10A, GJA1, ABHD3, DPY19L4, SDC2, SLC11A2, EDNRB, STT3A, SLC48A1, MFAP3L, ACSL4, PCSK5, CRIM1, SOAT1, 2010107G23RIK, TRPC6, LRRN4, AF529169, CKAP4, TSPAN13, TPBG, KITL, HERPUD2, REEP3, ST6GALNAC2, ST6GALNAC1, ATP7A, MGAT2, SDC1, TMEM263, PLXDC1, CLDN1, DSC1, YIPF4, CPD, STEAP2, VLDLR	182	4312	18012	1.05577076	1	0.99998502	99.96924291
UP_KEYWORDS	Membrane	80	36.86635945	0.430660138	TMEM18, TM7SF3, SGMS2, SLC16A11, RP2, LEPR, LYPD8, ORMDL1, ATP10A, GJA1, DPY19L4, SDC2, PGR, CDC42, EDNRB, DMXL1, ANK3, PRKCA, TRPM3, ROCK1, MMP19, MPP6, GRHL2, TIMM44, BICD1, RNF225, ST6GALNAC2, TNKS2, ST6GALNAC1, MGAT2, SPAG9, SDC1, CPE, PLXDC1, CLDN1, PIAS2, CPD, STEAP2, MED1, ALDH18A1, DERL1, MTDH, GNAI3, ENPP1, 1810064F22RIK, ABHD3, SLC11A2, MTM1, CDYL, SLC48A1, STT3A, 4930512M02RIK, MFAP3L, ACSL4, PCSK5, CRIM1, SOAT1, RAB2A, 2010107G23RIK, TRPC6, LRRN4, CKAP4, AF529169, TSPAN13, AP4S1, HERPUD2, REEP3, KITL, TPBG, ATP7A, PLEKHA3, TMEM263, PRKAR1B, ANXA13, MARCKS, DSC1, YIPF4, GFRA2, GCA, VLDLR	203	8683	22680	1.029359787	1	0.83485015	99.92581152
UP_SEQ_FEATURE	topological domain:Extracellular	24	11.05990783	0.504237293	TMEM18, TRPC6, LRRN4, ENPP1, SLC16A11, LEPR, ATP10A, TSPAN13, GJA1, KITL, TPBG, SDC2, ATP7A, SLC11A2, EDNRB, SDC1, PLXDC1, CLDN1, DSC1, MFAP3L, CPD, PCSK5, CRIM1, VLDLR	182	2256	18012	1.052840776	1	0.99999799	99.99760351
UP_KEYWORDS	Transmembrane helix	57	26.26728111	0.832650859	TM7SF3, TMEM18, SGMS2, SLC16A11, ORMDL1, LEPR, ATP10A, GJA1, DPY19L4, SDC2, PGR, EDNRB, DMXL1, TRPM3, ST6GALNAC2, RNF225, BICD1, ST6GALNAC1, MGAT2, SPAG9, SDC1, PLXDC1, CLDN1, PIAS2, CPD, STEAP2, MED1, MTDH, DERL1, ENPP1, 1810064F22RIK, ABHD3, SLC11A2, STT3A, SLC48A1, CDYL, 4930512M02RIK, MFAP3L, ACSL4, PCSK5, CRIM1, SOAT1, 2010107G23RIK, TRPC6, LRRN4, AF529169, CKAP4, TSPAN13, KITL, REEP3, TPBG, HERPUD2, ATP7A, TMEM263, DSC1, YIPF4, VLDLR	203	6938	22680	0.91788352	1	0.9847553	99.99999999
UP_KEYWORDS	Transmembrane	57	26.26728111	0.838350923	TM7SF3, TMEM18, SGMS2, SLC16A11, ORMDL1, LEPR, ATP10A, GJA1, DPY19L4, SDC2, PGR, EDNRB, DMXL1, TRPM3, ST6GALNAC2, RNF225, BICD1, ST6GALNAC1, MGAT2, SPAG9, SDC1, PLXDC1, CLDN1, PIAS2, CPD, STEAP2, MED1, MTDH, DERL1, ENPP1, 1810064F22RIK, ABHD3, SLC11A2, STT3A, SLC48A1, CDYL, 4930512M02RIK, MFAP3L, ACSL4, PCSK5, CRIM1, SOAT1, 2010107G23RIK, TRPC6, LRRN4, AF529169, CKAP4, TSPAN13, KITL, REEP3, TPBG, HERPUD2, ATP7A, TMEM263, DSC1, YIPF4, VLDLR	203	6955	22680	0.915639951	1	0.9853176	99.99999999

GOTERM_CC_DIRECT	GO:0016021~integral component of membrane	58	26.7281106	0.857347117	TM7SF3, TMEM18, SGMS2, SLC16A11, ORMDL1, LEPR, ATP10A, GJA1, DPY19L4, SDC2, PGR, EDNRB, DMXL1, TRPM3, MPP6, BICD1, ST6GALNAC2, RNF225, ST6GALNAC1, MGAT2, SPAG9, SDC1, PLXDC1, CLDN1, PIAS2, CPD, STEAP2, MED1, MTDH, DERL1, ENPP1, 1810064F22RIK, ABHD3, SLC11A2, STT3A, SLC48A1, CDYL, 4930512M02RIK, MFAP3L, ACSL4, PCSK5, CRIM1, SOAT1, 2010107G23RIK, TRPC6, LRRN4, AF529169, CKAP4, TSPAN13, KITL, REEP3, TPBG, HERPUD2, ATP7A, TMEM263, DSC1, YIPF4, VLDLR	182	6878	19662	0.911007864	1	0.9994506	100

Annotation Cluster 31 Enrichment Score: 0.41525289306247476

Category	Term	Count	%	PValue	Genes	List Total	Pop Hits	Pop Total	Fold Enrichment	Bonferroni	Benjamini	FDR
UP_KEYWORDS	GPI-anchor	3	1.382488479	0.354918064	LYPD8, MMP19, GFRA2	203	140	22680	2.39408867	1	0.80433296	99.63330904
GOTERM_CC_DIRECT	GO:0031225~anchored component of membrane	3	1.382488479	0.372868333	LYPD8, MMP19, GFRA2	182	141	19662	2.298573767	1	0.92523617	99.7567017
UP_SEQ_FEATURE	propeptide:Removed in mature form	4	1.843317972	0.429099211	CDC42, LYPD8, MMP19, GFRA2	182	238	18012	1.663311478	1	0.99998402	99.97963713

Annotation Cluster 32 Enrichment Score: 0.3888282887754356

Category	Term	Count	%	PValue	Genes	List Total	Pop Hits	Pop Total	Fold Enrichment	Bonferroni	Benjamini	FDR
GOTERM_BP_DIRECT	GO:0015031~protein transport	8	3.686635945	0.307037085	RAB2A, MTM1, DERL1, RP2, A230046K03RIK, MYH9, AP4S1, TIMM44	168	592	18082	1.454472329	1	0.99978475	99.71292927
UP_KEYWORDS	Protein transport	7	3.225806452	0.409079835	RAB2A, MTM1, DERL1, RP2, A230046K03RIK, AP4S1, TIMM44	203	577	22680	1.355405486	1	0.82729321	99.88058895
GOTERM_BP_DIRECT	GO:0006810~transport	19	8.755760369	0.418242118	RAB2A, DERL1, TRPC6, RP2, SLC16A11, ATP10A, A230046K03RIK, AP4S1, TIMM44, BICD1, QK, ATP7A, SLC11A2, MTM1, SLC48A1, SRSF7, STEAP2, THOC1, VLDLR	168	1822	18082	1.122386441	1	0.99998067	99.98239375
UP_KEYWORDS	Transport	18	8.294930876	0.529980845	RAB2A, DERL1, TRPC6, RP2, SLC16A11, ATP10A, A230046K03RIK, AP4S1, TIMM44, QK, ATP7A, SLC11A2, MTM1, SLC48A1, SRSF7, STEAP2, THOC1, VLDLR	203	1901	22680	1.057882421	1	0.87316013	99.99361447

Annotation Cluster 33 Enrichment Score: 0.37345662827777737

Category	Term	Count	%	PValue	Genes	List Total	Pop Hits	Pop Total	Fold Enrichment	Bonferroni	Benjamini	FDR
UP_KEYWORDS	mRNA transport	3	1.382488479	0.178485586	SRSF7, THOC1, QK	203	86	22680	3.897353649	1	0.66328714	91.91600714
GOTERM_BP_DIRECT	GO:0051028~mRNA transport	3	1.382488479	0.195303577	SRSF7, THOC1, QK	168	88	18082	3.669237013	1	0.99919485	96.88082059
KEGG_PATHWAY	mmu03040:Spliceosome	3	1.382488479	0.354754166	SRSF7, SF3B5, THOC1	73	133	7691	2.376454836	1	0.92553669	99.51245665
UP_KEYWORDS	mRNA splicing	4	1.843317972	0.362845372	SRSF7, SF3B5, THOC1, QK	203	240	22680	1.862068966	1	0.80291209	99.68696104
GOTERM_BP_DIRECT	GO:0008380~RNA splicing	4	1.843317972	0.384879722	SRSF7, SF3B5, THOC1, QK	168	241	18082	1.786405849	1	0.99996668	99.957131
UP_KEYWORDS	mRNA processing	4	1.843317972	0.517317975	SRSF7, SF3B5, THOC1, QK	203	307	22680	1.455689094	1	0.87014159	99.99102766
GOTERM_BP_DIRECT	GO:0006397~mRNA processing	4	1.843317972	0.57392634	SRSF7, SF3B5, THOC1, QK	168	322	18082	1.337030464	1	0.99999957	99.99987777
UP_KEYWORDS	RNA-binding	4	1.843317972	0.906113967	SRSF7, PUM3, THOC1, QK	203	601	22680	0.743588272	1	0.99559195	100
GOTERM_MF_DIRECT	GO:0003723~RNA binding	5	2.304147465	0.937143994	SRSF7, HBP1, PUM3, THOC1, QK	164	780	17446	0.681910569	1	0.99999997	100

Annotation Cluster 34 Enrichment Score: 0.3544569882159475

Category	Term	Count	%	PValue	Genes	List Total	Pop Hits	Pop Total	Fold Enrichment	Bonferroni	Benjamini	FDR
UP_KEYWORDS	NADP	4	1.843317972	0.205756272	ALDH18A1, IDH1, ALDH1L2, CRYM	203	175	22680	2.553694581	1	0.67915615	94.7512634
GOTERM_MF_DIRECT	GO:0016491~oxidoreductase activity	7	3.225806452	0.49786538	KDM1B, ALDH18A1, IDH1, STEAP2, ALDH1L2, CRYM, KDM5D	164	604	17446	1.232858181	1	0.99776577	99.9928124
UP_KEYWORDS	Oxidoreductase	7	3.225806452	0.503834172	KDM1B, ALDH18A1, IDH1, STEAP2, ALDH1L2, CRYM, KDM5D	203	639	22680	1.223895095	1	0.86652227	99.98723592
INTERPRO	IPR016040:NAD(P)-binding domain	3	1.382488479	0.548173386	KDM1B, STEAP2, CRYM	190	199	20594	1.634012166	1	0.99999955	99.99891444
GOTERM_BP_DIRECT	GO:0055114~oxidation-reduction process	7	3.225806452	0.597096441	KDM1B, ALDH18A1, IDH1, STEAP2, ALDH1L2, CRYM, KDM5D	168	676	18082	1.114521696	1	0.99999966	99.99994992
Annotation Cluster 35	Enrichment Score: 0.30457555472161957											
Category	Term	Count	%	PValue	Genes	List Total	Pop Hits	Pop Total	Fold Enrichment	Bonferroni	Benjamini	FDR
GOTERM_BP_DIRECT	GO:0051301~cell division	6	2.764976959	0.264345614	CDC42, GNAI3, RB1, REEP3, PPP1CB, LATS2	168	374	18082	1.726699771	1	0.99937735	99.25470233
UP_KEYWORDS	Cell division	5	2.304147465	0.422212952	CDC42, GNAI3, REEP3, PPP1CB, LATS2	203	372	22680	1.501668521	1	0.83546792	99.91042916
UP_KEYWORDS	Cell cycle	6	2.764976959	0.65855961	CDC42, GNAI3, RB1, REEP3, PPP1CB, LATS2	203	626	22680	1.070838383	1	0.93548053	99.99989298
GOTERM_BP_DIRECT	GO:0007049~cell cycle	5	2.304147465	0.822998471	GNAI3, RB1, REEP3, PPP1CB, LATS2	168	614	18082	0.876473554	1	1	100
Annotation Cluster 36	Enrichment Score: 0.2631022923483371											
Category	Term	Count	%	PValue	Genes	List Total	Pop Hits	Pop Total	Fold Enrichment	Bonferroni	Benjamini	FDR
INTERPRO	IPR017986:WD40-repeat-containing domain	5	2.304147465	0.311383294	SPAG9, DMXL1, WDR77, WDR95, PWP1	190	307	20594	1.765300874	1	0.99994221	99.53357286
INTERPRO	IPR015943:WD40/YVTN repeat-like-containing domain	5	2.304147465	0.379852489	SPAG9, DMXL1, WDR77, WDR95, PWP1	190	340	20594	1.593962848	1	0.99997948	99.89663573
INTERPRO	IPR019775:WD40 repeat, conserved site	3	1.382488479	0.433537897	WDR77, WDR95, PWP1	190	161	20594	2.019679634	1	0.99999536	99.9719091
INTERPRO	IPR001680:WD40 repeat	4	1.843317972	0.434830528	DMXL1, WDR77, WDR95, PWP1	190	263	20594	1.648509105	1	0.99999262	99.97281746
SMART	SM00320:WD40	4	1.843317972	0.561414795	DMXL1, WDR77, WDR95, PWP1	117	262	10425	1.36034449	1	0.99998529	99.9936387
UP_SEQ_FEATURE	repeat:WD 5	3	1.382488479	0.628691023	DMXL1, WDR77, PWP1	182	211	18012	1.407114213	1	0.99999997	99.99997007
UP_KEYWORDS	WD repeat	3	1.382488479	0.667547373	DMXL1, WDR77, PWP1	203	256	22680	1.309267241	1	0.93781349	99.99992393
UP_SEQ_FEATURE	repeat:WD 4	3	1.382488479	0.675168241	DMXL1, WDR77, PWP1	182	230	18012	1.290874343	1	0.99999999	99.99999606
UP_SEQ_FEATURE	repeat:WD 3	3	1.382488479	0.704182272	DMXL1, WDR77, PWP1	182	243	18012	1.221815222	1	1	99.99999905
UP_SEQ_FEATURE	repeat:WD 1	3	1.382488479	0.714757226	DMXL1, WDR77, PWP1	182	248	18012	1.19718185	1	1	99.99999945
UP_SEQ_FEATURE	repeat:WD 2	3	1.382488479	0.714757226	DMXL1, WDR77, PWP1	182	248	18012	1.19718185	1	1	99.99999945
Annotation Cluster 37	Enrichment Score: 0.20007028694835405											
Category	Term	Count	%	PValue	Genes	List Total	Pop Hits	Pop Total	Fold Enrichment	Bonferroni	Benjamini	FDR
UP_SEQ_FEATURE	DNA-binding region:Basic motif	3	1.382488479	0.470432457	MAF, NPAS2, CLOCK	182	156	18012	1.903212172	1	0.99999495	99.99348455
GOTERM_BP_DIRECT	GO:0045944~positive regulation of transcription from RNA polymerase II promoter	10	4.608294931	0.574095271	MAF, PGR, NPAS2, IKBKG, PIAS2, RB1, GRHL2, CLOCK, CTR9, MED1	168	995	18082	1.081718114	1	0.99999946	99.99987854
GOTERM_MF_DIRECT	GO:0003700~transcription factor activity, sequence-specific DNA binding	8	3.686635945	0.72408729	MAF, PGR, POU6F1, NPAS2, RCOR1, ZFX, GRHL2, CLOCK	164	883	17446	0.963787531	1	0.99984323	99.9999982
UP_KEYWORDS	Activator	5	2.304147465	0.809928499	MAF, NPAS2, ZFX, CLOCK, MED1	203	624	22680	0.895225464	1	0.98027854	99.99999994
Annotation Cluster 38	Enrichment Score: 0.19648793309579413											
Category	Term	Count	%	PValue	Genes	List Total	Pop Hits	Pop Total	Fold Enrichment	Bonferroni	Benjamini	FDR

UP_KEYWORDS	Differentiation	7	3.225806452	0.507555666	CFAP54, CDC42, CHRDL1, DMKN, MMP19, SDC2, QK	203	646	22680	1.210633074	1	0.86601277	99.98840799
UP_KEYWORDS	Neurogenesis	3	1.382488479	0.648312247	CDC42, CHRDL1, SDC2	203	247	22680	1.356973335	1	0.93660336	99.99984378
GOTERM_BP_DIRECT	GO:0007399~nervous system development	4	1.843317972	0.679968019	CDC42, CHRDL1, SDC2, GFRA2	168	377	18082	1.14197297	1	0.99999996	99.99999873
GOTERM_BP_DIRECT	GO:0030154~cell differentiation	7	3.225806452	0.731630623	CFAP54, CDC42, CHRDL1, DMKN, MMP19, SDC2, QK	168	780	18082	0.965918803	1	1	99.99999992
Annotation Cluster 39	Enrichment Score: 0.18987995941480273											
Category	Term	Count	%	PValue	Genes	List Total	Pop Hits	Pop Total	Fold Enrichment	Bonferroni	Benjamini	FDR
INTERPRO	IPR011993:Pleckstrin homology-like domain	5	2.304147465	0.518246439	PLEKHA3, MTM1, VAV3, ROCK1, MTMR9	190	409	20594	1.325054691	1	0.99999913	99.99726843
UP_SEQ_FEATURE	domain:PH	3	1.382488479	0.620900602	PLEKHA3, VAV3, ROCK1	182	208	18012	1.427409129	1	0.99999997	99.999959
INTERPRO	IPR001849:Pleckstrin homology domain	3	1.382488479	0.695885086	PLEKHA3, VAV3, ROCK1	190	262	20594	1.241100844	1	0.99999999	99.99999635
SMART	SM00233:PH	3	1.382488479	0.776934176	PLEKHA3, VAV3, ROCK1	117	253	10425	1.056552144	1	0.99999995	99.9999977
Annotation Cluster 40	Enrichment Score: 0.170325717074493											
Category	Term	Count	%	PValue	Genes	List Total	Pop Hits	Pop Total	Fold Enrichment	Bonferroni	Benjamini	FDR
GOTERM_CC_DIRECT	GO:0043005~neuron projection	5	2.304147465	0.541521407	PRKCA, ATP7A, PGR, CDC42, ANK3	182	420	19662	1.28610675	1	0.97412692	99.99572073
GOTERM_CC_DIRECT	GO:0030425~dendrite	5	2.304147465	0.663885335	PRKCA, PGR, CPE, ANK3, PLXDC1	182	490	19662	1.102377215	1	0.98700304	99.99992198
GOTERM_CC_DIRECT	GO:0030424~axon	3	1.382488479	0.857658001	PRKCA, PGR, ANK3	182	370	19662	0.875942976	1	0.99938618	100
Annotation Cluster 41	Enrichment Score: 0.10216027748024295											
Category	Term	Count	%	PValue	Genes	List Total	Pop Hits	Pop Total	Fold Enrichment	Bonferroni	Benjamini	FDR
GOTERM_CC_DIRECT	GO:0005887~integral component of plasma membrane	11	5.069124424	0.592482415	ATP7A, SLC11A2, TRPC6, LRRN4, ENPP1, SGMS2, SLC16A11, LEPR, CLDN1, TSPAN13, STEAP2	182	1126	19662	1.055384225	1	0.97977406	99.99906396
GOTERM_BP_DIRECT	GO:0006811~ion transport	4	1.843317972	0.909482171	ATP7A, SLC11A2, TRPC6, STEAP2	168	584	18082	0.737198304	1	1	100
UP_KEYWORDS	Ion transport	4	1.843317972	0.916324951	ATP7A, SLC11A2, TRPC6, STEAP2	203	619	22680	0.72196535	1	0.99641662	100
Annotation Cluster 42	Enrichment Score: 0.06481855279125175											
Category	Term	Count	%	PValue	Genes	List Total	Pop Hits	Pop Total	Fold Enrichment	Bonferroni	Benjamini	FDR
INTERPRO	IPR015880:Zinc finger, C2H2-like	6	2.764976959	0.766273187	2610044O15RIK8, ZFP944, ZFX, ZFP800, ZFP455, ZFP277	190	693	20594	0.938437002	1	1	99.99999992
INTERPRO	IPR007087:Zinc finger, C2H2	6	2.764976959	0.805037269	2610044O15RIK8, ZFP944, ZFX, ZFP800, ZFP455, ZFP277	190	731	20594	0.889653683	1	1	99.99999999
INTERPRO	IPR013087:Zinc finger C2H2-type/integrase DNA-binding domain	5	2.304147465	0.852847351	2610044O15RIK8, ZFP944, ZFX, ZFP800, ZFP455	190	652	20594	0.83120762	1	1	100
INTERPRO	IPR001909:Krueppel-associated box	3	1.382488479	0.859461368	2610044O15RIK8, ZFP944, ZFP455	190	373	20594	0.871765204	1	1	100
SMART	SM00355:ZnF_C2H2	6	2.764976959	0.892181681	2610044O15RIK8, ZFP944, ZFX, ZFP800, ZFP455, ZFP277	117	693	10425	0.771450771	1	1	100
SMART	SM00349:KRAB	3	1.382488479	0.919262364	2610044O15RIK8, ZFP944, ZFP455	117	367	10425	0.728358835	1	1	100
GOTERM_MF_DIRECT	GO:0003676~nucleic acid binding	8	3.686635945	0.948593332	2610044O15RIK8, SRSF7, ENPP1, ZFP944, ZFX, ZFP800, ZFP455, QK	164	1237	17446	0.687974446	1	0.99999999	100
