# Supplementary material for: Predicted mouse interactome and network-based interpretation of differentially expressed genes
Source: PLoS One. 2022 Apr 7;17(4):e0264174. doi: 10.1371/journal.pone.0264174 (PMC8989236; doi:10.1371/journal.pone.0264174)
Supplement: S6 Table — (PDF) [file pone.0264174.s007.pdf]

Table S6. Functional annotations reported by GO enrichment analysis tool for the top 250 transcriptionally changed genes between the wild type and Piezo1-knockout mice.

Analysis Type:	PANTHER Overrepresentation Test (Released 20200407)						
Annotation Version and Release Date:	GO Ontology database DOI: 10.5281/zenodo.3873405 Released 2020-06-01						
Analyzed List:	upload_1 (Mus musculus)						
Reference List:	Mus musculus (all genes in database)						
Test Type:	FISHER						
Correction:	FDR						
GO biological process complete	Mus musculus - REFLIST (22265)	upload_1 (237)	upload_1 (expected)	upload_1 (over/under)	upload_1 (fold Enrichment)	upload_1 (raw P-value)	upload_1 (FDR)
response to vitamin K (GO:0032571)	5	3	0.05	+	56.37	6.21E-05	8.36E-03
peptidyl-lysine oxidation (GO:0018057)	5	3	0.05	+	56.37	6.21E-05	8.29E-03
chondrocyte proliferation (GO:0035988)	11	5	0.12	+	42.7	4.94E-07	1.38E-04
cardiac muscle fiber development (GO:0048739)	9	3	0.1	+	31.32	2.37E-04	2.28E-02
neuronal action potential propagation (GO:0019227)	11	3	0.12	+	25.62	3.85E-04	3.40E-02
action potential propagation (GO:0098870)	11	3	0.12	+	25.62	3.85E-04	3.38E-02
elastic fiber assembly (GO:0048251)	11	3	0.12	+	25.62	3.85E-04	3.36E-02
protein oxidation (GO:0018158)	13	3	0.14	+	21.68	5.84E-04	4.66E-02
negative regulation of substrate adhesion-dependent cell spreading (GO:1900025)	13	3	0.14	+	21.68	5.84E-04	4.64E-02
regulation of skeletal muscle contraction (GO:0014819)	13	3	0.14	+	21.68	5.84E-04	4.61E-02
positive regulation of fibroblast migration (GO:0010763)	20	4	0.21	+	18.79	1.08E-04	1.26E-02
collagen fibril organization (GO:0030199)	50	10	0.53	+	18.79	6.61E-10	3.75E-07
sarcomere organization (GO:0045214)	39	7	0.42	+	16.86	4.96E-07	1.36E-04
regulation of platelet-derived growth factor receptor signaling pathway (GO:0010640)	25	4	0.27	+	15.03	2.32E-04	2.26E-02
regulation of bone mineralization (GO:0030500)	82	13	0.87	+	14.89	2.30E-11	2.43E-08
muscle fiber development (GO:0048747)	59	9	0.63	+	14.33	3.93E-08	1.49E-05
endochondral bone growth (GO:0003416)	27	4	0.29	+	13.92	3.02E-04	2.76E-02
regulation of biomineral tissue development (GO:0070167)	99	14	1.05	+	13.29	1.53E-11	1.87E-08
regulation of biomineralization (GO:0110149)	100	14	1.06	+	13.15	1.73E-11	1.96E-08
embryonic eye morphogenesis (GO:0048048)	36	5	0.38	+	13.05	6.83E-05	8.90E-03
extracellular matrix assembly (GO:0085029)	29	4	0.31	+	12.96	3.86E-04	3.35E-02
embryonic camera-type eye morphogenesis (GO:0048596)	30	4	0.32	+	12.53	4.34E-04	3.67E-02
bone mineralization (GO:0030282)	46	6	0.49	+	12.25	1.74E-05	2.79E-03
bone growth (GO:0098868)	31	4	0.33	+	12.12	4.86E-04	4.06E-02
positive regulation of bone mineralization (GO:0030501)	42	5	0.45	+	11.18	1.33E-04	1.46E-02
regulation of fibroblast migration (GO:0010762)	42	5	0.45	+	11.18	1.33E-04	1.45E-02
myofibril assembly (GO:0030239)	59	7	0.63	+	11.15	6.04E-06	1.09E-03
regulation of sodium ion transmembrane transporter activity (GO:2000649)	51	6	0.54	+	11.05	2.97E-05	4.58E-03
smooth muscle contraction (GO:0006939)	55	6	0.59	+	10.25	4.38E-05	6.33E-03
extracellular matrix organization (GO:0030198)	267	29	2.84	+	10.2	8.79E-20	1.40E-15
extracellular structure organization (GO:0043062)	268	29	2.85	+	10.17	9.65E-20	7.66E-16
biomineralization (GO:0110148)	87	9	0.93	+	9.72	8.02E-07	1.96E-04
biomineral tissue development (GO:0031214)	87	9	0.93	+	9.72	8.02E-07	1.93E-04
striated muscle cell development (GO:0055002)	146	15	1.55	+	9.65	1.77E-10	1.17E-07
muscle contraction (GO:0006936)	186	19	1.98	+	9.6	6.69E-13	1.18E-09
positive regulation of biomineralization (GO:0110151)	50	5	0.53	+	9.39	2.81E-04	2.63E-02
positive regulation of biomineral tissue development (GO:0070169)	50	5	0.53	+	9.39	2.81E-04	2.61E-02
muscle cell development (GO:0055001)	161	16	1.71	+	9.34	6.78E-11	5.67E-08

regulation of sodium ion transmembrane transport (GO:1902305)	62	6	0.66	+	9.09	8.13E-05	1.02E-02
regulation of substrate adhesion-dependent cell spreading (GO:1900024)	54	5	0.57	+	8.7	3.91E-04	3.38E-02
cardiac muscle cell development (GO:0055013)	65	6	0.69	+	8.67	1.04E-04	1.22E-02
integrin-mediated signaling pathway (GO:0007229)	78	7	0.83	+	8.43	3.23E-05	4.84E-03
regulation of release of sequestered calcium ion into cytosol (GO:0051279)	79	7	0.84	+	8.32	3.49E-05	5.13E-03
proteoglycan metabolic process (GO:0006029)	68	6	0.72	+	8.29	1.31E-04	1.45E-02
cellular component assembly involved in morphogenesis (GO:0010927)	102	9	1.09	+	8.29	2.73E-06	5.48E-04
cardiac cell development (GO:0055006)	71	6	0.76	+	7.94	1.63E-04	1.67E-02
ossification (GO:0001503)	220	18	2.34	+	7.69	8.31E-11	6.28E-08
regulation of sequestering of calcium ion (GO:0051282)	112	9	1.19	+	7.55	5.56E-06	1.03E-03
muscle system process (GO:0003012)	241	19	2.57	+	7.41	4.42E-11	4.13E-08
striated muscle cell differentiation (GO:0051146)	219	17	2.33	+	7.29	6.00E-10	3.53E-07
actomyosin structure organization (GO:0031032)	109	8	1.16	+	6.9	3.42E-05	5.08E-03
regulation of phosphatidylinositol 3-kinase signaling (GO:0014066)	99	7	1.05	+	6.64	1.32E-04	1.46E-02
muscle cell differentiation (GO:0042692)	271	19	2.88	+	6.59	2.86E-10	1.82E-07
cardiac muscle cell differentiation (GO:0055007)	100	7	1.06	+	6.58	1.40E-04	1.50E-02
cardiocyte differentiation (GO:0035051)	130	9	1.38	+	6.5	1.71E-05	2.78E-03
regulation of tissue remodeling (GO:0034103)	87	6	0.93	+	6.48	4.55E-04	3.82E-02
striated muscle contraction (GO:0006941)	102	7	1.09	+	6.45	1.58E-04	1.66E-02
regulation of calcium ion transport into cytosol (GO:0010522)	103	7	1.1	+	6.38	1.67E-04	1.70E-02
osteoblast differentiation (GO:0001649)	90	6	0.96	+	6.26	5.39E-04	4.41E-02
regulation of sodium ion transport (GO:0002028)	92	6	0.98	+	6.13	6.02E-04	4.71E-02
bone development (GO:0060348)	200	13	2.13	+	6.11	4.52E-07	1.28E-04
regulation of ossification (GO:0030278)	225	14	2.4	+	5.85	2.68E-07	8.17E-05
cell-matrix adhesion (GO:0007160)	114	7	1.21	+	5.77	3.00E-04	2.76E-02
regulation of calcium ion transmembrane transport (GO:1903169)	164	10	1.75	+	5.73	1.66E-05	2.71E-03
regulation of muscle contraction (GO:0006937)	155	9	1.65	+	5.45	6.30E-05	8.34E-03
positive regulation of leukocyte migration (GO:0002687)	156	9	1.66	+	5.42	6.60E-05	8.67E-03
regulation of transmembrane transporter activity (GO:0022898)	266	15	2.83	+	5.3	3.30E-07	9.69E-05
regulation of cation transmembrane transport (GO:1904062)	355	20	3.78	+	5.29	3.45E-09	1.61E-06
sodium ion transport (GO:0006814)	143	8	1.52	+	5.26	2.05E-04	2.05E-02
muscle structure development (GO:0061061)	489	27	5.21	+	5.19	8.20E-12	1.09E-08
cartilage development (GO:0051216)	164	9	1.75	+	5.16	9.50E-05	1.13E-02
regulation of ion transmembrane transporter activity (GO:0032412)	257	14	2.74	+	5.12	1.21E-06	2.87E-04
regulation of transporter activity (GO:0032409)	276	15	2.94	+	5.11	5.14E-07	1.36E-04
supramolecular fiber organization (GO:0097435)	489	26	5.21	+	5	4.51E-11	3.98E-08
negative regulation of cellular response to growth factor stimulus (GO:0090288)	151	8	1.61	+	4.98	2.91E-04	2.69E-02
limb morphogenesis (GO:0035108)	170	9	1.81	+	4.97	1.23E-04	1.41E-02
appendage morphogenesis (GO:0035107)	170	9	1.81	+	4.97	1.23E-04	1.40E-02
cardiac muscle tissue development (GO:0048738)	190	10	2.02	+	4.94	5.45E-05	7.53E-03
regulation of cell-substrate adhesion (GO:0010810)	214	11	2.28	+	4.83	2.85E-05	4.43E-03
limb development (GO:0060173)	196	10	2.09	+	4.79	6.98E-05	9.01E-03
appendage development (GO:0048736)	196	10	2.09	+	4.79	6.98E-05	8.94E-03
regulation of leukocyte migration (GO:0002685)	217	11	2.31	+	4.76	3.21E-05	4.86E-03
skeletal system development (GO:0001501)	482	24	5.13	+	4.68	9.20E-10	5.04E-07
eye morphogenesis (GO:0048592)	169	8	1.8	+	4.45	5.96E-04	4.68E-02

muscle tissue development (GO:0060537)	338	16	3.6	+	4.45	1.22E-06	2.85E-04
regulation of metal ion transport (GO:0010959)	426	20	4.53	+	4.41	6.22E-08	2.30E-05
striated muscle tissue development (GO:0014706)	320	15	3.41	+	4.4	2.97E-06	5.89E-04
connective tissue development (GO:0061448)	218	10	2.32	+	4.31	1.61E-04	1.67E-02
regulation of muscle system process (GO:0090257)	243	11	2.59	+	4.25	8.52E-05	1.03E-02
heart development (GO:0007507)	563	25	5.99	+	4.17	3.75E-09	1.70E-06
regulation of calcium ion transport (GO:0051924)	275	12	2.93	+	4.1	5.71E-05	7.82E-03
regulation of ion transmembrane transport (GO:0034765)	483	21	5.14	+	4.08	9.95E-08	3.51E-05
regulation of transmembrane transport (GO:0034762)	576	25	6.13	+	4.08	5.80E-09	2.56E-06
cellular component morphogenesis (GO:0032989)	605	26	6.44	+	4.04	3.43E-09	1.65E-06
regulation of myeloid cell differentiation (GO:0045637)	211	9	2.25	+	4.01	5.66E-04	4.56E-02
regulation of cell size (GO:0008361)	211	9	2.25	+	4.01	5.66E-04	4.54E-02
regulation of actin filament-based process (GO:0032970)	412	17	4.39	+	3.88	3.37E-06	6.61E-04
skeletal system morphogenesis (GO:0048705)	246	10	2.62	+	3.82	4.06E-04	3.45E-02
positive regulation of cell motility (GO:2000147)	585	23	6.23	+	3.69	1.36E-07	4.59E-05
positive regulation of cell migration (GO:0030335)	562	22	5.98	+	3.68	2.77E-07	8.30E-05
positive regulation of locomotion (GO:0040017)	601	23	6.4	+	3.6	2.15E-07	6.84E-05
circulatory system development (GO:0072359)	916	35	9.75	+	3.59	1.12E-10	7.73E-08
positive regulation of cellular component movement (GO:0051272)	604	23	6.43	+	3.58	2.34E-07	7.29E-05
response to wounding (GO:0009611)	317	12	3.37	+	3.56	2.06E-04	2.04E-02
biological adhesion (GO:0022610)	847	32	9.02	+	3.55	1.00E-09	5.32E-07
growth (GO:0040007)	453	17	4.82	+	3.53	1.12E-05	1.91E-03
cell adhesion (GO:0007155)	837	31	8.91	+	3.48	2.96E-09	1.47E-06
negative regulation of locomotion (GO:0040013)	324	12	3.45	+	3.48	2.50E-04	2.36E-02
negative regulation of cellular component movement (GO:0051271)	324	12	3.45	+	3.48	2.50E-04	2.35E-02
actin filament-based process (GO:0030029)	569	21	6.06	+	3.47	1.30E-06	2.99E-04
regulation of hemopoiesis (GO:1903706)	407	15	4.33	+	3.46	4.51E-05	6.39E-03
cell projection morphogenesis (GO:0048858)	491	18	5.23	+	3.44	8.34E-06	1.47E-03
developmental growth (GO:0048589)	437	16	4.65	+	3.44	2.71E-05	4.26E-03
regulation of cellular component size (GO:0032535)	414	15	4.41	+	3.4	5.43E-05	7.57E-03
regulation of anatomical structure size (GO:0090066)	582	21	6.2	+	3.39	1.84E-06	4.05E-04
regulation of actin cytoskeleton organization (GO:0032956)	364	13	3.87	+	3.36	1.96E-04	1.98E-02
blood vessel morphogenesis (GO:0048514)	430	15	4.58	+	3.28	8.18E-05	1.02E-02
cell part morphogenesis (GO:0032990)	517	18	5.5	+	3.27	1.63E-05	2.69E-03
regulation of cell migration (GO:0030334)	920	32	9.79	+	3.27	6.92E-09	2.89E-06
regulation of cell morphogenesis involved in differentiation (GO:0010769)	351	12	3.74	+	3.21	5.00E-04	4.14E-02
regulation of ion transport (GO:0043269)	734	25	7.81	+	3.2	5.11E-07	1.38E-04
regulation of cell motility (GO:2000145)	970	33	10.33	+	3.2	6.61E-09	2.84E-06
regulation of cellular component movement (GO:0051270)	1060	36	11.28	+	3.19	1.30E-09	6.66E-07
neuron projection morphogenesis (GO:0048812)	480	16	5.11	+	3.13	7.95E-05	1.00E-02
actin cytoskeleton organization (GO:0030036)	513	17	5.46	+	3.11	5.10E-05	7.16E-03
plasma membrane bounded cell projection morphogenesis (GO:0120039)	485	16	5.16	+	3.1	8.93E-05	1.07E-02
regulation of cell growth (GO:0001558)	425	14	4.52	+	3.09	2.49E-04	2.37E-02
regulation of locomotion (GO:0040012)	1017	33	10.83	+	3.05	2.00E-08	8.13E-06
negative regulation of cell population proliferation (GO:0008285)	690	22	7.34	+	3	7.09E-06	1.26E-03
blood vessel development (GO:0001568)	534	17	5.68	+	2.99	8.20E-05	1.01E-02
regulation of cell morphogenesis (GO:0022604)	537	17	5.72	+	2.97	8.75E-05	1.05E-02
regulation of neuron projection development (GO:0010975)	613	19	6.53	+	2.91	4.42E-05	6.32E-03

metal ion transport (GO:0030001)	554	17	5.9	+	2.88	1.26E-04	1.42E-02
vasculature development (GO:0001944)	564	17	6	+	2.83	1.55E-04	1.64E-02
cell migration (GO:0016477)	863	26	9.19	+	2.83	2.72E-06	5.55E-04
cell junction organization (GO:0034330)	501	15	5.33	+	2.81	4.04E-04	3.45E-02
tissue development (GO:0009888)	1647	49	17.53	+	2.79	7.36E-11	5.85E-08
cardiovascular system development (GO:0072358)	578	17	6.15	+	2.76	2.05E-04	2.06E-02
cation transport (GO:0006812)	723	21	7.7	+	2.73	4.36E-05	6.35E-03
animal organ morphogenesis (GO:0009887)	1013	29	10.78	+	2.69	1.86E-06	4.06E-04
cell activation (GO:0001775)	606	17	6.45	+	2.64	3.49E-04	3.15E-02
regulation of system process (GO:0044057)	608	17	6.47	+	2.63	3.62E-04	3.23E-02
cell morphogenesis (GO:0000902)	736	20	7.83	+	2.55	1.59E-04	1.66E-02
regulation of anatomical structure morphogenesis (GO:0022603)	1104	30	11.75	+	2.55	3.39E-06	6.56E-04
localization of cell (GO:0051674)	994	27	10.58	+	2.55	1.10E-05	1.91E-03
cell motility (GO:0048870)	994	27	10.58	+	2.55	1.10E-05	1.89E-03
regulation of growth (GO:0040008)	703	19	7.48	+	2.54	2.48E-04	2.37E-02
anatomical structure morphogenesis (GO:0009653)	2229	60	23.73	+	2.53	2.54E-11	2.52E-08
positive regulation of cell differentiation (GO:0045597)	1116	30	11.88	+	2.53	4.18E-06	7.99E-04
locomotion (GO:0040011)	1194	32	12.71	+	2.52	2.06E-06	4.41E-04
anatomical structure formation involved in morphogenesis (GO:0048646)	935	25	9.95	+	2.51	3.05E-05	4.66E-03
animal organ development (GO:0048513)	3068	82	32.66	+	2.51	1.05E-15	4.19E-12
regulation of plasma membrane bounded cell projection organization (GO:0120035)	787	21	8.38	+	2.51	1.39E-04	1.49E-02
regulation of multicellular organismal development (GO:2000026)	2189	58	23.3	+	2.49	9.06E-11	6.54E-08
regulation of cell projection organization (GO:0031344)	796	21	8.47	+	2.48	1.62E-04	1.67E-02
regulation of neuron differentiation (GO:0045664)	781	20	8.31	+	2.41	5.15E-04	4.23E-02
movement of cell or subcellular component (GO:0006928)	1416	36	15.07	+	2.39	1.58E-06	3.53E-04
positive regulation of cellular component organization (GO:0051130)	1234	31	13.14	+	2.36	1.17E-05	1.98E-03
regulation of localization (GO:0032879)	2923	73	31.11	+	2.35	2.89E-12	4.18E-09
regulation of transport (GO:0051049)	1991	49	21.19	+	2.31	3.71E-08	1.44E-05
positive regulation of developmental process (GO:0051094)	1556	38	16.56	+	2.29	2.31E-06	4.84E-04
regulation of multicellular organismal process (GO:0051239)	3276	79	34.87	+	2.27	1.08E-12	1.71E-09
cellular response to endogenous stimulus (GO:0071495)	879	21	9.36	+	2.24	6.42E-04	4.98E-02
regulation of cell differentiation (GO:0045595)	1901	45	20.24	+	2.22	5.25E-07	1.37E-04
system development (GO:0048731)	4274	101	45.49	+	2.22	2.25E-16	1.19E-12
regulation of cellular localization (GO:0060341)	1058	25	11.26	+	2.22	3.08E-04	2.80E-02
tube development (GO:0035295)	934	22	9.94	+	2.21	5.51E-04	4.47E-02
regulation of cell development (GO:0060284)	1104	26	11.75	+	2.21	2.24E-04	2.19E-02
regulation of cell population proliferation (GO:0042127)	1678	39	17.86	+	2.18	5.53E-06	1.03E-03
positive regulation of multicellular organismal process (GO:0051240)	1939	45	20.64	+	2.18	7.72E-07	1.94E-04
response to endogenous stimulus (GO:0009719)	1083	25	11.53	+	2.17	3.69E-04	3.27E-02
negative regulation of developmental process (GO:0051093)	1049	24	11.17	+	2.15	5.49E-04	4.47E-02
positive regulation of immune system process (GO:0002684)	1062	24	11.3	+	2.12	6.16E-04	4.79E-02
regulation of developmental process (GO:0050793)	2723	61	28.98	+	2.1	2.28E-08	9.06E-06
cell development (GO:0048468)	1770	39	18.84	+	2.07	1.79E-05	2.84E-03
multicellular organism development (GO:0007275)	4872	106	51.86	+	2.04	7.97E-15	2.53E-11
regulation of cellular component organization (GO:0051128)	2465	53	26.24	+	2.02	7.14E-07	1.83E-04
negative regulation of response to stimulus (GO:0048585)	1592	34	16.95	+	2.01	1.20E-04	1.38E-02
regulation of immune system process (GO:0002682)	1512	32	16.09	+	1.99	2.36E-04	2.29E-02
anatomical structure development (GO:0048856)	5248	110	55.86	+	1.97	2.30E-14	5.22E-11

regulation of biological quality (GO:0065008)	3926	82	41.79	+	1.96	5.86E-10	3.58E-07
developmental process (GO:0032502)	5618	114	59.8	+	1.91	5.27E-14	1.05E-10
cell differentiation (GO:0030154)	3726	73	39.66	+	1.84	1.10E-07	3.78E-05
cellular developmental process (GO:0048869)	3776	73	40.19	+	1.82	2.04E-07	6.63E-05
regulation of response to stimulus (GO:0048583)	3984	76	42.41	+	1.79	1.53E-07	5.07E-05
nervous system development (GO:0007399)	2303	43	24.51	+	1.75	3.58E-04	3.22E-02
multicellular organismal process (GO:0032501)	7345	137	78.18	+	1.75	1.04E-14	2.76E-11
regulation of molecular function (GO:0065009)	2567	47	27.32	+	1.72	2.17E-04	2.14E-02
regulation of signal transduction (GO:0009966)	2849	50	30.33	+	1.65	4.00E-04	3.43E-02
regulation of cell communication (GO:0010646)	3364	59	35.81	+	1.65	8.40E-05	1.03E-02
negative regulation of cellular process (GO:0048523)	4746	83	50.52	+	1.64	1.42E-06	3.23E-04
regulation of signaling (GO:0023051)	3378	59	35.96	+	1.64	1.19E-04	1.37E-02
cellular component organization (GO:0016043)	5084	88	54.12	+	1.63	7.98E-07	1.98E-04
response to chemical (GO:0042221)	3416	59	36.36	+	1.62	1.34E-04	1.45E-02
negative regulation of biological process (GO:0048519)	5204	88	55.39	+	1.59	2.21E-06	4.67E-04
cellular component organization or biogenesis (GO:0071840)	5282	88	56.22	+	1.57	5.07E-06	9.58E-04
positive regulation of biological process (GO:0048518)	6199	100	65.99	+	1.52	2.68E-06	5.52E-04
localization (GO:0051179)	4920	79	52.37	+	1.51	7.75E-05	9.84E-03
positive regulation of cellular process (GO:0048522)	5730	88	60.99	+	1.44	1.31E-04	1.46E-02
regulation of biological process (GO:0050789)	11668	163	124.2	+	1.31	3.95E-07	1.14E-04
biological regulation (GO:0065007)	12256	171	130.46	+	1.31	8.23E-08	2.97E-05
regulation of cellular process (GO:0050794)	11161	154	118.8	+	1.3	5.74E-06	1.05E-03
cellular nitrogen compound metabolic process (GO:0034641)	2789	13	29.69	-	0.44	4.90E-04	4.07E-02
sensory perception of chemical stimulus (GO:0007606)	1212	1	12.9	-	0.08	6.01E-05	8.16E-03
sensory perception of smell (GO:0007608)	1112	0	11.84	-	< 0.01	1.23E-05	2.05E-03
