# Supplementary material for: Predicted mouse interactome and network-based interpretation of differentially expressed genes
Source: PLoS One. 2022 Apr 7;17(4):e0264174. doi: 10.1371/journal.pone.0264174 (PMC8989236; doi:10.1371/journal.pone.0264174)
Supplement: S7 Table — (PDF) [file pone.0264174.s008.pdf]

Table S7. Functional annotations reported by DAVID for the top 250 transcriptionally changed genes between the wild type and Piezo1-knockout mice.

Annotation Cluster 1	Enrichment Score: 21.75562820599827											
Category	Term	Count	%	PValue	Genes	List Total	Pop Hits	Pop Total	Fold Enrichment	Bonferroni	Benjamini	FDR
GOTERM_CC_DIRECT	GO:0005578~proteinaceous extracellular matrix	43	17.2	1.18E-31	ASPN, WNT16, ADAMTSL1, MMP9, LUM, LTBP4, TGFB3, BCAN, POSTN, COL2A1, DCN, ABI3BP, CHAD, NOV, COL9A1, OGN, COL9A2, CILP2, GPC3, LECT1, COMP, ACAN, PTN, FBN2, LOX, LOXL2, COL8A2, IHH, COL10A1, DPT, MATN3, HAPLN1, HAPLN4, LGALS1, COL15A1, LAMA2, MMP10, OMD, COL14A1, FBLN2, EPYC, MFAP4, MFAP5	237	316	19662	11.28913636	2.93E-29	2.93E-29	1.53E-28
UP_KEYWORDS	Secreted	80	32	6.35E-31	ASPN, LTBP4, NELL1, MMP9, ARSI, IGFBP6, TGFB3, BCAN, POSTN, IGHM, CHAD, NOV, OGN, GPC3, CILP2, APOD, SEMA3D, SEMA3C, SEMA3B, LOXL4, LOX, HTRA3, TPSB2, LOXL2, IHH, COL10A1, MATN3, CPXM2, SERPING1, DHRS7C, PCOLCE2, MMP10, CEMIP, WFDC1, EPYC, MFAP4, MFAP5, MIA, WNT16, ADAMTSL1, LUM, COL2A1, DCN, OSTN, MDK, FAM19A5, C1QTNF9, ITGBL1, COL9A1, COL9A2, LECT1, IL17B, COMP, ACAN, TNFRSF19, PTN, FBN2, ANGPTL2, COL8A2, BGLAP2, DPT, HAPLN1, BGLAP, HAPLN4, VSTM4, LGALS1, COL15A1, IGF2, CLEC11A, LAMA2, DKK3, NBL1, OMD, COL14A1, NPY, FBLN2, CLEC3A, CMA1, MEGF6, PRSS23	242	1685	22680	4.449567158	1.57E-28	1.57E-28	8.24E-28
UP_KEYWORDS	Extracellular matrix	35	14	1.25E-28	ASPN, WNT16, ADAMTSL1, MMP9, LUM, LTBP4, BCAN, POSTN, COL2A1, DCN, CHAD, OGN, COL9A1, COL9A2, CILP2, LECT1, COMP, ACAN, FBN2, LOXL2, COL8A2, COL10A1, DPT, HAPLN1, HAPLN4, LGALS1, COL15A1, LAMA2, MMP10, OMD, COL14A1, FBLN2, EPYC, MFAP4, MFAP5	242	235	22680	13.95815017	3.10E-26	1.55E-26	1.62E-25
GOTERM_CC_DIRECT	GO:0005576~extracellular region	82	32.8	6.94E-28	ASPN, LTBP4, NELL1, MMP9, ARSI, IGFBP6, TGFB3, BCAN, POSTN, IGHM, CHAD, NOV, OGN, GPC3, CILP2, APOD, SEMA3D, SEMA3C, SEMA3B, LOXL4, LOX, HTRA3, TPSB2, LOXL2, IHH, COL10A1, MATN3, MFI2, CPXM2, SERPING1, DHRS7C, PCOLCE2, MMP10, CEMIP, WFDC1, EPYC, MFAP4, MFAP5, MIA, WNT16, ADAMTSL1, LUM, COL2A1, DCN, OSTN, MDK, FAM19A5, ITGBL1, C1QTNF9, COL9A1, COL9A2, LECT1, IL17B, COMP, ACAN, TNFRSF19, PTN, FBN2, ANGPTL2, COL8A2, BGLAP2, DPT, HAPLN1, BGLAP, HAPLN4, VSTM4, LGALS1, COL15A1, IGF2, CLEC11A, LAMA2, DKK3, NBL1, OMD, COL14A1, NPY, FBLN2, CLEC3A, CMA1, MEGF6, C1S1, PRSS23	237	1753	19662	3.880710825	1.72E-25	8.61E-26	9.01E-25
UP_SEQ_FEATURE	signal peptide	104	41.6	5.57E-24	ASPN, HFE2, MMP9, LTBP4, NELL1, TGFB3, POSTN, CHAD, NOV, OGN, APOD, HTRA3, LOX, TPSB2, 1810041L15RIK, COL10A1, MATN3, MFI2, IL11RA1, SERPING1, DHRS7C, PCOLCE2, PODXL2, CD300LG, WFDC1, EFNA5, MFAP4, MFAP5, FXYD1, FRAS1, FXYD3, ADAMTSL1, ITGB3, MDK, FXYD6, ITGBL1, IL17B, ITGAX, ANGPTL2, CSF1R, IGH, ITGA1, LAMA2, CD55, OMD, COL14A1, NPY, NTRK2, ARSB, IGFBP6, ARSI, BCAN, LRRC15, DDR2, ART1, GPC3, SEMA3D, SEMA3C, SEMA3B, LOXL4, LOXL2, RAMP1, IHH, CPXM2, NFAM1, MMP10, CEMIP, FKBP14, EPYC, CASQ1, MIA, WNT16, FKBP7, LUM, COL2A1, DCN, OSTN, C1QTNF9, COL9A1, COL9A2, COMP, ACAN, PTN, TNFRSF19, FBN2, COL8A2, BGLAP2, DPT, PTPRD, HAPLN4, BGLAP, MPZ, COL15A1, IGF2, CLEC11A, DKK3, NBL1, FBLN2, CLEC3A, CMA1, 3110079O15RIK, MEGF6, PRSS23, C1S1	223	3124	18012	2.688929336	5.79E-21	5.79E-21	8.84E-21

UP_KEYWORDS	Glycoprotein	108	43.2	5.62E-24	ASPN, HFE2, MMP9, LTBP4, NELL1, TGFB3, POSTN, IGHM, CHAD, NOV, OGN, APOD, ELOVL6, LOX, TPSB2, 1810041L15RIK, MATN3, MFI2, IL11RA1, SERPING1, PCOLCE2, PODXL2, SSTR2, CHPF, SLC37A2, CD300LG, EFNA5, MFAP4, PMP22, MFAP5, FRAS1, ADAMTSL1, ITGB3, SYPL2, ITGBL1, SERINC5, IL17B, ITGAX, ANGPTL2, CSF1R, VSTM4, ITGA1, CACNA1S, LAMA2, OMD, CD55, COL14A1, WSCD2, NTRK2, SCARA5, ARSB, SLC20A2, TRPV2, ARSI, ANO1, IGFBP6, BCAN, LRRC15, DDR2, ART1, GPC3, CILP2, XYLT1, SEMA3D, SEMA3C, SEMA3B, LOXL4, PIEZO1, LOXL2, ANO6, IHH, CPXM2, SEL1L3, NFAM1, CACNG1, CEMIP, CYBRD1, FKBP14, SGCD, EPYC, CASQ1, AOC3, WNT16, FKBP7, LUM, COL2A1, DCN, C1QTNF9, ITM2A, COL9A2, LECT1, COMP, ACAN, TNFRSF19, FBN2, HAPLN1, PTPRD, HAPLN4, MPZ, COL15A1, CLEC11A, DKK3, SLC17A8, FBLN2, CMA1, PRSS23, C1S1, MEGF6	242	3815	22680	2.653120024	1.39E-21	4.64E-22	7.29E-21
UP_KEYWORDS	Disulfide bond	95	38	1.08E-22	ASPN, ARSB, BACH2, HFE2, MMP9, LTBP4, NELL1, IGFBP6, TGFB3, BCAN, POSTN, IGHM, DDR2, ART1, CHAD, NOV, OGN, CILP2, APOD, XYLT1, SEMA3D, SEMA3C, SEMA3B, LOXL4, LOX, TPSB2, LOXL2, PIEZO1, RAMP1, MATN3, MFI2, IL11RA1, CPXM2, SERPING1, NFAM1, PCOLCE2, MMP10, SSTR2, PODXL2, CD300LG, SGCD, WFDC1, EFNA5, EPYC, MFAP5, AOC3, MIA, WNT16, ADAMTSL1, LUM, COL2A1, ITGB3, DCN, MDK, ITM2A, COL9A1, COL9A2, LECT1, IL17B, ITGAX, COMP, ACAN, TNFRSF19, PTN, FBN2, ANGPTL2, BGLAP2, CSF1R, DPT, HAPLN1, OBSCN, BGLAP, PTPRD, HAPLN4, VSTM4, MPZ, COL15A1, ITGA1, IGF2, CACNA1S, CLEC11A, LAMA2, NBL1, DKK3, OMD, CD55, COL14A1, FBLN2, CLEC3A, NTRK2, CMA1, MEGF6, PRSS23, C1S1, SCARA5	242	3124	22680	2.849969842	2.67E-20	6.67E-21	1.40E-19
UP_KEYWORDS	Signal	112	44.8	3.71E-20	ASPN, HFE2, MMP9, LTBP4, NELL1, TGFB3, POSTN, CHAD, OLFML1, NOV, OGN, APOD, HTRA3, LOX, TPSB2, 1810041L15RIK, COL10A1, MATN3, MFI2, IL11RA1, SERPING1, DHRS7C, PCOLCE2, PODXL2, CD300LG, WFDC1, EFNA5, MFAP4, MFAP5, FXYD1, FRAS1, FXYD3, ADAMTSL1, ITGB3, MDK, FXYD6, ABI3BP, ITGBL1, IL17B, ITGAX, NDRG4, ANGPTL2, CSF1R, IGH, VSTM4, ITGA1, LAMA2, OMD, CD55, COL14A1, NPY, NTRK2, ARSB, ARSI, IGFBP6, BCAN, LRRC15, DDR2, ART1, GPC3, CILP2, SEMA3D, SEMA3C, SEMA3B, LOXL4, LOXL2, RAMP1, IHH, CPXM2, NFAM1, SIGLEC15, MMP10, CEMIP, FKBP14, EPYC, CASQ1, MIA, WNT16, FKBP7, FHL1, LUM, COL2A1, DCN, OSTN, C1QTNF9, COL9A1, COL9A2, COL9A3, COMP, ACAN, PTN, TNFRSF19, FBN2, COL8A2, BGLAP2, DPT, BGLAP, PTPRD, HAPLN4, MPZ, COL15A1, IGF2, CLEC11A, DKK3, NBL1, FBLN2, CLEC3A, CMA1, 3110079O15RIK, PRSS23, C1S1, MEGF6	242	4543	22680	2.310484025	9.20E-18	1.84E-18	4.81E-17
UP_SEQ_FEATURE	disulfide bond	82	32.8	1.74E-17	ASPN, ARSB, LTBP4, NELL1, MMP9, IGFBP6, TGFB3, BCAN, POSTN, IGHM, DDR2, ART1, CHAD, NOV, OGN, APOD, SEMA3D, SEMA3C, SEMA3B, LOXL4, TPSB2, LOXL2, RAMP1, MATN3, MFI2, IL11RA1, CPXM2, SERPING1, NFAM1, PCOLCE2, MMP10, SSTR2, CD300LG, SGCD, WFDC1, EFNA5, EPYC, AOC3, MIA, ADAMTSL1, LUM, ITGB3, DCN, MDK, COL9A1, COL9A2, LECT1, IL17B, ITGAX, COMP, ACAN, TNFRSF19, PTN, FBN2, ANGPTL2, BGLAP2, CSF1R, DPT, HAPLN1, IGH, OBSCN, BGLAP, PTPRD, HAPLN4, MPZ, COL15A1, ITGA1, IGF2, CLEC11A, LAMA2, DKK3, NBL1, OMD, CD55, FBLN2, CLEC3A, NTRK2, CMA1, MEGF6, C1S1, PRSS23, SCARA5	223	2510	18012	2.63874368	1.81E-14	9.06E-15	2.77E-14

GOTERM_CC_DIRECT	GO:0031012~extracellular matrix	28	11.2	2.00E-16	ASPN, FRAS1, ADAMTSL1, LUM, MMP9, LTBP4, TGFB3, COL2A1, POSTN, DCN, NOV, OGN, COMP, FBN2, TPSB2, LOXL2, COL8A2, DPT, HAPLN1, LGALS1, COL15A1, CPXM2, MMP10, OMD, COL14A1, FBLN2, CMA1, MFAP4	237	294	19662	7.901145268	5.51E-14	1.83E-14	2.89E-13
UP_SEQ_FEATURE	glycosylation site:N-linked (GlcNAc...)	95	38	1.00E-14	ASPN, ARSB, HFE2, SLC20A2, MMP9, LTBP4, NELL1, TRPV2, ARSI, ANO1, TGFB3, BCAN, POSTN, LRRC15, IGHM, DDR2, ART1, NOV, OGN, GPC3, APOD, XYLT1, SEMA3D, SEMA3C, SEMA3B, LOXL4, LOX, TPSB2, LOXL2, ANO6, 1810041L15RIK, IHH, MATN3, MFI2, IL11RA1, CPXM2, SERPING1, SEL1L3, NFAM1, CACNG1, PCOLCE2, SSTR2, PODXL2, CHPF, SLC37A2, CEMIP, CYBRD1, FKBP14, SGCD, EFNA5, EPYC, MFAP4, PMP22, CASQ1, MFAP5, AOC3, FRAS1, WNT16, ADAMTSL1, FKBP7, ITGB3, DCN, SYPL2, ITGBL1, ITM2A, SERINC5, LECT1, IL17B, ITGAX, COMP, ACAN, TNFRSF19, FBN2, ANGPTL2, CSF1R, HAPLN1, IGH, PTPRD, HAPLN4, COL15A1, ITGA1, CACNA1S, LAMA2, SLC17A8, DKK3, OMD, CD55, COL14A1, FBLN2, NTRK2, CMA1, MEGF6, PRSS23, C1S1, SCARA5	223	3563	18012	2.153599086	1.04E-11	3.46E-12	1.59E-11
GOTERM_CC_DIRECT	GO:0005615~extracellular space	51	20.4	3.01E-11	PRKAG3, HFE2, MMP9, LTBP4, NELL1, IGFBP6, TGFB3, BCAN, POSTN, IGHM, OGN, GPC3, APOD, SEMA3D, SEMA3C, SEMA3B, LOXL4, LOX, TPSB2, LOXL2, RAMP1, IHH, ACTA2, MFI2, CPXM2, SERPING1, WFDC1, EPYC, LCP1, AOC3, WNT16, LUM, COL2A1, DCN, OSTN, ABI3BP, IL17B, COMP, ENO3, PTN, BGLAP2, DPT, BGLAP, LGALS1, COL15A1, IGF2, CLEC11A, DKK3, OMD, COL14A1, NPY	237	1504	19662	2.813206975	7.47E-09	1.49E-09	3.91E-08
Annotation Cluster 2	Enrichment Score: 5.529520942027068											
Category	Term	Count	%	PValue	Genes	List Total	Pop Hits	Pop Total	Fold Enrichment	Bonferroni	Benjamini	FDR
INTERPRO	IPR008160:Collagen triple helix repeat	10	4	2.14E-07	COL9A1, COL9A2, COL9A3, COL14A1, COL15A1, COL2A1, COL8A2, SCARA5, COL10A1, C1QTNF9	237	76	20594	11.43348879	1.13E-04	1.13E-04	3.10E-04
UP_KEYWORDS	Collagen	9	3.6	3.44E-06	COL9A1, COL9A2, COL9A3, COL14A1, COL15A1, COL2A1, COL8A2, COL10A1, C1QTNF9	242	85	22680	9.92318911	8.54E-04	7.76E-05	0.004467
UP_KEYWORDS	Hydroxylation	9	3.6	4.47E-06	COL9A1, COL9A2, COL14A1, COL15A1, COL2A1, C1S1, COL8A2, COL10A1, C1QTNF9	242	88	22680	9.584898573	0.001109	9.25E-05	0.005806
GOTERM_CC_DIRECT	GO:0005581~collagen trimer	9	3.6	6.96E-06	COL9A1, COL9A2, COL14A1, COL15A1, COL2A1, LOX, COL8A2, COL10A1, C1QTNF9	237	83	19662	8.995882263	0.001723	1.92E-04	0.009024
KEGG_PATHWAY	mmu04974:Protein digestion and absorption	9	3.6	9.82E-06	FXYD2, COL9A1, COL9A2, COL9A3, COL14A1, COL15A1, COL2A1, ATP1A2, COL10A1	94	88	7691	8.367867505	0.00154	0.00154	0.011817
Annotation Cluster 3	Enrichment Score: 5.055628936121134											
Category	Term	Count	%	PValue	Genes	List Total	Pop Hits	Pop Total	Fold Enrichment	Bonferroni	Benjamini	FDR
GOTERM_CC_DIRECT	GO:0033017~sarcoplasmic reticulum membrane	7	2.8	2.37E-06	MRLN, JPH2, JSRP1, RYR1, DHRS7C, CASQ1, ART1	237	33	19662	17.59800537	5.87E-04	8.39E-05	0.003074
GOTERM_CC_DIRECT	GO:0016529~sarcoplasmic reticulum	8	3.2	5.87E-06	MRLN, JPH2, JSRP1, RYR1, ITPR3, CACNA1S, CASQ1, ART1	237	58	19662	11.44303797	0.001454	1.82E-04	0.00761
UP_KEYWORDS	Sarcoplasmic reticulum	6	2.4	4.90E-05	MRLN, JPH2, JSRP1, RYR1, CASQ1, ART1	242	38	22680	14.79773815	0.012078	9.34E-04	0.063553
Annotation Cluster 4	Enrichment Score: 3.532184728248969											
Category	Term	Count	%	PValue	Genes	List Total	Pop Hits	Pop Total	Fold Enrichment	Bonferroni	Benjamini	FDR
KEGG_PATHWAY	mmu05410:Hypertrophic cardiomyopathy (HCM)	8	3.2	4.30E-05	PRKAG3, DES, ITGA1, TGFB3, SGCD, ITGB3, CACNG1, CACNA1S	94	79	7691	8.285483437	0.006725	0.003368	0.051719
KEGG_PATHWAY	mmu05414:Dilated cardiomyopathy	7	2.8	4.75E-04	DES, ITGA1, TGFB3, SGCD, ITGB3, CACNG1, CACNA1S	94	83	7691	6.900410151	0.071838	0.024543	0.569869
KEGG_PATHWAY	mmu05412:Arrhythmogenic right ventricular cardiomyopathy (ARVC)	6	2.4	0.00124	DES, ITGA1, SGCD, ITGB3, CACNG1, CACNA1S	94	67	7691	7.327087964	0.177116	0.038238	1.483323
Annotation Cluster 5	Enrichment Score: 3.2139012078899967											
Category	Term	Count	%	PValue	Genes	List Total	Pop Hits	Pop Total	Fold Enrichment	Bonferroni	Benjamini	FDR

UP_KEYWORDS	Immunoglobulin domain	22	8.8	5.32E-08	HAPLN1, IGH, OBSCN, PTPRD, HAPLN4, VSTM4, ADAMTSL1, MPZ, BCAN, IL11RA1, NFAM1, IGHM, MYOT, CILP2, MYOM3, NTRK2, CD300LG, ACAN, SEMA3D, SEMA3C, SEMA3B, CSF1R	242	481	22680	4.286524287	1.32E-05	1.65E-06	6.90E-05
INTERPRO	IPR003599:Immunoglobulin subtype	22	8.8	6.12E-07	HAPLN1, IGH, OBSCN, PTPRD, HAPLN4, ADAMTSL1, MPZ, MYBPC1, BCAN, IL11RA1, IGHM, SIGLEC15, MYOT, CILP2, MYOM2, MYOM3, NTRK2, CD300LG, ACAN, SEMA3C, SEMA3B, CSF1R	237	518	20594	3.690500627	3.23E-04	1.08E-04	8.89E-04
SMART	SM00409:IG	22	8.8	3.15E-05	HAPLN1, IGH, OBSCN, PTPRD, HAPLN4, ADAMTSL1, MPZ, MYBPC1, BCAN, IL11RA1, IGHM, SIGLEC15, MYOT, CILP2, MYOM2, MYOM3, NTRK2, CD300LG, ACAN, SEMA3C, SEMA3B, CSF1R	158	518	10425	2.802282391	0.004369	0.004369	0.037113
INTERPRO	IPR003598:Immunoglobulin subtype 2	12	4.8	1.16E-04	OBSCN, HAPLN4, PTPRD, ADAMTSL1, CILP2, MYOM2, MYOM3, MYBPC1, NTRK2, SEMA3C, MYOT, CSF1R	237	242	20594	4.308818914	0.059315	0.007614	0.167907
INTERPRO	IPR013098:Immunoglobulin I-set	9	3.6	3.00E-04	OBSCN, PTPRD, ADAMTSL1, MYOM2, MYOM3, MYBPC1, NTRK2, SEMA3C, MYOT	237	147	20594	5.320072333	0.146558	0.015723	0.434589
INTERPRO	IPR007110:Immunoglobulin-like domain	24	9.6	3.95E-04	HAPLN1, IGH, OBSCN, PTPRD, HAPLN4, VSTM4, ADAMTSL1, MPZ, MYBPC1, IL11RA1, BCAN, IGHM, SIGLEC15, MYOT, CILP2, MYOM2, MYOM3, NTRK2, CD300LG, ACAN, SEMA3D, SEMA3C, SEMA3B, CSF1R	237	920	20594	2.266813429	0.188298	0.018787	0.571703
INTERPRO	IPR013783:Immunoglobulin-like fold	26	10.4	8.68E-04	HAPLN1, IGH, OBSCN, VSTM4, PTPRD, HAPLN4, ADAMTSL1, MPZ, MYBPC1, IL11RA1, BCAN, IGHM, SIGLEC15, MYOT, ABI3BP, COL14A1, CILP2, MYOM2, MYOM3, NTRK2, CD300LG, ACAN, SEMA3D, SEMA3C, SEMA3B, CSF1R	237	1099	20594	2.055739203	0.367828	0.034662	1.252419
SMART	SM00408:IGc2	12	4.8	0.00107	OBSCN, HAPLN4, PTPRD, ADAMTSL1, CILP2, MYOM2, MYOM3, MYBPC1, NTRK2, SEMA3C, MYOT, CSF1R	158	242	10425	3.271785752	0.138023	0.029268	1.251286
UP_SEQ_FEATURE	domain:Ig-like C2-type 1	5	2	0.08065	PTPRD, MYOM3, NTRK2, MYOT, CSF1R	223	132	18012	3.059518956	1	0.813941	73.68823
UP_SEQ_FEATURE	domain:Ig-like C2-type 2	5	2	0.08239	PTPRD, MYOM3, NTRK2, MYOT, CSF1R	223	133	18012	3.036515054	1	0.814948	74.46757
INTERPRO	IPR013106:Immunoglobulin V-set	11	4.4	0.10574	OBSCN, HAPLN1, IGH, VSTM4, HAPLN4, MPZ, ACAN, CD300LG, BCAN, IGHM, SIGLEC15	237	554	20594	1.725342351	1	0.707505	80.24272
UP_SEQ_FEATURE	domain:Ig-like C2-type 3	3	1.2	0.29559	PTPRD, MYOM3, CSF1R	223	88	18012	2.753567061	1	0.994098	99.61654
Annotation Cluster 6	Enrichment Score: 2.7878458328833458											
Category	Term	Count	%	PValue	Genes	List Total	Pop Hits	Pop Total	Fold Enrichment	Bonferroni	Benjamini	FDR
INTERPRO	IPR009030:Insulin-like growth factor binding protein, N-terminal	12	4.8	3.11E-07	LAMA2, FRAS1, NOV, MATN3, FBLN2, NELL1, LTBP4, COMP, IGFBP6, FBN2, HTRA3, MEGF6	237	130	20594	8.021032132	1.64E-04	8.22E-05	4.52E-04
INTERPRO	IPR001881:EGF-like calcium-binding	10	4	1.51E-05	MATN3, FBLN2, NELL1, LTBP4, COMP, ACAN, BCAN, FBN2, MEGF6, C1S1	237	126	20594	6.896390061	0.007922	0.001986	0.021856
SMART	SM00181:EGF	12	4.8	8.86E-05	LAMA2, FRAS1, MATN3, FBLN2, NELL1, LTBP4, COMP, ACAN, BCAN, FBN2, MEGF6, ITGBL1	158	181	10425	4.374431778	0.012244	0.006141	0.104392
INTERPRO	IPR000742:Epidermal growth factor-like domain	12	4.8	9.63E-05	LAMA2, FRAS1, MATN3, FBLN2, NELL1, LTBP4, COMP, ACAN, BCAN, FBN2, MEGF6, ITGBL1	237	237	20594	4.399722267	0.0496	0.007241	0.13971
SMART	SM00179:EGF_CA	10	4	1.16E-04	MATN3, FBLN2, NELL1, LTBP4, COMP, ACAN, BCAN, FBN2, MEGF6, C1S1	158	126	10425	5.236588306	0.016024	0.00537	0.136857
INTERPRO	IPR018097:EGF-like calcium-binding, conserved site	8	3.2	1.27E-04	FBLN2, NELL1, LTBP4, COMP, ACAN, FBN2, MEGF6, C1S1	237	97	20594	7.166557919	0.064919	0.00743	0.184297
UP_KEYWORDS	EGF-like domain	11	4.4	1.51E-04	MATN3, FBLN2, NELL1, LTBP4, COMP, ACAN, BCAN, FBN2, MEGF6, C1S1, ITGBL1	242	224	22680	4.602272727	0.036655	0.002331	0.195177
INTERPRO	IPR000152:EGF-type aspartate/asparagine hydroxylation site	7	2.8	9.30E-04	FBLN2, NELL1, LTBP4, ACAN, FBN2, MEGF6, C1S1	237	98	20594	6.206751055	0.388073	0.034473	1.34071
INTERPRO	IPR026823:Complement Clr-like EGF domain	4	1.6	0.0017	MATN3, FBLN2, FBN2, MEGF6	237	21	20594	16.55133615	0.592022	0.054493	2.433808
INTERPRO	IPR013032:EGF-like, conserved site	9	3.6	0.00202	MATN3, FBLN2, NELL1, LTBP4, COMP, ACAN, BCAN, FBN2, MEGF6	237	197	20594	3.969800167	0.655661	0.060787	2.887473
UP_SEQ_FEATURE	domain:EGF-like 5; calcium-binding	4	1.6	0.00644	FBLN2, NELL1, LTBP4, FBN2	223	31	18012	10.42210328	0.99879	0.342834	9.747283
UP_SEQ_FEATURE	domain:EGF-like 4; calcium-binding	4	1.6	0.00704	FBLN2, LTBP4, FBN2, MEGF6	223	32	18012	10.09641256	0.999356	0.350932	10.61208
UP_SEQ_FEATURE	domain:EGF-like 10; calcium-binding	3	1.2	0.00773	FBLN2, LTBP4, FBN2	223	11	18012	22.02853649	0.999687	0.36132	11.59287

UP_SEQ_FEATURE	domain:EGF-like 11; calcium-binding	3	1.2	0.01248	FBLN2, LTBP4, FBN2	223	14	18012	17.30813581	0.999998	0.447776	18.08229
UP_SEQ_FEATURE	domain:EGF-like 9; calcium-binding	3	1.2	0.01248	FBLN2, LTBP4, FBN2	223	14	18012	17.30813581	0.999998	0.447776	18.08229
UP_SEQ_FEATURE	domain:EGF-like 8; calcium-binding	3	1.2	0.02483	FBLN2, LTBP4, FBN2	223	20	18012	12.11569507	1	0.558353	32.92007
UP_SEQ_FEATURE	domain:EGF-like 2; calcium-binding	4	1.6	0.02623	NELL1, LTBP4, COMP, MEGF6	223	52	18012	6.213176958	1	0.55645	34.42711
UP_SEQ_FEATURE	domain:EGF-like 6; calcium-binding	3	1.2	0.03228	FBLN2, NELL1, LTBP4	223	23	18012	10.53538702	1	0.602398	40.60874
UP_SEQ_FEATURE	domain:EGF-like 7; calcium-binding	3	1.2	0.03767	FBLN2, LTBP4, FBN2	223	25	18012	9.692556054	1	0.640818	45.64849
UP_SEQ_FEATURE	domain:EGF-like 3	4	1.6	0.04798	MATN3, NELL1, LTBP4, FBN2	223	66	18012	4.89523033	1	0.687162	54.19002
UP_SEQ_FEATURE	domain:EGF-like 1	5	2	0.04855	MATN3, NELL1, LTBP4, COMP, FBN2	223	111	18012	3.638346867	1	0.683399	54.62432
Annotation Cluster 7	Enrichment Score: 2.684204317706646											
Category	Term	Count	%	PValue	Genes	List Total	Pop Hits	Pop Total	Fold Enrichment	Bonferroni	Benjamini	FDR
GOTERM_MF_DIRECT	GO:0005201~extracellular matrix structural constituent	7	2.8	8.72E-06	HAPLN1, HAPLN4, COMP, ACAN, BCAN, COL2A1, FBN2	210	41	17446	14.18373984	0.003447	0.00115	0.012144
UP_SEQ_FEATURE	domain:Link 2	4	1.6	9.88E-05	HAPLN1, HAPLN4, ACAN, BCAN	223	8	18012	40.38565022	0.097678	0.025369	0.156807
UP_SEQ_FEATURE	domain:Link 1	4	1.6	9.88E-05	HAPLN1, HAPLN4, ACAN, BCAN	223	8	18012	40.38565022	0.097678	0.025369	0.156807
UP_KEYWORDS	Hyaluronic acid	4	1.6	1.35E-04	HAPLN1, HAPLN4, CEMIP, BCAN	242	10	22680	37.48760331	0.032825	0.002223	0.174457
GOTERM_MF_DIRECT	GO:0005540~hyaluronic acid binding	5	2	1.76E-04	HAPLN1, HAPLN4, CEMIP, ACAN, BCAN	210	24	17446	17.30753968	0.067402	0.011563	0.245125
INTERPRO	IPR000538:Link	4	1.6	4.93E-04	HAPLN1, HAPLN4, ACAN, BCAN	237	14	20594	24.82700422	0.229087	0.021448	0.712485
SMART	SM00445:LINK	4	1.6	0.00108	HAPLN1, HAPLN4, ACAN, BCAN	158	14	10425	18.8517179	0.139427	0.024716	1.264937
GOTERM_BP_DIRECT	GO:0001501~skeletal system development	7	2.8	0.0018	HAPLN1, HAPLN4, MMP9, BCAN, COL2A1, CACNA1S, IHH	217	107	18082	5.451311426	0.903619	0.164694	2.897459
INTERPRO	IPR016186:C-type lectin-like	7	2.8	0.00503	HAPLN1, HAPLN4, CLEC3A, ACAN, COL15A1, BCAN, CLEC11A	237	137	20594	4.439865718	0.930093	0.12456	7.051001
INTERPRO	IPR016187:C-type lectin fold	7	2.8	0.0066	HAPLN1, HAPLN4, CLEC3A, ACAN, COL15A1, BCAN, CLEC11A	237	145	20594	4.194907609	0.96962	0.153275	9.155647
UP_SEQ_FEATURE	domain:Ig-like V-type	6	2.4	0.01087	HAPLN1, MPZ, ACAN, CD300LG, BCAN, NFAM1	223	108	18012	4.487294469	0.999988	0.418048	15.93477
GOTERM_BP_DIRECT	GO:0007417~central nervous system development	5	2	0.02198	ARSB, HAPLN1, HAPLN4, ACAN, COL2A1	217	89	18082	4.681302749	1	0.523038	30.43267
INTERPRO	IPR003006:Immunoglobulin/major histocompatibility complex, conserved site	4	1.6	0.07415	IGH, ACAN, BCAN, IGHM	237	85	20594	4.089153636	1	0.595032	67.30408
INTERPRO	IPR013106:Immunoglobulin V-set	11	4.4	0.10574	OBSCN, HAPLN1, IGH, VSTM4, HAPLN4, MPZ, ACAN, CD300LG, BCAN, IGHM, SIGLEC15	237	554	20594	1.725342351	1	0.707505	80.24272
SMART	SM00406:IGv	8	3.2	0.45458	OBSCN, HAPLN1, IGH, HAPLN4, MPZ, ACAN, BCAN, IGHM	158	423	10425	1.247867852	1	0.945283	99.92101
Annotation Cluster 8	Enrichment Score: 2.6599602391047426											
Category	Term	Count	%	PValue	Genes	List Total	Pop Hits	Pop Total	Fold Enrichment	Bonferroni	Benjamini	FDR
UP_KEYWORDS	Developmental protein	24	9.6	3.16E-04	OBSCN, WNT16, DAB2IP, JPH2, TBX2, FHL1, ENC1, ANO1, PRRX2, INSC, OSTN, MDK, DKK3, LECT1, NTRK2, SEMA3D, RYR1, SEMA3C, SEMA3B, EFNA5, NDRG2, DCLK1, IHH, TWIST1	242	976	22680	2.304565777	0.075276	0.004338	0.408594
UP_KEYWORDS	Differentiation	17	6.8	0.00156	OBSCN, CPLX2, FHL1, NELL1, INSC, OSTN, MDK, LECT1, OCSTAMP, NTRK2, SEMA3D, SEMA3C, SEMA3B, EFNA5, NDRG2, DCLK1, TWIST1	242	646	22680	2.466289691	0.320889	0.015994	2.004144
GOTERM_BP_DIRECT	GO:0030154~cell differentiation	20	8	0.00264	OBSCN, CPLX2, FHL1, NELL1, INSC, OSTN, MDK, OCSTAMP, LECT1, ETS1, NTRK2, SEMA3D, SEMA3C, SEMA3B, EFNA5, NDRG2, PMP22, DCLK1, IHH, TWIST1	217	780	18082	2.136594588	0.967614	0.192956	4.219344
GOTERM_BP_DIRECT	GO:0007275~multicellular organism development	24	9.6	0.00288	OBSCN, WNT16, DAB2IP, JPH2, TBX2, FHL1, ENC1, ANO1, PRRX2, INSC, OSTN, MDK, DKK3, LECT1, NTRK2, SEMA3D, RYR1, SEMA3C, SEMA3B, EFNA5, NDRG2, DCLK1, IHH, TWIST1	217	1029	18082	1.943491287	0.976334	0.18778	4.596211
UP_KEYWORDS	Neurogenesis	9	3.6	0.00507	CPLX2, NTRK2, SEMA3D, SEMA3C, SEMA3B, EFNA5, NDRG2, INSC, DCLK1	242	247	22680	3.414862649	0.716398	0.044009	6.380236
GOTERM_BP_DIRECT	GO:0007399~nervous system development	12	4.8	0.0058	NBL1, CPLX2, ENC1, NTRK2, SEMA3D, SEMA3C, SEMA3B, EFNA5, NDRG2, INSC, DCLK1, ITM2A	217	377	18082	2.652324316	0.999476	0.270009	9.056531
Annotation Cluster 9	Enrichment Score: 2.5244989993128426											
Category	Term	Count	%	PValue	Genes	List Total	Pop Hits	Pop Total	Fold Enrichment	Bonferroni	Benjamini	FDR

UP_KEYWORDS	Proteoglycan	8	3.2	4.51E-07	COL9A2, OMD, GPC3, LUM, ACAN, BCAN, DCN, EPYC	242	45	22680	16.66115702	1.12E-04	1.24E-05	5.85E-04
GOTERM_MF_DIRECT	GO:0005518~collagen binding	9	3.6	4.87E-07	ASPN, LUM, COMP, ITGA1, DCN, LRRC15, DDR2, ABI3BP, PCOLCE2	210	59	17446	12.67263923	1.93E-04	9.64E-05	6.78E-04
INTERPRO	IPR000372:Leucine-rich repeat-containing N-terminal	7	2.8	3.11E-05	ASPN, OMD, LUM, NTRK2, DCN, EPYC, CHAD	237	53	20594	11.47663403	0.016288	0.003279	0.045123
SMART	SM00013:LRRNT	7	2.8	1.36E-04	ASPN, OMD, LUM, NTRK2, DCN, EPYC, CHAD	158	53	10425	8.71447337	0.018682	0.004704	0.159754
UP_SEQ_FEATURE	compositionally biased region:Cys-rich	9	3.6	2.72E-04	ASPN, OGN, SLC17A8, OMD, SERINC5, LTBP4, LUM, ITGB1BP2, DCN	223	135	18012	5.384753363	0.246452	0.055021	0.431095
GOTERM_BP_DIRECT	GO:0046426~negative regulation of JAK-STAT cascade	5	2	0.0017	ASPN, PTPRD, DCN, LRRC15, CHAD	217	43	18082	9.689208016	0.889867	0.16793	2.734547
BIOCARTA	m_slrpPathway:Small Leucine-rich Proteoglycan (SLRP) molecules	3	1.2	0.00331	LUM, DCN, EPYC	21	6	1289	30.69047619	0.147007	0.076424	3.111938
UP_SEQ_FEATURE	repeat:LRR 7	8	3.2	0.00349	ASPN, OGN, OMD, LUM, DCN, EPYC, LRRC15, CHAD	223	158	18012	4.089686099	0.973762	0.244249	5.406743
INTERPRO	IPR003591:Leucine-rich repeat, typical subtype	8	3.2	0.00413	ASPN, OGN, OMD, LUM, DCN, EPYC, LRRC15, CHAD	237	175	20594	3.972320675	0.887565	0.114329	5.829154
UP_SEQ_FEATURE	repeat:LRR 11	6	2.4	0.00465	ASPN, OMD, LUM, DCN, LRRC15, CHAD	223	88	18012	5.507134121	0.992163	0.292738	7.135961
GOTERM_MF_DIRECT	GO:0004860~protein kinase inhibitor activity	5	2	0.00482	ASPN, DCN, LRRC15, PKIA, CHAD	210	57	17446	7.287385129	0.852144	0.127626	6.501965
UP_SEQ_FEATURE	repeat:LRR 10	6	2.4	0.00897	ASPN, OMD, LUM, DCN, LRRC15, CHAD	223	103	18012	4.705124298	0.999915	0.38925	13.32759
UP_KEYWORDS	Leucine-rich repeat	9	3.6	0.00943	ASPN, OGN, OMD, LUM, NTRK2, DCN, EPYC, LRRC15, CHAD	242	275	22680	3.067167543	0.904554	0.070782	11.56498
UP_SEQ_FEATURE	repeat:LRR 6	8	3.2	0.00961	ASPN, OGN, OMD, LUM, DCN, EPYC, LRRC15, CHAD	223	191	18012	3.383091118	0.999956	0.394643	14.2103
INTERPRO	IPR001611:Leucine-rich repeat	9	3.6	0.01024	ASPN, OGN, OMD, LUM, NTRK2, DCN, EPYC, LRRC15, CHAD	237	259	20594	3.019500513	0.995642	0.218921	13.87641
UP_SEQ_FEATURE	repeat:LRR 5	8	3.2	0.01558	ASPN, OGN, OMD, LUM, DCN, EPYC, LRRC15, CHAD	223	210	18012	3.077001922	1	0.508307	22.0647
UP_SEQ_FEATURE	repeat:LRR 12	5	2	0.01644	ASPN, OMD, LUM, DCN, LRRC15	223	79	18012	5.112107623	1	0.512408	23.14108
SMART	SM00369:LRR_TYP	8	3.2	0.01657	ASPN, OGN, OMD, LUM, DCN, EPYC, LRRC15, CHAD	158	175	10425	3.016274864	0.902012	0.207284	17.87535
UP_SEQ_FEATURE	repeat:LRR 9	6	2.4	0.0182	ASPN, OMD, LUM, DCN, LRRC15, CHAD	223	123	18012	3.940063437	1	0.534216	25.29557
UP_SEQ_FEATURE	repeat:LRR 1	9	3.6	0.02065	ASPN, OGN, OMD, LUM, NTRK2, DCN, EPYC, LRRC15, CHAD	223	274	18012	2.653071913	1	0.526871	28.20562
UP_SEQ_FEATURE	repeat:LRR 2	9	3.6	0.02065	ASPN, OGN, OMD, LUM, NTRK2, DCN, EPYC, LRRC15, CHAD	223	274	18012	2.653071913	1	0.526871	28.20562
UP_SEQ_FEATURE	repeat:LRR 4	8	3.2	0.02299	ASPN, OGN, OMD, LUM, DCN, EPYC, LRRC15, CHAD	223	228	18012	2.834080717	1	0.541687	30.87653
UP_SEQ_FEATURE	repeat:LRR 8	6	2.4	0.02526	ASPN, OMD, LUM, DCN, LRRC15, CHAD	223	134	18012	3.616625393	1	0.553537	33.38889
GOTERM_BP_DIRECT	GO:0006469~negative regulation of protein kinase activity	5	2	0.02714	ASPN, DCN, LRRC15, PKIA, CHAD	217	95	18082	4.385641523	1	0.590763	36.18415
GOTERM_BP_DIRECT	GO:0019221~cytokine-mediated signaling pathway	6	2.4	0.03098	ASPN, IL11RA1, DCN, LRRC15, CSF1R, CHAD	217	146	18082	3.424405025	1	0.605106	40.1791
UP_SEQ_FEATURE	repeat:LRR 3	8	3.2	0.03768	ASPN, OGN, OMD, LUM, DCN, EPYC, LRRC15, CHAD	223	253	18012	2.554033216	1	0.631599	45.65697

Annotation Cluster 10	Enrichment Score: 2.518778563903055											
Category	Term	Count	%	PValue	Genes	List Total	Pop Hits	Pop Total	Fold Enrichment	Bonferroni	Benjamini	FDR
UP_KEYWORDS	Growth factor	8	3.2	4.25E-04	MIA, NOV, OGN, TGFB3, PTN, IGF2, MDK, CLEC11A	242	127	22680	5.903559576	0.100073	0.005534	0.550116
GOTERM_MF_DIRECT	GO:0008083~growth factor activity	8	3.2	0.00184	MIA, NOV, OGN, TGFB3, PTN, IGF2, MDK, CLEC11A	210	145	17446	4.583513957	0.516841	0.077643	2.525912
UP_KEYWORDS	Mitogen	4	1.6	0.00653	TGFB3, PTN, IGF2, MDK	242	36	22680	10.41322314	0.803187	0.05451	8.152521
GOTERM_BP_DIRECT	GO:0051781~positive regulation of cell division	4	1.6	0.01651	TGFB3, PTN, IGF2, MDK	217	45	18082	7.406861239	1	0.470519	23.79283

Annotation Cluster 11	Enrichment Score: 2.4421256482481906											
Category	Term	Count	%	PValue	Genes	List Total	Pop Hits	Pop Total	Fold Enrichment	Bonferroni	Benjamini	FDR
INTERPRO	IPR000272:Ion-transport regulator, FXYD motif	4	1.6	5.03E-05	FXYD1, FXYD2, FXYD3, FXYD6	237	7	20594	49.65400844	0.026194	0.004414	0.072921
GOTERM_MF_DIRECT	GO:0005216~ion channel activity	8	3.2	0.00445	FXYD1, FXYD2, FXYD3, TRPV2, RYR1, ITPR3, FXYD6, CACNA1S	210	170	17446	3.909467787	0.829056	0.127051	6.023592
GOTERM_BP_DIRECT	GO:2000649~regulation of sodium ion transmembrane transporter activity	3	1.2	0.01527	FXYD1, FXYD2, FXYD3	217	16	18082	15.62384793	1	0.464472	22.21056
GOTERM_MF_DIRECT	GO:0017080~sodium channel regulator activity	3	1.2	0.04989	FXYD1, FXYD2, FXYD3	210	30	17446	8.307619048	1	0.515096	50.97257

Annotation Cluster 12 Enrichment Score: 2.2908666201033108

Category	Term	Count	%	PValue	Genes	List Total	Pop Hits	Pop Total	Fold Enrichment	Bonferroni	Benjamini	FDR
UP_KEYWORDS	TPQ	4	1.6	1.84E-04	LOXL4, LOX, LOXL2, AOC3	242	11	22680	34.07963937	0.044511	0.002675	0.237928
GOTERM_MF_DIRECT	GO:0004720~protein-lysine 6-oxidase activity	3	1.2	4.25E-04	LOXL4, LOX, LOXL2	210	3	17446	83.07619048	0.154971	0.023768	0.590473
UP_SEQ_FEATURE	metal ion-binding site:Copper	4	1.6	7.53E-04	LOXL4, LOX, LOXL2, AOC3	223	15	18012	21.53901345	0.543299	0.12245	1.189461
UP_KEYWORDS	LTQ	3	1.2	0.0011	LOXL4, LOX, LOXL2	242	5	22680	56.23140496	0.239056	0.012341	1.419104
INTERPRO	IPR019828:Lysyl oxidase, conserved site	3	1.2	0.00128	LOXL4, LOX, LOXL2	237	5	20594	52.13670886	0.491025	0.044025	1.838921
INTERPRO	IPR001695:Lysyl oxidase	3	1.2	0.00128	LOXL4, LOX, LOXL2	237	5	20594	52.13670886	0.491025	0.044025	1.838921
GOTERM_MF_DIRECT	GO:0016641~oxidoreductase activity, acting on the CH-NH2 group of donors, oxygen as acceptor	3	1.2	0.00139	LOXL4, LOX, LOXL2	210	5	17446	49.84571429	0.424616	0.066757	1.92518
UP_SEQ_FEATURE	region of interest:Lysyl-oxidase like	3	1.2	0.00148	LOXL4, LOX, LOXL2	223	5	18012	48.46278027	0.784733	0.197008	2.317691
UP_SEQ_FEATURE	cross-link:Lysine tyrosylquinone (Lys-Tyr)	3	1.2	0.00148	LOXL4, LOX, LOXL2	223	5	18012	48.46278027	0.784733	0.197008	2.317691
UP_KEYWORDS	Copper	4	1.6	0.02382	LOXL4, LOX, LOXL2, AOC3	242	58	22680	6.46337988	0.997469	0.142134	26.86042
GOTERM_MF_DIRECT	GO:0005507~copper ion binding	4	1.6	0.03672	LOXL4, LOX, LOXL2, AOC3	210	61	17446	5.447619048	1	0.44713	40.61235
UP_KEYWORDS	Oxidoreductase	10	4	0.2401	ALDH1A3, FMO2, HR, CYBRD1, CYTB, LOXL4, LOX, LOXL2, DHRS7C, AOC3	242	639	22680	1.466651147	1	0.666545	97.1627
GOTERM_MF_DIRECT	GO:0016491~oxidoreductase activity	10	4	0.29974	ALDH1A3, FMO2, HR, CYBRD1, CYTB, LOXL4, LOX, LOXL2, DHRS7C, AOC3	210	604	17446	1.375433617	1	0.947107	99.30042
GOTERM_BP_DIRECT	GO:0055114~oxidation-reduction process	10	4	0.41881	ALDH1A3, FMO2, HR, CYBRD1, CYTB, LOXL4, LOX, LOXL2, DHRS7C, AOC3	217	676	18082	1.232650724	1	0.997583	99.9858

Annotation Cluster 13 Enrichment Score: 2.223003242280727

Category	Term	Count	%	PValue	Genes	List Total	Pop Hits	Pop Total	Fold Enrichment	Bonferroni	Benjamini	FDR
KEGG_PATHWAY	mmu04510:Focal adhesion	10	4	8.47E-04	LAMA2, COMP, ITGA1, MYLK2, MYLPF, COL2A1, ACTN2, ITGB3, ACTN3, CHAD	94	207	7691	3.952615891	0.124504	0.032695	1.014146
KEGG_PATHWAY	mmu04512:ECM-receptor interaction	6	2.4	0.00413	LAMA2, COMP, ITGA1, COL2A1, ITGB3, CHAD	94	88	7691	5.578578337	0.47758	0.077954	4.855653
KEGG_PATHWAY	mmu04151:PI3K-Akt signaling pathway	9	3.6	0.06133	LAMA2, COMP, ITGA1, COL2A1, PIK3AP1, EFNA5, ITGB3, CSF1R, CHAD	94	351	7691	2.097926896	0.999952	0.407272	53.31807

Annotation Cluster 14 Enrichment Score: 2.185567107337135

Category	Term	Count	%	PValue	Genes	List Total	Pop Hits	Pop Total	Fold Enrichment	Bonferroni	Benjamini	FDR
KEGG_PATHWAY	mmu04510:Focal adhesion	10	4	8.47E-04	LAMA2, COMP, ITGA1, MYLK2, MYLPF, COL2A1, ACTN2, ITGB3, ACTN3, CHAD	94	207	7691	3.952615891	0.124504	0.032695	1.014146
BIOCARTA	m_uCalpainPathway:uCalpain and friends in Cell spread	4	1.6	0.00129	ITGA1, ACTN2, ITGB3, ACTN3	21	15	1289	16.36825397	0.06019	0.06019	1.226658
KEGG_PATHWAY	mmu04810:Regulation of actin cytoskeleton	8	3.2	0.01435	ITGAX, SCIN, ITGA1, MYLK2, MYLPF, ACTN2, ITGB3, ACTN3	94	213	7691	3.073019678	0.896631	0.203035	15.96854
BIOCARTA	m_integrinPathway:Integrin Signaling Pathway	3	1.2	0.11529	ITGA1, ACTN2, ACTN3	21	38	1289	4.845864662	0.997204	0.859127	68.93302

Annotation Cluster 15 Enrichment Score: 2.0798828277452612

Category	Term	Count	%	PValue	Genes	List Total	Pop Hits	Pop Total	Fold Enrichment	Bonferroni	Benjamini	FDR
UP_SEQ_FEATURE	domain:TSP N-terminal	4	1.6	0.00155	COL9A1, COL14A1, NELL1, COL15A1	223	19	18012	17.0044843	0.800136	0.182304	2.428353
SMART	SM00210:TSPN	4	1.6	0.00418	COL9A1, COL14A1, NELL1, COL15A1	158	22	10425	11.99654776	0.441509	0.079848	4.818516
INTERPRO	IPR001791:Laminin G domain	5	2	0.0044	LAMA2, COL9A1, COL14A1, NELL1, COL15A1	237	58	20594	7.490906446	0.902302	0.115219	6.192063
INTERPRO	IPR013320:Concanavalin A-like lectin/glucanase, subgroup	8	3.2	0.01109	LAMA2, COL9A1, COL14A1, NELL1, LGALS1, COMP, RYR1, COL15A1	237	211	20594	3.294578759	0.997224	0.217519	14.93759
SMART	SM00282:LamG	3	1.2	0.12644	LAMA2, NELL1, COL15A1	158	41	10425	4.827878975	1	0.574327	79.66793

Annotation Cluster 16 Enrichment Score: 2.0448059311829545

Category	Term	Count	%	PValue	Genes	List Total	Pop Hits	Pop Total	Fold Enrichment	Bonferroni	Benjamini	FDR

UP_SEQ_FEATURE	metal ion-binding site:Calcium 2	5	2	0.00567	BGLAP, MMP9, PLCD4, BGLAP2, AOC3	223	58	18012	6.963043142	0.997284	0.325584	8.626307
UP_SEQ_FEATURE	metal ion-binding site:Calcium 3	4	1.6	0.00644	BGLAP, MMP9, PLCD4, BGLAP2	223	31	18012	10.42210328	0.99879	0.342834	9.747283
UP_SEQ_FEATURE	metal ion-binding site:Calcium 1	4	1.6	0.02012	BGLAP, MMP9, BGLAP2, AOC3	223	47	18012	6.87415323	1	0.556467	27.5829
Annotation Cluster 17	Enrichment Score: 1.7301803383202776											
Category	Term	Count	%	PValue	Genes	List Total	Pop Hits	Pop Total	Fold Enrichment	Bonferroni	Benjamini	FDR
GOTERM_BP_DIRECT	GO:0001558~regulation of cell growth	5	2	0.00508	NOV, FXYD2, IGFBP6, WFDC1, HTRA3	217	58	18082	7.183378357	0.998655	0.249826	7.973434
UP_SEQ_FEATURE	domain:IGFBP N-terminal	3	1.2	0.02032	NOV, IGFBP6, HTRA3	223	18	18012	13.46188341	1	0.546515	27.81894
INTERPRO	IPR000867:Insulin-like growth factor-binding protein, IGFBP	3	1.2	0.02169	NOV, IGFBP6, HTRA3	237	20	20594	13.03417722	0.999991	0.295938	27.25627
GOTERM_MF_DIRECT	GO:0005520~insulin-like growth factor binding	3	1.2	0.03064	NOV, IGFBP6, HTRA3	210	23	17446	10.83602484	0.999996	0.401539	35.16706
SMART	SM00121:IB	3	1.2	0.03259	NOV, IGFBP6, HTRA3	158	19	10425	10.41805463	0.99	0.280314	32.32258
Annotation Cluster 18	Enrichment Score: 1.6629218085994333											
Category	Term	Count	%	PValue	Genes	List Total	Pop Hits	Pop Total	Fold Enrichment	Bonferroni	Benjamini	FDR
UP_KEYWORDS	Chloride channel	4	1.6	0.01285	FXYD1, FXYD3, ANO1, ANO6	242	46	22680	8.14947898	0.959578	0.092647	15.4521
GOTERM_BP_DIRECT	GO:0006821~chloride transport	5	2	0.01546	FXYD1, FXYD3, WNK4, ANO1, ANO6	217	80	18082	5.207949309	1	0.458418	22.45802
GOTERM_CC_DIRECT	GO:0034707~chloride channel complex	4	1.6	0.01774	FXYD1, FXYD3, ANO1, ANO6	237	46	19662	7.214089158	0.98819	0.146601	20.72302
UP_KEYWORDS	Chloride	4	1.6	0.03452	FXYD1, FXYD3, ANO1, ANO6	242	67	22680	5.595164673	0.999835	0.187329	36.60494
GOTERM_MF_DIRECT	GO:0005254~chloride channel activity	4	1.6	0.03983	FXYD1, FXYD3, ANO1, ANO6	210	63	17446	5.27467876	1	0.461537	43.22496
Annotation Cluster 19	Enrichment Score: 1.6254041510533455											
Category	Term	Count	%	PValue	Genes	List Total	Pop Hits	Pop Total	Fold Enrichment	Bonferroni	Benjamini	FDR
UP_KEYWORDS	Ion transport	19	7.6	1.20E-04	ASPN, FXYD1, FXYD2, FXYD3, SLC20A2, TRPV2, MFI2, ANO1, ATP1A2, ITPR3, CACNG1, FXYD6, CACNA1S, SLC17A8, CACNA1G, RYR1, PIEZO1, SCARA5, ANO6	242	619	22680	2.876673921	0.029293	0.002121	0.15542
UP_KEYWORDS	Calcium channel	6	2.4	5.14E-04	TRPV2, RYR1, CACNA1G, ITPR3, CACNG1, CACNA1S	242	62	22680	9.069581445	0.119617	0.00635	0.664288
GOTERM_BP_DIRECT	GO:0070588~calcium ion transmembrane transport	7	2.8	6.50E-04	TRPV2, RYR1, CACNA1G, ITPR3, CACNG1, CACNA1S, ANO6	217	88	18082	6.62829912	0.570557	0.089643	1.056728
GOTERM_BP_DIRECT	GO:0006811~ion transport	18	7.2	6.73E-04	FXYD1, FXYD2, FXYD3, SLC20A2, TRPV2, MFI2, ANO1, ATP1A2, ITPR3, CACNG1, FXYD6, CACNA1S, SLC17A8, WNK4, RYR1, PIEZO1, SCARA5, ANO6	217	584	18082	2.568303769	0.583134	0.08378	1.093682
UP_KEYWORDS	Ion channel	12	4.8	9.92E-04	FXYD1, FXYD3, TRPV2, ANO1, RYR1, CACNA1G, ITPR3, CACNG1, PIEZO1, FXYD6, CACNA1S, ANO6	242	336	22680	3.347107438	0.218143	0.01165	1.279175
UP_KEYWORDS	Calcium transport	6	2.4	0.00316	TRPV2, RYR1, CACNA1G, ITPR3, CACNG1, CACNA1S	242	93	22680	6.04638763	0.544157	0.029764	4.026721
GOTERM_MF_DIRECT	GO:0005216~ion channel activity	8	3.2	0.00445	FXYD1, FXYD2, FXYD3, TRPV2, RYR1, ITPR3, FXYD6, CACNA1S	210	170	17446	3.909467787	0.829056	0.127051	6.023592
GOTERM_MF_DIRECT	GO:0005245~voltage-gated calcium channel activity	4	1.6	0.01381	RYR1, CACNA1G, CACNG1, CACNA1S	210	42	17446	7.912018141	0.995934	0.251539	17.60252
GOTERM_MF_DIRECT	GO:0005262~calcium channel activity	5	2	0.01982	TRPV2, RYR1, ITPR3, CACNG1, CACNA1S	210	86	17446	4.830011074	0.99964	0.314452	24.3342
KEGG_PATHWAY	mmu04020:Calcium signaling pathway	7	2.8	0.02153	PTK2B, RYR1, CACNA1G, MYLK2, PLCD4, ITPR3, CACNA1S	94	180	7691	3.181855792	0.967172	0.231109	23.04208
GOTERM_BP_DIRECT	GO:0006816~calcium ion transport	6	2.4	0.02721	TRPV2, RYR1, ITPR3, CACNG1, CACNA1S, RAMP1	217	141	18082	3.545837827	1	0.582768	36.2645
GOTERM_CC_DIRECT	GO:0005891~voltage-gated calcium channel complex	3	1.2	0.03329	CACNA1G, CACNG1, CACNA1S	237	24	19662	10.37025316	0.999774	0.237275	35.55012
INTERPRO	IPR005821:Ion transport domain	5	2	0.03475	TRPV2, RYR1, CACNA1G, ITPR3, CACNA1S	237	107	20594	4.060491344	1	0.396338	40.14504
KEGG_PATHWAY	mmu04921:Oxytocin signaling pathway	6	2.4	0.03488	PRKAG3, RYR1, MYLK2, ITPR3, CACNG1, CACNA1S	94	150	7691	3.272765957	0.996204	0.310358	34.77257
KEGG_PATHWAY	mmu04270:Vascular smooth muscle contraction	5	2	0.06745	ACTA2, MYLK2, ITPR3, CACNA1S, RAMP1	94	127	7691	3.221226336	0.999983	0.406727	56.85154
UP_KEYWORDS	Transport	27	10.8	0.11029	ASPN, FXYD1, FXYD2, CPLX2, FXYD3, SLC20A2, TRPV2, ANO1, CYTB, FXYD6, APOD, APOBR, CYGB, PIEZO1, ANO6, RAMP1, MFI2, ATP1A2, ITPR3, CACNG1, CACNA1S, SLC17A8, SLC37A2, CACNA1G, CYBRD1, RYR1, SCARA5	242	1901	22680	1.331095856	1	0.433482	78.04485
KEGG_PATHWAY	mmu04713:Circadian entrainment	4	1.6	0.11507	RYR1, CACNA1G, PRKG2, ITPR3	94	98	7691	3.339557099	1	0.522006	77.03713

UP_KEYWORDS	Voltage-gated channel	4	1.6	0.17636	CACNA1G, CACNG1, CACNA1S, ANO6	242	136	22680	2.75644142	1	0.557604	91.93268
GOTERM_BP_DIRECT	GO:0006810~transport	26	10.4	0.26106	FXYD1, FXYD2, CPLX2, FXYD3, SLC20A2, TRPV2, ANO1, CYTB, SYPL2, FXYD6, APOD, APOBR, CYGB, PIEZO1, ANO6, RAMP1, MFI2, ATP1A2, ITPR3, CACNG1, CACNA1S, SLC17A8, SLC37A2, CYBRD1, RYR1, SCARA5	217	1822	18082	1.18908173	1	0.981122	99.28411
KEGG_PATHWAY	mmu04925:Aldosterone synthesis and secretion	3	1.2	0.27906	CACNA1G, ITPR3, CACNA1S	94	86	7691	2.854156358	1	0.759963	98.05156
KEGG_PATHWAY	mmu04010:MAPK signaling pathway	5	2	0.36074	NTRK2, TGFB3, CACNA1G, CACNG1, CACNA1S	94	251	7691	1.629863525	1	0.812245	99.54172
GOTERM_BP_DIRECT	GO:0055085~transmembrane transport	6	2.4	0.43956	SLC17A8, TRPV2, SLC37A2, RYR1, ITPR3, CACNA1S	217	364	18082	1.373525092	1	0.998201	99.99216
GOTERM_MF_DIRECT	GO:0005244~voltage-gated ion channel activity	3	1.2	0.4787	CACNG1, CACNA1S, ANO6	210	134	17446	1.859914712	1	0.986423	99.98853
GOTERM_BP_DIRECT	GO:0034765~regulation of ion transmembrane transport	3	1.2	0.48106	CACNG1, CACNA1S, ANO6	217	135	18082	1.85171531	1	0.998844	99.99777

Annotation Cluster 20 Enrichment Score: 1.5889607986913958

Category	Term	Count	%	PValue	Genes	List Total	Pop Hits	Pop Total	Fold Enrichment	Bonferroni	Benjamini	FDR
UP_KEYWORDS	Sushi	4	1.6	0.01362	CD55, ACAN, BCAN, C1S1	242	47	22680	7.97608581	0.966696	0.095218	16.30457
INTERPRO	IPR000436:Sushi/SCR/CCP	4	1.6	0.02639	CD55, ACAN, BCAN, C1S1	237	56	20594	6.206751055	0.999999	0.339901	32.16681
SMART	SM00032:CCP	4	1.6	0.04757	CD55, ACAN, BCAN, C1S1	158	54	10425	4.887482419	0.998857	0.36341	43.69156

Annotation Cluster 21 Enrichment Score: 1.5671690549136115

Category	Term	Count	%	PValue	Genes	List Total	Pop Hits	Pop Total	Fold Enrichment	Bonferroni	Benjamini	FDR
GOTERM_BP_DIRECT	GO:0030335~positive regulation of cell migration	9	3.6	0.00311	PTK2B, ETS1, CEMIP, SEMA3D, SEMA3C, SEMA3B, ITGB3, LRRC15, CSF1R	217	203	18082	3.694308869	0.982481	0.191736	4.956131
GOTERM_BP_DIRECT	GO:0050919~negative chemotaxis	4	1.6	0.00357	SEMA3D, SEMA3C, SEMA3B, ITGB3	217	26	18082	12.81956753	0.990381	0.207211	5.669588
GOTERM_MF_DIRECT	GO:0038191~neuropilin binding	3	1.2	0.00381	SEMA3D, SEMA3C, SEMA3B	210	8	17446	31.15357143	0.779782	0.128516	5.182709
GOTERM_MF_DIRECT	GO:0045499~chemorepellent activity	4	1.6	0.00401	SEMA3D, SEMA3C, SEMA3B, EFNA5	210	27	17446	12.30758377	0.796321	0.124185	5.442697
INTERPRO	IPR016201:Plexin-like fold	4	1.6	0.0148	SEMA3D, SEMA3C, SEMA3B, ITGB3	237	45	20594	7.723956868	0.999619	0.245125	19.45775
INTERPRO	IPR027231:Semaphorin	3	1.2	0.02169	SEMA3D, SEMA3C, SEMA3B	237	20	20594	13.03417722	0.999991	0.295938	27.25627
SMART	SM00423:PSI	4	1.6	0.02204	SEMA3D, SEMA3C, SEMA3B, ITGB3	158	40	10425	6.598101266	0.954845	0.227512	23.09615
GOTERM_MF_DIRECT	GO:0030215~semaphorin receptor binding	3	1.2	0.02583	SEMA3D, SEMA3C, SEMA3B	210	21	17446	11.86802721	0.999968	0.362715	30.54241
GOTERM_BP_DIRECT	GO:0048843~negative regulation of axon extension involved in axon guidance	3	1.2	0.03825	SEMA3D, SEMA3C, SEMA3B	217	26	18082	9.614675647	1	0.636978	47.0999
UP_SEQ_FEATURE	domain:Ig-like C2-type	5	2	0.03861	HAPLN4, SEMA3D, IL11RA1, SEMA3C, SEMA3B	223	103	18012	3.920936915	1	0.631699	46.48827
UP_SEQ_FEATURE	domain:Sema	3	1.2	0.04634	SEMA3D, SEMA3C, SEMA3B	223	28	18012	8.654067905	1	0.682603	52.92496
INTERPRO	IPR001627:Semaphorin/CD100 antigen	3	1.2	0.04892	SEMA3D, SEMA3C, SEMA3B	237	31	20594	8.40914659	1	0.475794	51.70072
GOTERM_BP_DIRECT	GO:0071526~semaphorin-plexin signaling pathway	3	1.2	0.06208	SEMA3D, SEMA3C, SEMA3B	217	34	18082	7.352399024	1	0.756119	64.87824
KEGG_PATHWAY	mmu04360:Axon guidance	5	2	0.07059	ABLIM3, SEMA3D, SEMA3C, SEMA3B, EFNA5	94	129	7691	3.171284842	0.99999	0.406906	58.56549
SMART	SM00630:Sema	3	1.2	0.07889	SEMA3D, SEMA3C, SEMA3B	158	31	10425	6.38525929	0.999989	0.469832	62.02895
GOTERM_BP_DIRECT	GO:0001755~neural crest cell migration	3	1.2	0.11609	SEMA3D, SEMA3C, SEMA3B	217	49	18082	5.101664629	1	0.885374	86.66213
INTERPRO	IPR015943:WD40/YVTN repeat-like-containing domain	5	2	0.54749	WDFY4, SEMA3D, SEMA3C, SEMA3B, KIF21B	237	340	20594	1.277860511	1	0.997684	99.99899

Annotation Cluster 22 Enrichment Score: 1.5650013598585362

Category	Term	Count	%	PValue	Genes	List Total	Pop Hits	Pop Total	Fold Enrichment	Bonferroni	Benjamini	FDR
UP_SEQ_FEATURE	repeat:II	3	1.2	0.02723	ITGB3, CACNA1S, ITGBL1	223	21	18012	11.53875721	1	0.559677	35.48822
UP_SEQ_FEATURE	repeat:III	3	1.2	0.02723	ITGB3, CACNA1S, ITGBL1	223	21	18012	11.53875721	1	0.559677	35.48822
UP_SEQ_FEATURE	repeat:I	3	1.2	0.02723	ITGB3, CACNA1S, ITGBL1	223	21	18012	11.53875721	1	0.559677	35.48822
UP_SEQ_FEATURE	repeat:IV	3	1.2	0.02723	ITGB3, CACNA1S, ITGBL1	223	21	18012	11.53875721	1	0.559677	35.48822

Annotation Cluster 23 Enrichment Score: 1.4782703401641644

Category	Term	Count	%	PValue	Genes	List Total	Pop Hits	Pop Total	Fold Enrichment	Bonferroni	Benjamini	FDR
UP_KEYWORDS	LIM domain	5	2	0.008	XIRP2, FHL1, ABLIM3, PDLIM3, LDB3	242	74	22680	6.332365423	0.863737	0.062272	9.902352
INTERPRO	IPR001781:Zinc finger, LIM-type	5	2	0.01035	XIRP2, FHL1, ABLIM3, PDLIM3, LDB3	237	74	20594	5.871250998	0.995877	0.212389	14.00779
SMART	SM00132:LIM	5	2	0.02524	XIRP2, FHL1, ABLIM3, PDLIM3, LDB3	158	74	10425	4.458176531	0.971392	0.239205	26.01507
UP_SEQ_FEATURE	domain:LIM zinc-binding 3	3	1.2	0.04048	FHL1, ABLIM3, LDB3	223	26	18012	9.319765436	1	0.640598	48.11836
UP_SEQ_FEATURE	domain:LIM zinc-binding 2	3	1.2	0.1263	FHL1, ABLIM3, LDB3	223	50	18012	4.846278027	1	0.914859	88.27997
UP_SEQ_FEATURE	domain:LIM zinc-binding 1	3	1.2	0.1263	FHL1, ABLIM3, LDB3	223	50	18012	4.846278027	1	0.914859	88.27997

Annotation Cluster 24 Enrichment Score: 1.4091949411937297

Category	Term	Count	%	PValue	Genes	List Total	Pop Hits	Pop Total	Fold Enrichment	Bonferroni	Benjamini	FDR
GOTERM_CC_DIRECT	GO:0031225~anchored component of membrane	7	2.8	0.00718	CD55, GPC3, HFE2, MFI2, BCAN, EFNA5, ART1	237	141	19662	4.118682108	0.832354	0.07797	8.920046
UP_KEYWORDS	GPI-anchor	6	2.4	0.01704	CD55, GPC3, HFE2, MFI2, EFNA5, ART1	242	140	22680	4.016528926	0.985896	0.108786	19.98359
UP_SEQ_FEATURE	propeptide:Removed in mature form	7	2.8	0.07479	CD55, GPC3, HFE2, ACTA2, MFI2, EFNA5, ART1	223	238	18012	2.375626484	1	0.801475	70.89591
GOTERM_CC_DIRECT	GO:0046658~anchored component of plasma membrane	3	1.2	0.07608	GPC3, MFI2, EFNA5	237	38	19662	6.549633578	1	0.420245	64.18252
UP_KEYWORDS	Lipoprotein	13	5.2	0.12933	FXYD1, WNT16, HFE2, MFI2, AKAP12, PRKG2, ART1, CD55, SSTR2, GPC3, APOBR, EFNA5, IHH	242	780	22680	1.561983471	1	0.458441	83.4174

Annotation Cluster 25 Enrichment Score: 1.3628111356529113

Category	Term	Count	%	PValue	Genes	List Total	Pop Hits	Pop Total	Fold Enrichment	Bonferroni	Benjamini	FDR
INTERPRO	IPR001715:Calponin homology domain	5	2	0.01133	TAGLN, ACTN2, SMTNL1, ACTN3, LCP1	237	76	20594	5.716744393	0.997568	0.213965	15.2456
INTERPRO	IPR002048:EF-hand domain	8	3.2	0.01466	FKBP7, RYR1, FKBP14, MYLPF, PLCD4, ACTN2, ACTN3, LCP1	237	223	20594	3.11729201	0.999588	0.25078	19.28449
SMART	SM00033:CH	5	2	0.01733	TAGLN, ACTN2, SMTNL1, ACTN3, LCP1	158	66	10425	4.998561565	0.911963	0.19821	18.61754
UP_SEQ_FEATURE	domain:EF-hand 2	7	2.8	0.02047	FKBP7, FKBP14, MYLPF, PLCD4, ACTN2, ACTN3, LCP1	223	173	18012	3.268202908	1	0.53624	27.99883
UP_SEQ_FEATURE	domain:EF-hand 1	7	2.8	0.02099	FKBP7, FKBP14, MYLPF, PLCD4, ACTN2, ACTN3, LCP1	223	174	18012	3.249420133	1	0.520624	28.59358
UP_SEQ_FEATURE	domain:CH 2	3	1.2	0.02723	ACTN2, ACTN3, LCP1	223	21	18012	11.53875721	1	0.559677	35.48822
UP_SEQ_FEATURE	domain:CH 1	3	1.2	0.02723	ACTN2, ACTN3, LCP1	223	21	18012	11.53875721	1	0.559677	35.48822
INTERPRO	IPR001589:Actinin-type, actin-binding, conserved site	3	1.2	0.02824	ACTN2, ACTN3, LCP1	237	23	20594	11.33406714	1	0.343093	34.01508
INTERPRO	IPR011992:EF-hand-like domain	8	3.2	0.03852	FKBP7, RYR1, FKBP14, MYLPF, PLCD4, ACTN2, ACTN3, LCP1	237	273	20594	2.546359407	1	0.420612	43.44625
UP_SEQ_FEATURE	calcium-binding region:2	4	1.6	0.16636	FKBP7, FKBP14, PLCD4, LCP1	223	114	18012	2.834080717	1	0.950393	94.43755
SMART	SM00054:EFh	5	2	0.17508	MYLPF, PLCD4, ACTN2, ACTN3, LCP1	158	145	10425	2.275207333	1	0.687516	89.64942
UP_SEQ_FEATURE	calcium-binding region:1	4	1.6	0.20343	FKBP7, FKBP14, PLCD4, LCP1	223	126	18012	2.564168268	1	0.972234	97.29887
INTERPRO	IPR018247:EF-Hand 1, calcium-binding site	4	1.6	0.32492	FKBP7, FKBP14, MYLPF, LCP1	237	175	20594	1.986160338	1	0.964783	99.66596

Annotation Cluster 26 Enrichment Score: 1.3496069740581744

Category	Term	Count	%	PValue	Genes	List Total	Pop Hits	Pop Total	Fold Enrichment	Bonferroni	Benjamini	FDR
INTERPRO	IPR001007:von Willebrand factor, type C	4	1.6	0.0115	FRAS1, NOV, NELL1, COL2A1	237	41	20594	8.477513636	0.997769	0.209287	15.44676
SMART	SM00214:VWC	4	1.6	0.01542	FRAS1, NOV, NELL1, COL2A1	158	35	10425	7.540687161	0.88461	0.213323	16.7293
GOTERM_BP_DIRECT	GO:0010468~regulation of gene expression	5	2	0.50426	NOV, NELL1, COL2A1, SCARA5, H19	217	308	18082	1.352714106	1	0.999146	99.99894

Annotation Cluster 27 Enrichment Score: 1.3415552380718572

Category	Term	Count	%	PValue	Genes	List Total	Pop Hits	Pop Total	Fold Enrichment	Bonferroni	Benjamini	FDR
SMART	SM00060:FN3	8	3.2	0.00838	OBSCN, PTPRD, COL14A1, MYOM2, MYOM3, MYBPC1, IL11RA1, ABI3BP	158	153	10425	3.44998759	0.689695	0.13608	9.444522
INTERPRO	IPR003961:Fibronectin, type III	8	3.2	0.01109	OBSCN, PTPRD, COL14A1, MYOM2, MYOM3, MYBPC1, IL11RA1, ABI3BP	237	211	20594	3.294578759	0.997224	0.217519	14.93759
UP_SEQ_FEATURE	domain:Fibronectin type-III 2	5	2	0.06741	OBSCN, PTPRD, COL14A1, MYOM3, IL11RA1	223	124	18012	3.256907276	1	0.786531	66.98328
UP_SEQ_FEATURE	domain:Fibronectin type-III 3	4	1.6	0.06751	OBSCN, PTPRD, COL14A1, MYOM3	223	76	18012	4.251121076	1	0.780059	67.03899
UP_SEQ_FEATURE	domain:Fibronectin type-III 1	5	2	0.069	OBSCN, PTPRD, COL14A1, MYOM3, IL11RA1	223	125	18012	3.230852018	1	0.78072	67.86477

UP_SEQ_FEATURE	domain:Fibronectin type-III 5	3	1.2	0.08696	PTPRD, COL14A1, MYOM3	223	40	18012	6.057847534	1	0.826593	76.41514
UP_SEQ_FEATURE	domain:Fibronectin type-III 4	3	1.2	0.16019	PTPRD, COL14A1, MYOM3	223	58	18012	4.177825885	1	0.946519	93.7469

Annotation Cluster 28 Enrichment Score: 1.2710177956796367

Category	Term	Count	%	PValue	Genes	List Total	Pop Hits	Pop Total	Fold Enrichment	Bonferroni	Benjamini	FDR
INTERPRO	IPR016186:C-type lectin-like	7	2.8	0.00503	HAPLN1, HAPLN4, CLEC3A, ACAN, COL15A1, BCAN, CLEC11A	237	137	20594	4.439865718	0.930093	0.12456	7.051001
INTERPRO	IPR016187:C-type lectin fold	7	2.8	0.0066	HAPLN1, HAPLN4, CLEC3A, ACAN, COL15A1, BCAN, CLEC11A	237	145	20594	4.194907609	0.96962	0.153275	9.155647
INTERPRO	IPR018378:C-type lectin, conserved site	4	1.6	0.02067	CLEC3A, ACAN, BCAN, CLEC11A	237	51	20594	6.81525606	0.999984	0.29147	26.14169
UP_KEYWORDS	Lectin	5	2	0.11379	LGALS1, CLEC3A, ACAN, BCAN, CLEC11A	242	173	22680	2.708641857	1	0.431791	79.14046
UP_SEQ_FEATURE	domain:C-type lectin	4	1.6	0.1261	CLEC3A, ACAN, BCAN, CLEC11A	223	100	18012	3.230852018	1	0.918173	88.23691
GOTERM_MF_DIRECT	GO:0030246~carbohydrate binding	6	2.4	0.14129	CILP2, LGALS1, CLEC3A, ACAN, BCAN, CLEC11A	210	229	17446	2.176668746	1	0.812808	88.01592
INTERPRO	IPR001304:C-type lectin	4	1.6	0.17034	CLEC3A, ACAN, BCAN, CLEC11A	237	124	20594	2.803048863	1	0.849855	93.34472
SMART	SM00034:CLECT	4	1.6	0.28697	CLEC3A, ACAN, BCAN, CLEC11A	158	124	10425	2.128419763	1	0.824708	98.14236

Annotation Cluster 29 Enrichment Score: 1.2205763283383946

Category	Term	Count	%	PValue	Genes	List Total	Pop Hits	Pop Total	Fold Enrichment	Bonferroni	Benjamini	FDR
INTERPRO	IPR001190:Speract/scavenger receptor	3	1.2	0.04066	LOXL4, LOXL2, SCARA5	237	28	20594	9.310126582	1	0.429903	45.24517
INTERPRO	IPR017448:Speract/scavenger receptor-related	3	1.2	0.04335	LOXL4, LOXL2, SCARA5	237	29	20594	8.989087735	1	0.442866	47.43048
SMART	SM00202:SR	3	1.2	0.06601	LOXL4, LOXL2, SCARA5	158	28	10425	7.069394213	0.999925	0.447474	55.27869
GOTERM_MF_DIRECT	GO:0005044~scavenger receptor activity	3	1.2	0.11272	LOXL4, LOXL2, SCARA5	210	48	17446	5.192261905	1	0.761908	81.09282

Annotation Cluster 30 Enrichment Score: 1.1704680796478408

Category	Term	Count	%	PValue	Genes	List Total	Pop Hits	Pop Total	Fold Enrichment	Bonferroni	Benjamini	FDR
UP_SEQ_FEATURE	domain:C1q	3	1.2	0.04938	COL8A2, COL10A1, C1QTNF9	223	29	18012	8.355651771	1	0.681743	55.25052
INTERPRO	IPR001073:Complement C1q protein	3	1.2	0.05179	COL8A2, COL10A1, C1QTNF9	237	32	20594	8.146360759	1	0.487561	53.7777
SMART	SM00110:C1Q	3	1.2	0.07022	COL8A2, COL10A1, C1QTNF9	158	29	10425	6.825621999	0.99996	0.448595	57.59667
INTERPRO	IPR008983:Tumour necrosis factor-like domain	3	1.2	0.11584	COL8A2, COL10A1, C1QTNF9	237	51	20594	5.111442045	1	0.734649	83.24688

Annotation Cluster 31 Enrichment Score: 1.1556933229856687

Category	Term	Count	%	PValue	Genes	List Total	Pop Hits	Pop Total	Fold Enrichment	Bonferroni	Benjamini	FDR
UP_KEYWORDS	Myosin	4	1.6	0.01442	MYO18B, MYH2, MYH11, MYLPF	242	48	22680	7.809917355	0.972742	0.097807	17.17721
INTERPRO	IPR027401:Myosin-like IQ motif-containing domain	3	1.2	0.01773	MYO18B, MYH2, MYH11	237	18	20594	14.48241913	0.999921	0.270115	22.86399
INTERPRO	IPR002928:Myosin tail	3	1.2	0.01967	MYO18B, MYH2, MYH11	237	19	20594	13.72018654	0.999972	0.287055	25.04329
UP_KEYWORDS	Motor protein	5	2	0.04789	MYO18B, MYH2, MYH11, MYLPF, KIF21B	242	128	22680	3.66089876	0.999995	0.241655	47.09931
INTERPRO	IPR001609:Myosin head, motor domain	3	1.2	0.07349	MYO18B, MYH2, MYH11	237	39	20594	6.684193444	1	0.599851	66.96337
INTERPRO	IPR000048:IQ motif, EF-hand binding site	4	1.6	0.0804	OBSCN, MYO18B, MYH2, MYH11	237	88	20594	3.949750671	1	0.617899	70.36578
SMART	SM00242:MYSc	3	1.2	0.11645	MYO18B, MYH2, MYH11	158	39	10425	5.075462512	1	0.577036	76.75266
GOTERM_MF_DIRECT	GO:0003774~motor activity	3	1.2	0.24436	MYO18B, MYH2, MYH11	210	79	17446	3.154792043	1	0.915044	97.98047
INTERPRO	IPR027417:P-loop containing nucleoside triphosphate hydrolase	6	2.4	0.98027	RERG, MYO18B, WSCD2, MYH2, MYH11, KIF21B	237	909	20594	0.573561154	1	1	100

Annotation Cluster 32 Enrichment Score: 1.146832515067825

Category	Term	Count	%	PValue	Genes	List Total	Pop Hits	Pop Total	Fold Enrichment	Bonferroni	Benjamini	FDR
INTERPRO	IPR002035:von Willebrand factor, type A	5	2	0.02718	MATN3, COL14A1, ITGAX, ITGA1, ITGB3	237	99	20594	4.388611857	1	0.340125	32.95857
UP_SEQ_FEATURE	domain:VWFA	4	1.6	0.02892	MATN3, ITGAX, ITGA1, ITGB3	223	54	18012	5.983059292	1	0.571603	37.24539
GOTERM_CC_DIRECT	GO:0008305~integrin complex	3	1.2	0.0359	ITGAX, ITGA1, ITGB3	237	25	19662	9.955443038	0.999885	0.246765	37.77491
SMART	SM00327:VWA	4	1.6	0.09144	MATN3, COL14A1, ITGAX, ITGA1	158	71	10425	3.71724015	0.999998	0.504194	67.69922
GOTERM_BP_DIRECT	GO:0007229~integrin-mediated signaling pathway	4	1.6	0.10023	ITGAX, PTK2B, ITGA1, ITGB3	217	93	18082	3.583965116	1	0.867032	82.17133

UP_KEYWORDS	Integrin	3	1.2	0.10901	ITGAX, ITGA1, ITGB3	242	53	22680	5.304849524	1	0.435883	77.63218
GOTERM_CC_DIRECT	GO:0009897~external side of plasma membrane	6	2.4	0.33265	CD55, ITGAX, ANO1, ITGA1, ITGB3, IGHM	237	317	19662	1.570259154	1	0.791374	99.47391

Annotation Cluster 33	Enrichment Score: 1.1270586139025867											
Category	Term	Count	%	PValue	Genes	List Total	Pop Hits	Pop Total	Fold Enrichment	Bonferroni	Benjamini	FDR
KEGG_PATHWAY	mmu04260:Cardiac muscle contraction	6	2.4	0.00231	FXYD2, COX7A1, CYTB, ATP1A2, CACNG1, CACNA1S	94	77	7691	6.375518099	0.304401	0.058703	2.744304
KEGG_PATHWAY	mmu04022:cGMP-PKG signaling pathway	6	2.4	0.04711	FXYD2, MYLK2, PRKG2, ATP1A2, ITPR3, CACNA1S	94	163	7691	3.011747814	0.999487	0.377159	44.0517
KEGG_PATHWAY	mmu04970:Salivary secretion	4	1.6	0.06603	FXYD2, PRKG2, ATP1A2, ITPR3	94	77	7691	4.250345399	0.999978	0.415068	56.05393
KEGG_PATHWAY	mmu04911:Insulin secretion	4	1.6	0.08562	FXYD2, ATP1A2, ITPR3, CACNA1S	94	86	7691	3.805541811	0.999999	0.443201	65.94996
KEGG_PATHWAY	mmu04024:cAMP signaling pathway	6	2.4	0.0898	FXYD1, FXYD2, SSTR2, NPY, ATP1A2, CACNA1S	94	197	7691	2.491953775	1	0.446162	67.7756
KEGG_PATHWAY	mmu04918:Thyroid hormone synthesis	3	1.2	0.20737	FXYD2, ATP1A2, ITPR3	94	70	7691	3.506534954	1	0.703643	93.90099
KEGG_PATHWAY	mmu04261:Adrenergic signaling in cardiomyocytes	4	1.6	0.24609	FXYD2, ATP1A2, CACNG1, CACNA1S	94	142	7691	2.304764759	1	0.728659	96.66214
KEGG_PATHWAY	mmu04972:Pancreatic secretion	3	1.2	0.34162	FXYD2, ATP1A2, ITPR3	94	100	7691	2.454574468	1	0.806126	99.34655

Annotation Cluster 34	Enrichment Score: 1.070554618211922											
Category	Term	Count	%	PValue	Genes	List Total	Pop Hits	Pop Total	Fold Enrichment	Bonferroni	Benjamini	FDR
UP_KEYWORDS	Immunity	10	4	0.02781	SERINC5, DAB2IP, CD55, PTK2B, ETS1, CD300LG, SERPING1, INPP5D, C1S1, CSF1R	242	401	22680	2.337132376	0.999082	0.160413	30.64234
GOTERM_BP_DIRECT	GO:0006958~complement activation, classical pathway	4	1.6	0.02799	CD55, SERPING1, IGHM, C1S1	217	55	18082	6.060159196	1	0.584415	37.09231
UP_KEYWORDS	Complement pathway	3	1.2	0.03315	CD55, SERPING1, C1S1	242	27	22680	10.41322314	0.999766	0.18448	35.43029
GOTERM_BP_DIRECT	GO:0002376~immune system process	10	4	0.04104	SERINC5, DAB2IP, CD55, PTK2B, ETS1, CD300LG, SERPING1, INPP5D, C1S1, CSF1R	217	383	18082	2.17564462	1	0.648941	49.54729
UP_KEYWORDS	Innate immunity	6	2.4	0.11564	SERINC5, DAB2IP, CD55, SERPING1, C1S1, CSF1R	242	241	22680	2.333253318	1	0.4313	79.69884
GOTERM_BP_DIRECT	GO:0045087~innate immune response	8	3.2	0.19989	SERINC5, DAB2IP, CD55, PTK2B, SERPING1, IGHM, C1S1, CSF1R	217	400	18082	1.666543779	1	0.9642	97.3772
KEGG_PATHWAY	mmu04610:Complement and coagulation cascades	3	1.2	0.23412	CD55, SERPING1, C1S1	94	76	7691	3.229703247	1	0.718876	95.96531
GOTERM_CC_DIRECT	GO:0072562~blood microparticle	3	1.2	0.47571	SERPING1, IGHM, C1S1	237	133	19662	1.871323879	1	0.898496	99.97701

Annotation Cluster 35	Enrichment Score: 0.8691355586444103											
Category	Term	Count	%	PValue	Genes	List Total	Pop Hits	Pop Total	Fold Enrichment	Bonferroni	Benjamini	FDR
INTERPRO	IPR001478:PDZ domain	5	2	0.10125	PDLIM3, LDB3, PDZRN4, HTRA3, SYNPO2L	237	154	20594	2.821250479	1	0.698569	78.75383
UP_SEQ_FEATURE	domain:PDZ	4	1.6	0.12884	PDLIM3, LDB3, HTRA3, SYNPO2L	223	101	18012	3.198863384	1	0.915694	88.8107
SMART	SM00228:PDZ	5	2	0.1893	PDLIM3, LDB3, PDZRN4, HTRA3, SYNPO2L	158	150	10425	2.199367089	1	0.688633	91.56635

Annotation Cluster 36	Enrichment Score: 0.8408435484782332											
Category	Term	Count	%	PValue	Genes	List Total	Pop Hits	Pop Total	Fold Enrichment	Bonferroni	Benjamini	FDR
UP_KEYWORDS	Sodium transport	4	1.6	0.1193	SLC17A8, FXYD2, SLC20A2, ATP1A2	242	113	22680	3.317487018	1	0.436084	80.76293
UP_KEYWORDS	Sodium	4	1.6	0.13587	SLC17A8, FXYD2, SLC20A2, ATP1A2	242	120	22680	3.123966942	1	0.464426	84.96375
GOTERM_BP_DIRECT	GO:0006814~sodium ion transport	4	1.6	0.18522	SLC17A8, FXYD2, SLC20A2, ATP1A2	217	124	18082	2.687973837	1	0.959474	96.47112

Annotation Cluster 37	Enrichment Score: 0.6675391384317532											
Category	Term	Count	%	PValue	Genes	List Total	Pop Hits	Pop Total	Fold Enrichment	Bonferroni	Benjamini	FDR
INTERPRO	IPR011993:Pleckstrin homology-like domain	8	3.2	0.19072	PLEKHG4, OBSCN, DAB2IP, PTK2B, WDFY4, PSD3, PLCD4, EPS8L2	237	409	20594	1.699648211	1	0.873702	95.36098
GOTERM_MF_DIRECT	GO:0005089~Rho guanyl-nucleotide exchange factor activity	3	1.2	0.22223	PLEKHG4, OBSCN, EPS8L2	210	74	17446	3.367953668	1	0.906485	96.98135
GOTERM_BP_DIRECT	GO:0035023~regulation of Rho protein signal transduction	3	1.2	0.23452	PLEKHG4, OBSCN, EPS8L2	217	77	18082	3.246513855	1	0.975107	98.7262

Annotation Cluster 38	Enrichment Score: 0.5933212506816464											
Category	Term	Count	%	PValue	Genes	List Total	Pop Hits	Pop Total	Fold Enrichment	Bonferroni	Benjamini	FDR
UP_SEQ_FEATURE	propeptide:Activation peptide	4	1.6	0.07843	MMP10, MMP9, CMA1, TPSB2	223	81	18012	3.988706195	1	0.81092	72.6632
GOTERM_MF_DIRECT	GO:0004252~serine-type endopeptidase activity	6	2.4	0.11159	MMP9, CMA1, HTRA3, TPSB2, PRSS23, C1S1	210	212	17446	2.351212938	1	0.768741	80.7552
UP_KEYWORDS	Serine protease	5	2	0.11379	CMA1, HTRA3, TPSB2, PRSS23, C1S1	242	173	22680	2.708641857	1	0.431791	79.14046
INTERPRO	IPR009003:Trypsin-like cysteine/serine peptidase domain	5	2	0.1279	CMA1, HTRA3, TPSB2, PRSS23, C1S1	237	168	20594	2.586146273	1	0.757516	86.2732
GOTERM_MF_DIRECT	GO:0008236~serine-type peptidase activity	5	2	0.13244	CMA1, HTRA3, TPSB2, PRSS23, C1S1	210	163	17446	2.548349401	1	0.808841	86.17432
UP_KEYWORDS	Hydrolase	22	8.8	0.2216	APOBEC2, ARSB, PTPRD, MMP9, ARSI, RAG1, CPXM2, PDE11A, ATP1A2, AMPD1, ADPRHL1, MMP10, CEMIP, PLCD4, CMA1, ENTPD3, HTRA3, INPP5D, TPSB2, C1S1, PRSS23, IHH	242	1646	22680	1.25262344	1	0.644945	96.12406
UP_KEYWORDS	Protease	9	3.6	0.22203	MMP10, MMP9, CPXM2, CMA1, HTRA3, TPSB2, PRSS23, C1S1, IHH	242	542	22680	1.556219694	1	0.639666	96.15138
UP_SEQ_FEATURE	active site:Charge relay system	5	2	0.25055	CMA1, HTRA3, TPSB2, PRSS23, C1S1	223	205	18012	1.970031718	1	0.987057	98.97421
INTERPRO	IPR001254:Peptidase S1	4	1.6	0.27791	CMA1, TPSB2, PRSS23, C1S1	237	160	20594	2.172362869	1	0.945733	99.11264
GOTERM_MF_DIRECT	GO:0008233~peptidase activity	9	3.6	0.27832	MMP10, ADAMTSL1, MMP9, CMA1, HTRA3, TPSB2, PRSS23, C1S1, IHH	210	516	17446	1.449003322	1	0.939672	98.93573
GOTERM_BP_DIRECT	GO:0006508~proteolysis	9	3.6	0.39282	MMP10, MMP9, CPXM2, CMA1, HTRA3, TPSB2, PRSS23, C1S1, IHH	217	582	18082	1.288564777	1	0.996432	99.971
UP_KEYWORDS	Zymogen	4	1.6	0.39792	MMP10, MMP9, CMA1, TPSB2	242	213	22680	1.759981376	1	0.817386	99.86162
UP_SEQ_FEATURE	domain:Peptidase S1	3	1.2	0.41121	CMA1, TPSB2, C1S1	223	114	18012	2.125560538	1	0.999415	99.97775
INTERPRO	IPR018114:Peptidase S1, trypsin family, active site	3	1.2	0.467	CMA1, TPSB2, PRSS23	237	137	20594	1.902799593	1	0.993972	99.98917
GOTERM_MF_DIRECT	GO:0016787~hydrolase activity	20	8	0.47358	APOBEC2, ARSB, PTPRD, MMP9, ARSI, RAG1, PDE11A, ATP1A2, AMPD1, ADPRHL1, MMP10, CEMIP, PLCD4, CMA1, HTRA3, INPP5D, TPSB2, PRSS23, C1S1, IHH	210	1533	17446	1.083838101	1	0.986523	99.98685
INTERPRO	IPR001314:Peptidase S1A, chymotrypsin-type	3	1.2	0.51813	CMA1, TPSB2, C1S1	237	151	20594	1.726381088	1	0.997093	99.99749
SMART	SM00020:Tryp_SPc	3	1.2	0.69167	CMA1, TPSB2, C1S1	158	158	10425	1.252804038	1	0.993969	99.9999

Annotation Cluster 39	Enrichment Score: 0.5840796108420417											
Category	Term	Count	%	PValue	Genes	List Total	Pop Hits	Pop Total	Fold Enrichment	Bonferroni	Benjamini	FDR
UP_KEYWORDS	Endoplasmic reticulum	16	6.4	0.11202	JPH2, FKBP7, ARSI, ITPR3, RTN2, JSRP1, PTRF, XYLT1, FITM1, FMO2, SLC37A2, FKBP14, CEMIP, PLCD4, ELOVL6, PIEZO1	242	997	22680	1.504016181	1	0.432548	78.59223
GOTERM_CC_DIRECT	GO:0005783~endoplasmic reticulum	19	7.6	0.32507	JPH2, FKBP7, ARSI, ITPR3, RTN2, APOD, JSRP1, PTRF, XYLT1, FITM1, FMO2, SLC37A2, CEMIP, FKBP14, PLCD4, PTN, ELOVL6, PIEZO1, AOC3	237	1323	19662	1.19144254	1	0.787249	99.39088
GOTERM_CC_DIRECT	GO:0005789~endoplasmic reticulum membrane	10	4	0.48584	FXYD3, FKBP7, XYLT1, NDRG4, FITM1, FMO2, FKBP14, ITPR3, PIEZO1, RTN2	237	710	19662	1.16847923	1	0.902081	99.98215

Annotation Cluster 40	Enrichment Score: 0.5252228432372151											
Category	Term	Count	%	PValue	Genes	List Total	Pop Hits	Pop Total	Fold Enrichment	Bonferroni	Benjamini	FDR
GOTERM_CC_DIRECT	GO:0016020~membrane	96	38.4	0.07626	JPH2, HFE2, HIP1R, SLC20A2, TRPV2, ANO1, SYT8, PRKG2, LRRC15, IGHM, DDR2, ART1, RTN2, GPC3, OCSTAMP, DES, JSRP1, FITM1, XYLT1, WNK4, PIK3AP1, LOXL4, ELOVL6, LOXL2, PIEZO1, RAMP1, ANO6, 1810041L15RIK, IHH, DAB2IP, MRLN, MFI2, PSD3, IL11RA1, SEL1L3, NFAM1, CACNG1, SSTR2, PODXL2, PTRF, CHPF, SLC37A2, CEMIP, CYBRD1, CD300LG, RYR1, SGCD, EFNA5, PMP22, CASQ1, LCP1, AOC3, FXYD1, FRAS1, FXYD2, FXYD3, CLDN19, AKAP12, CYTB, ITGB3, FXYD6, SYPL2, MYOT, FAM19A5, ITM2A, SERINC5, LECT1, ITGAX, PTK2B, PLIN4, FMO2, APOBR, PLCD4, TNFRSF19, ENO3, PTN, INPP5D, CSF1R, PTPRD, VSTM4, MPZ, COX7A1, ITGA1, DOCK8, ATP1A2, ITPR3, CACNA1S, GPR153, RERG, SLC17A8, CD55, WSCD2, NTRK2, CACNA1G, 3110079O15RIK, SCARA5	237	6998	19662	1.138090087	1	0.412407	64.27296

GOTERM_CC_DIRECT	GO:0005886~plasma membrane	68	27.2	0.11355	JPH2, HFE2, SLC20A2, TRPV2, ANO1, SYT8, PRKG2, IGHM, DDR2, GSTM2, GPC3, DES, PIK3AP1, PIEZO1, RAMP1, ANO6, IHH, DAB2IP, CRYAB, MFI2, PSD3, ACTN2, NFAM1, SIGLEC15, SSTR2, PTRF, RIF1, CEMIP, RYR1, CD300LG, SGCD, EFNA5, PMP22, EPS8L2, LCP1, AOC3, FXYD1, FRAS1, CLDN19, FHL1, AKAP12, ITGB3, FXYD6, MYOT, ITM2A, ANXA8, SERINC5, PTK2B, PLIN4, APOBR, PLCD4, ENO3, TNFRSF19, ENTPD3, INPP5D, CSF1R, VSTM4, IKZF3, MPZ, ITGA1, ATP1A2, ITPR3, CACNA1S, GPR153, CD55, NTRK2, CACNA1G, SCARA5	237	4874	19662	1.157451318	1	0.509189	79.06634
UP_SEQ_FEATURE	topological domain:Extracellular	35	14	0.12407	FXYD1, FRAS1, FXYD3, CLDN19, SLC20A2, TRPV2, ANO1, SYT8, ITGB3, LRRC15, FXYD6, DDR2, SERINC5, ITGAX, FITM1, TNFRSF19, ANO6, RAMP1, 1810041L15RIK, CSF1R, PTPRD, MPZ, ITGA1, IL11RA1, NFAM1, ITPR3, CACNA1S, GPR153, SSTR2, PODXL2, NTRK2, CD300LG, SGCD, SCARA5, AOC3	223	2256	18012	1.253100849	1	0.918315	87.79647
UP_SEQ_FEATURE	topological domain:Cytoplasmic	41	16.4	0.22752	FXYD1, FRAS1, FXYD3, CLDN19, JPH2, SLC20A2, TRPV2, ANO1, SYT8, ITGB3, LRRC15, FXYD6, DDR2, SYPL2, SERINC5, ITGAX, XYLT1, FITM1, TNFRSF19, ANO6, RAMP1, 1810041L15RIK, CSF1R, PTPRD, MPZ, ITGA1, IL11RA1, NFAM1, ATP1A2, ITPR3, CACNA1S, GPR153, SLC17A8, SSTR2, PODXL2, CHPF, NTRK2, CD300LG, SGCD, SCARA5, AOC3	223	2880	18012	1.149869208	1	0.981813	98.3411
UP_KEYWORDS	Cell membrane	45	18	0.26402	FRAS1, CLDN19, JPH2, HFE2, SLC20A2, TRPV2, ANO1, SYT8, PRKG2, ITGB3, IGHM, DDR2, MYOT, SERINC5, GPC3, DES, PTK2B, PLIN4, APOBR, TNFRSF19, PIK3AP1, INPP5D, PIEZO1, ANO6, CSF1R, IHH, VSTM4, DAB2IP, MPZ, MFI2, PSD3, NFAM1, ATP1A2, GPR153, CD55, SSTR2, PTRF, NTRK2, CEMIP, CD300LG, SGCD, EFNA5, PMP22, SCARA5, LCP1	242	3759	22680	1.121935454	1	0.700823	98.12637
UP_SEQ_FEATURE	transmembrane region	57	22.8	0.35049	JPH2, SLC20A2, TRPV2, ANO1, SYT8, LRRC15, IGHM, DDR2, RTN2, OCSTAMP, FITM1, XYLT1, ELOVL6, ANO6, RAMP1, 1810041L15RIK, IL11RA1, SEL1L3, NFAM1, CACNG1, PODXL2, SSTR2, CHPF, SLC37A2, CD300LG, CYBRD1, SGCD, PMP22, AOC3, FXYD1, FRAS1, FXYD2, FXYD3, CLDN19, CYTB, ITGB3, FXYD6, SYPL2, ITM2A, FAM19A5, SERINC5, LECT1, ITGAX, TNFRSF19, CSF1R, PTPRD, MPZ, COX7A1, ITGA1, ATP1A2, ITPR3, CACNA1S, GPR153, SLC17A8, NTRK2, 3110079O15RIK, SCARA5	223	4312	18012	1.067709677	1	0.998037	99.8943
UP_KEYWORDS	Membrane	94	37.6	0.48556	JPH2, HFE2, HIP1R, SLC20A2, TRPV2, ANO1, SYT8, PRKG2, LRRC15, IGHM, DDR2, ART1, RTN2, GPC3, OCSTAMP, DES, JSRP1, FITM1, XYLT1, SEMA3D, PIK3AP1, ELOVL6, PIEZO1, RAMP1, ANO6, 1810041L15RIK, IHH, DAB2IP, MRLN, MFI2, PSD3, IL11RA1, SEL1L3, NFAM1, CACNG1, SIGLEC15, SSTR2, PODXL2, PTRF, CHPF, SLC37A2, CEMIP, CYBRD1, CD300LG, RYR1, SGCD, EFNA5, PMP22, CASQ1, LCP1, AOC3, FXYD1, FRAS1, FXYD2, FXYD3, TM4SF19, CLDN19, AKAP12, CYTB, ITGB3, FXYD6, SYPL2, MYOT, FAM19A5, ITM2A, SERINC5, LECT1, ITGAX, PTK2B, PLIN4, FMO2, APOBR, PLCD4, TNFRSF19, ENTPD3, INPP5D, CSF1R, PTPRD, VSTM4, MPZ, COX7A1, ITGA1, ATP1A2, ITPR3, CACNA1S, GPR153, SLC17A8, CD55, ETS1, WSCD2, NTRK2, CACNA1G, 3110079O15RIK, SCARA5	242	8683	22680	1.014578691	1	0.875889	99.98202

UP_KEYWORDS	Transmembrane	72	28.8	0.68124	ASPN, JPH2, SLC20A2, TRPV2, ANO1, SYT8, LRRC15, IGHM, DDR2, RTN2, OCSTAMP, JSRP1, FITM1, XYLT1, SEMA3D, ELOVL6, PIEZO1, RAMP1, ANO6, 1810041L15RIK, MRLN, IL11RA1, SEL1L3, NFAM1, CACNG1, SIGLEC15, SSTR2, PODXL2, CHPF, SLC37A2, CYBRD1, CD300LG, RYR1, SGCD, PMP22, AOC3, FXYD1, FRAS1, FXYD2, FXYD3, CLDN19, TM4SF19, CYTB, ITGB3, FXYD6, SYPL2, FAM19A5, ITM2A, SERINC5, LECT1, ITGAX, PTK2B, FMO2, TNFRSF19, ENTPD3, CSF1R, PTPRD, VSTM4, MPZ, COX7A1, ITGA1, ATP1A2, ITPR3, CACNA1S, GPR153, SLC17A8, WSCD2, ETS1, NTRK2, CACNA1G, 3110079O15RIK, SCARA5	242	6955	22680	0.970203968	1	0.960126	99.99996
UP_KEYWORDS	Transmembrane helix	70	28	0.76729	JPH2, SLC20A2, TRPV2, ANO1, SYT8, LRRC15, IGHM, DDR2, RTN2, OCSTAMP, JSRP1, FITM1, XYLT1, SEMA3D, ELOVL6, PIEZO1, RAMP1, ANO6, 1810041L15RIK, MRLN, IL11RA1, SEL1L3, NFAM1, CACNG1, SIGLEC15, SSTR2, PODXL2, CHPF, SLC37A2, CYBRD1, CD300LG, RYR1, SGCD, PMP22, AOC3, FXYD1, FRAS1, FXYD2, FXYD3, CLDN19, TM4SF19, CYTB, ITGB3, FXYD6, SYPL2, FAM19A5, ITM2A, SERINC5, LECT1, ITGAX, PTK2B, TNFRSF19, ENTPD3, CSF1R, PTPRD, VSTM4, MPZ, COX7A1, ITGA1, ATP1A2, ITPR3, CACNA1S, GPR153, SLC17A8, WSCD2, ETS1, NTRK2, CACNA1G, 3110079O15RIK, SCARA5	242	6938	22680	0.945565088	1	0.982003	100
GOTERM_CC_DIRECT	GO:0016021~integral component of membrane	70	28	0.97448	JPH2, SLC20A2, TRPV2, ANO1, SYT8, LRRC15, IGHM, DDR2, RTN2, OCSTAMP, JSRP1, FITM1, XYLT1, SEMA3D, ELOVL6, PIEZO1, RAMP1, ANO6, 1810041L15RIK, MRLN, IL11RA1, SEL1L3, NFAM1, CACNG1, SIGLEC15, SSTR2, PODXL2, CHPF, SLC37A2, CYBRD1, CD300LG, RYR1, SGCD, PMP22, AOC3, FXYD1, FRAS1, FXYD2, FXYD3, CLDN19, TM4SF19, CYTB, ITGB3, FXYD6, SYPL2, FAM19A5, ITM2A, SERINC5, LECT1, ITGAX, PTK2B, FMO2, TNFRSF19, ENTPD3, CSF1R, PTPRD, VSTM4, MPZ, COX7A1, ITGA1, ATP1A2, ITPR3, CACNA1S, GPR153, SLC17A8, WSCD2, ETS1, NTRK2, 3110079O15RIK, SCARA5	237	6878	19662	0.84433582	1	0.999988	100

Annotation Cluster 41	Enrichment Score: 0.5182688408608292											
Category	Term	Count	%	PValue	Genes	List Total	Pop Hits	Pop Total	Fold Enrichment	Bonferroni	Benjamini	FDR
INTERPRO	IPR008266:Tyrosine-protein kinase, active site	5	2	0.02718	OBSCN, PTK2B, NTRK2, DDR2, CSF1R	237	99	20594	4.388611857	1	0.340125	32.95857
INTERPRO	IPR020635:Tyrosine-protein kinase, catalytic domain	4	1.6	0.06617	PTK2B, NTRK2, DDR2, CSF1R	237	81	20594	4.291087149	1	0.568552	62.96787
GOTERM_CC_DIRECT	GO:0014069~postsynaptic density	7	2.8	0.06884	PTK2B, CRYAB, HIP1R, NTRK2, PSD3, MYLK2, DCLK1	237	239	19662	2.429850114	1	0.405638	60.36409
GOTERM_BP_DIRECT	GO:0007169~transmembrane receptor protein tyrosine kinase signaling pathway	4	1.6	0.11779	NTRK2, DDR2, CSF1R, LCP2	217	100	18082	3.333087558	1	0.8859	87.07644
UP_KEYWORDS	Tyrosine-protein kinase	4	1.6	0.1193	PTK2B, NTRK2, DDR2, CSF1R	242	113	22680	3.317487018	1	0.436084	80.76293
SMART	SM00219:TyrKc	4	1.6	0.12285	PTK2B, NTRK2, DDR2, CSF1R	158	81	10425	3.258321613	1	0.580054	78.66196
GOTERM_MF_DIRECT	GO:0004714~transmembrane receptor protein tyrosine kinase activity	3	1.2	0.13666	NTRK2, DDR2, CSF1R	210	54	17446	4.615343915	1	0.810342	87.08195
UP_SEQ_FEATURE	active site:Proton acceptor	13	5.2	0.16852	OBSCN, MYLK2, PRKG2, DHRS7C, DDR2, PTK2B, WNK4, ALDH1A3, NTRK2, ENO3, DCLK1, CSF1R, AOC3	223	710	18012	1.478911135	1	0.950157	94.66219
GOTERM_MF_DIRECT	GO:0004713~protein tyrosine kinase activity	4	1.6	0.17729	PTK2B, NTRK2, DDR2, CSF1R	210	121	17446	2.746320346	1	0.855145	93.39958
GOTERM_BP_DIRECT	GO:0018108~peptidyl-tyrosine phosphorylation	3	1.2	0.18213	PTK2B, DDR2, CSF1R	217	65	18082	3.845870259	1	0.958623	96.24647
GOTERM_BP_DIRECT	GO:0008284~positive regulation of cell proliferation	10	4	0.20158	PTK2B, TBX2, ETS1, NTRK2, PTN, IL11RA1, DDR2, CLEC11A, IHH, CSF1R	217	542	18082	1.53740201	1	0.962586	97.46581
INTERPRO	IPR001245:Serine-threonine/tyrosine-protein kinase catalytic domain	4	1.6	0.21383	PTK2B, NTRK2, DDR2, CSF1R	237	139	20594	2.500561576	1	0.900694	96.95283

INTERPRO	IPR000719:Protein kinase, catalytic domain	9	3.6	0.23884	OBSCN, PTK2B, WNK4, NTRK2, MYLK2, PRKG2, DDR2, DCLK1, CSF1R	237	515	20594	1.518544918	1	0.920185	98.09404
INTERPRO	IPR017441:Protein kinase, ATP binding site	7	2.8	0.30396	OBSCN, PTK2B, NTRK2, MYLK2, PRKG2, DCLK1, CSF1R	237	394	20594	1.543811176	1	0.958774	99.47942
GOTERM_MF_DIRECT	GO:0004672~protein kinase activity	9	3.6	0.30588	OBSCN, PTK2B, WNK4, NTRK2, MYLK2, PRKG2, DDR2, DCLK1, CSF1R	210	531	17446	1.408071025	1	0.947698	99.38123
INTERPRO	IPR011009:Protein kinase-like domain	9	3.6	0.30677	OBSCN, PTK2B, WNK4, NTRK2, MYLK2, PRKG2, DDR2, DCLK1, CSF1R	237	556	20594	1.406565887	1	0.958055	99.50907
UP_KEYWORDS	Kinase	10	4	0.33829	OBSCN, PTK2B, WNK4, NTRK2, AKAP12, MYLK2, PRKG2, DDR2, DCLK1, CSF1R	242	707	22680	1.325587104	1	0.773299	99.52872
GOTERM_BP_DIRECT	GO:0046777~protein autophosphorylation	4	1.6	0.373	NTRK2, MYLK2, DDR2, CSF1R	217	183	18082	1.821359321	1	0.995965	99.95101
GOTERM_BP_DIRECT	GO:0006468~protein phosphorylation	9	3.6	0.38282	OBSCN, PTK2B, WNK4, NTRK2, MYLK2, PRKG2, DDR2, DCLK1, CSF1R	217	576	18082	1.301987327	1	0.996104	99.96213
GOTERM_MF_DIRECT	GO:0016301~kinase activity	10	4	0.41805	OBSCN, PTK2B, WNK4, NTRK2, AKAP12, MYLK2, PRKG2, DDR2, DCLK1, CSF1R	210	674	17446	1.232584428	1	0.979716	99.94687
UP_SEQ_FEATURE	domain:Protein kinase	8	3.2	0.42806	PTK2B, WNK4, NTRK2, MYLK2, PRKG2, DDR2, DCLK1, CSF1R	223	502	18012	1.287192039	1	0.999568	99.98597
UP_SEQ_FEATURE	binding site:ATP	9	3.6	0.42932	OBSCN, PTK2B, WNK4, NTRK2, MYLK2, PRKG2, DDR2, DCLK1, CSF1R	223	583	18012	1.246898292	1	0.999536	99.98645
GOTERM_BP_DIRECT	GO:0016310~phosphorylation	9	3.6	0.44703	OBSCN, PTK2B, WNK4, NTRK2, MYLK2, PRKG2, DDR2, DCLK1, CSF1R	217	612	18082	1.225399837	1	0.998361	99.9937
UP_KEYWORDS	ATP-binding	16	6.4	0.48247	PRKAG3, OBSCN, ACTA2, MYH2, MYLK2, ATP1A2, PRKG2, DDR2, MYO18B, PTK2B, WNK4, NTRK2, MYH11, KIF21B, DCLK1, CSF1R	242	1363	22680	1.100149767	1	0.87684	99.98057
INTERPRO	IPR008271:Serine/threonine-protein kinase, active site	5	2	0.53245	OBSCN, WNK4, MYLK2, PRKG2, DCLK1	237	333	20594	1.304722444	1	0.9975	99.99838
UP_KEYWORDS	Serine/threonine-protein kinase	5	2	0.62684	OBSCN, WNK4, MYLK2, PRKG2, DCLK1	242	405	22680	1.157024793	1	0.943645	99.99972
UP_SEQ_FEATURE	nucleotide phosphate-binding region:ATP	12	4.8	0.64566	OBSCN, PTK2B, WNK4, NTRK2, MYH2, MYH11, MYLK2, PRKG2, DDR2, KIF21B, DCLK1, CSF1R	223	963	18012	1.006495956	1	0.999998	99.99999
GOTERM_MF_DIRECT	GO:0004674~protein serine/threonine kinase activity	5	2	0.75761	OBSCN, WNK4, MYLK2, PRKG2, DCLK1	210	428	17446	0.970516244	1	0.999797	100
UP_KEYWORDS	Nucleotide-binding	17	6.8	0.77239	PRKAG3, OBSCN, ACTA2, MYH2, MYLK2, ATP1A2, PRKG2, DDR2, RERG, MYO18B, PTK2B, WNK4, NTRK2, MYH11, KIF21B, DCLK1, CSF1R	242	1754	22680	0.908337024	1	0.982293	100
GOTERM_MF_DIRECT	GO:0005524~ATP binding	16	6.4	0.80879	PRKAG3, OBSCN, ACTA2, MYH2, MYLK2, ATP1A2, PRKG2, DDR2, MYO18B, PTK2B, WNK4, NTRK2, MYH11, KIF21B, DCLK1, CSF1R	210	1507	17446	0.882029892	1	0.999914	100
SMART	SM00220:S_TKc	5	2	0.82951	OBSCN, WNK4, MYLK2, PRKG2, DCLK1	158	380	10425	0.868171219	1	0.999277	100
UP_KEYWORDS	Transferase	15	6	0.84568	OBSCN, AKAP12, MYLK2, PRKG2, DDR2, ART1, GSTM2, XYLT1, PTK2B, CHPF, WNK4, NTRK2, ELOVL6, DCLK1, CSF1R	242	1654	22680	0.849930547	1	0.992775	100
GOTERM_MF_DIRECT	GO:0016740~transferase activity	14	5.6	0.90545	OBSCN, MYLK2, PRKG2, DDR2, ART1, GSTM2, PTK2B, CHPF, XYLT1, WNK4, NTRK2, ELOVL6, DCLK1, CSF1R	210	1472	17446	0.790126812	1	0.999997	100
GOTERM_MF_DIRECT	GO:0000166~nucleotide binding	15	6	0.98859	PRKAG3, OBSCN, ACTA2, MYLK2, ATP1A2, PRKG2, DDR2, RERG, PTK2B, WNK4, NTRK2, MYH11, KIF21B, DCLK1, CSF1R	210	1936	17446	0.643668831	1	1	100

Annotation Cluster 42 Enrichment Score: 0.44236492428571583

Category	Term	Count	%	PValue	Genes	List Total	Pop Hits	Pop Total	Fold Enrichment	Bonferroni	Benjamini	FDR
INTERPRO	IPR011993:Pleckstrin homology-like domain	8	3.2	0.19072	PLEKHG4, OBSCN, DAB2IP, PTK2B, WDFY4, PSD3, PLCD4, EPS8L2	237	409	20594	1.699648211	1	0.873702	95.36098
INTERPRO	IPR001849:Pleckstrin homology domain	5	2	0.35356	PLEKHG4, OBSCN, DAB2IP, PSD3, PLCD4	237	262	20594	1.658292267	1	0.974173	99.82189
UP_SEQ_FEATURE	domain:PH	4	1.6	0.47411	OBSCN, DAB2IP, PSD3, PLCD4	223	208	18012	1.553294239	1	0.99981	99.9963
SMART	SM00233:PH	5	2	0.53186	PLEKHG4, OBSCN, DAB2IP, PSD3, PLCD4	158	253	10425	1.303972582	1	0.970301	99.98695

Annotation Cluster 43 Enrichment Score: 0.39449046380947983

Category	Term	Count	%	PValue	Genes	List Total	Pop Hits	Pop Total	Fold Enrichment	Bonferroni	Benjamini	FDR
UP_KEYWORDS	Protease inhibitor	3	1.2	0.36893	GPC3, WFDC1, SERPING1	242	121	22680	2.323611775	1	0.804246	99.74524
GOTERM_BP_DIRECT	GO:0010466~negative regulation of peptidase activity	3	1.2	0.40861	GPC3, WFDC1, SERPING1	217	117	18082	2.136594588	1	0.997211	99.98114

GOTERM_MF_DIRECT	GO:0030414~peptidase inhibitor activity	3	1.2	0.43479	GPC3, WFDC1, SERPING1	210	123	17446	2.026248548	1	0.979668	99.96461

Annotation Cluster 44	Enrichment Score: 0.251300539892727											
Category	Term	Count	%	PValue	Genes	List Total	Pop Hits	Pop Total	Fold Enrichment	Bonferroni	Benjamini	FDR
INTERPRO	IPR001452:Src homology-3 domain	4	1.6	0.44444	MIA, OBSCN, NEB, EPS8L2	237	212	20594	1.639519147	1	0.992165	99.98024
UP_SEQ_FEATURE	domain:SH3	3	1.2	0.60928	MIA, OBSCN, EPS8L2	223	166	18012	1.459722297	1	0.999994	99.99997
UP_KEYWORDS	SH3 domain	3	1.2	0.65083	MIA, OBSCN, EPS8L2	242	208	22680	1.351716465	1	0.950181	99.99988

Annotation Cluster 45	Enrichment Score: 0.22253697886031373											
Category	Term	Count	%	PValue	Genes	List Total	Pop Hits	Pop Total	Fold Enrichment	Bonferroni	Benjamini	FDR
UP_KEYWORDS	Lipid biosynthesis	3	1.2	0.51375	PRKAG3, SERINC5, ELOVL6	242	160	22680	1.757231405	1	0.89303	99.99135
GOTERM_BP_DIRECT	GO:0006629~lipid metabolic process	6	2.4	0.64421	PRKAG3, SERINC5, APOD, APOBR, PLCD4, ELOVL6	217	459	18082	1.0892443	1	0.999962	100
UP_KEYWORDS	Lipid metabolism	5	2	0.64955	PRKAG3, SERINC5, APOBR, PLCD4, ELOVL6	242	417	22680	1.123729116	1	0.951378	99.99988

Annotation Cluster 46	Enrichment Score: 0.024799280962238057											
Category	Term	Count	%	PValue	Genes	List Total	Pop Hits	Pop Total	Fold Enrichment	Bonferroni	Benjamini	FDR
GOTERM_MF_DIRECT	GO:0043565~sequence-specific DNA binding	7	2.8	0.77425	IKZF3, BACH2, TBX2, ETS1, RAG1, SPIB, PRRX2	210	633	17446	0.918694049	1	0.999849	100
GOTERM_CC_DIRECT	GO:0005667~transcription factor complex	3	1.2	0.83309	TBX2, ETS1, SPIB	237	267	19662	0.932157588	1	0.996628	100
GOTERM_BP_DIRECT	GO:0000122~negative regulation of transcription from RNA polymerase II promoter	7	2.8	0.87214	DAB2IP, BACH2, HFE2, TBX2, NFIX, PKIA, TWIST1	217	729	18082	0.800123899	1	1	100
UP_KEYWORDS	Repressor	4	1.6	0.92528	BACH2, NFIX, LOXL2, TWIST1	242	534	22680	0.702015043	1	0.998771	100
UP_KEYWORDS	Activator	4	1.6	0.96347	BACH2, NFIX, SPIB, TWIST1	242	624	22680	0.600762873	1	0.999789	100
UP_KEYWORDS	DNA-binding	11	4.4	0.97868	IKZF3, BACH2, TCEA3, TBX2, ETS1, HR, RAG1, NFIX, SPIB, PRRX2, TWIST1	242	1604	22680	0.642711403	1	0.999935	100
GOTERM_MF_DIRECT	GO:0003700~transcription factor activity, sequence-specific DNA binding	6	2.4	0.98263	BACH2, TBX2, ETS1, NFIX, SPIB, TWIST1	210	883	17446	0.564504126	1	1	100
UP_KEYWORDS	Transcription regulation	11	4.4	0.9937	IKZF3, BACH2, TCEA3, PTRF, TBX2, ETS1, HR, NFIX, SPIB, LOXL2, TWIST1	242	1799	22680	0.573045631	1	0.999995	100
UP_KEYWORDS	Transcription	11	4.4	0.99577	IKZF3, BACH2, TCEA3, PTRF, TBX2, ETS1, HR, NFIX, SPIB, LOXL2, TWIST1	242	1859	22680	0.554550345	1	0.999998	100
GOTERM_BP_DIRECT	GO:0006351~transcription, DNA-templated	12	4.8	0.99835	IKZF3, BACH2, TCEA3, PTRF, TBX2, ETS1, ABLIM3, HR, NFIX, SPIB, LOXL2, TWIST1	217	1885	18082	0.530464863	1	1	100
GOTERM_MF_DIRECT	GO:0003677~DNA binding	11	4.4	0.99918	IKZF3, BACH2, TCEA3, TBX2, ETS1, HR, RAG1, NFIX, SPIB, PRRX2, TWIST1	210	1847	17446	0.494768866	1	1	100
GOTERM_BP_DIRECT	GO:0006355~regulation of transcription, DNA-templated	12	4.8	0.99994	IKZF3, BACH2, TCEA3, PTRF, TBX2, ETS1, HR, NFIX, SPIB, PRRX2, LOXL2, TWIST1	217	2279	18082	0.438756589	1	1	100
UP_KEYWORDS	Nucleus	23	9.2	1	IKZF3, BACH2, CRYAB, TBX2, FHL1, NELL1, ENC1, HR, RAG1, NFIX, PRRX2, SMTNL1, MUSTN1, TCEA3, RIF1, PTRF, PTK2B, ETS1, CEMIP, PLCD4, SPIB, LOXL2, TWIST1	242	4534	22680	0.475416231	1	1	100
