# Supplementary material for: Predicted mouse interactome and network-based interpretation of differentially expressed genes
Source: PLoS One. 2022 Apr 7;17(4):e0264174. doi: 10.1371/journal.pone.0264174 (PMC8989236; doi:10.1371/journal.pone.0264174)
Supplement: S8 Table — (PDF) [file pone.0264174.s009.pdf]

Table S8. Functional annotations reported by MID/GSLA for the top 250 transcriptionally changed genes between the wild type and Piezo1-knockout mice.

Job Number: GSLA07275824497532
Cutoff used:
Q1: density >= 0.015
Q2: p <= 0.00001
Meaning:
Q1: inter-geneset interaction density is greater than expected.
Q2: the observed interaction density can only be observed in the biologically correct interactome topology.
Categories selected: GO biological process
Species: Mus musculus
Interaction dataset selected: MID

#Subjob Number: GSLA07275824497532_0
#Description: #bone-TOP50

#Quaried GeneSet: Acan, Ihh, Hapln1, Arsi, 3110079O15Rik, Col9a1, Apod, Col10a1, Igf2, Aldh1a3, Col9a3, Wnk4, Col9a2, Col2a1, Matn3, Sstr2, Dcn, Piezo1, Loxl4, Epyc, H19, Pcolce2, Itgb3, Col8a2, Ogn, Fras1, Eps8l2, Ptn, Ostn, Plin4, Mia, Cpxm2, Tagln, Cryab, Igfbp6, Htra3, Lcp1, Ano1, Mfi2, Loxl2, Fxyd3, Pik3ap1, Scin, Mmp10, Il11ra1, Sema3c, Aspn, Inpp5d, Tcea3, Itgbl1

<table>
<tr><th>Term type</th><th>Term</th><th>Description</th><th>Term size</th><th>P value</th><th>Density</th><th>Interaction number</th><th>Overlap gene number</th><th>Overlap gene(s)</th><th>Interactions</th></tr>
<tr><td>GO biological process</td><td>GO:0005583</td><td>fibrillar collagen trimer</td><td>10</td><td>0</td><td>0.03469</td><td>17</td><td>1</td><td>Col2a1</td><td>Col2a1-MGI:88468|Col2a1-MGI:88467|Col2a1-MGI:88457|Col2a1-MGI:88458|Col2a1-MGI:1858212|Col2a1-MGI:88446|Col2a1-MGI:88453|Col2a1-MGI:2672118|Dcn-MGI:88468|Dcn-MGI:88467|Dcn-MGI:88457|Dcn-MGI:88458|Dcn-MGI:1858212|Dcn-MGI:109347|Dcn-MGI:88446|Dcn-MGI:88453|Dcn-MGI:2672118</td></tr>
<tr><td>GO biological process</td><td>GO:1900086</td><td>positive regulation of peptidyl-tyrosine autophosphorylation</td><td>6</td><td>0</td><td>0.03401</td><td>10</td><td>0</td><td></td><td>Cryab-MGI:103178|Apod-MGI:1344337|Wnk4-MGI:105100|Inpp5d-MGI:105100|Inpp5d-MGI:99515|Itgb3-MGI:1344337|Itgb3-MGI:105100|Itgb3-MGI:99515|Itgb3-MGI:1352757|Itgb3-MGI:103178</td></tr>
<tr><td>GO biological process</td><td>GO:0015379</td><td>potassium:chloride symporter activity</td><td>4</td><td>0</td><td>0.03061</td><td>6</td><td>0</td><td></td><td>Ano1-MGI:103150|Ano1-MGI:2135960|Ano1-MGI:101924|Ano1-MGI:1862037|Wnk4-MGI:1862037|Itgb3-MGI:101924</td></tr>
<tr><td>GO biological process</td><td>GO:1905310</td><td>regulation of cardiac neural crest cell migration involved in outflow tract morphogenesis</td><td>4</td><td>0</td><td>0.03061</td><td>6</td><td>1</td><td>Sema3c</td><td>Col2a1-MGI:88180|Col2a1-MGI:103302|Sema3c-MGI:95392|Itgb3-MGI:88180|Itgb3-MGI:95392|Itgb3-MGI:103302</td></tr>
<tr><td>GO biological process</td><td>GO:1902897</td><td>regulation of postsynaptic density protein 95 clustering</td><td>4</td><td>0</td><td>0.03061</td><td>6</td><td>0</td><td></td><td>Scin-MGI:1931838|Lcp1-MGI:1931838|Ptn-MGI:1931838|Ptn-MGI:88355|Itgb3-MGI:1931838|Itgb3-MGI:88355</td></tr>
<tr><td>GO biological process</td><td>GO:0007435</td><td>salivary gland morphogenesis</td><td>8</td><td>0</td><td>0.02806</td><td>11</td><td>0</td><td></td><td>Sstr2-MGI:95294|Col2a1-MGI:103302|Aldh1a3-MGI:97490|Wnk4-MGI:105100|Wnk4-MGI:95522|Inpp5d-MGI:105100|Inpp5d-MGI:95522|Itgb3-MGI:105100|Itgb3-MGI:95522|Itgb3-MGI:95294|Itgb3-MGI:103302</td></tr>
<tr><td>GO biological process</td><td>GO:0071801</td><td>regulation of podosome assembly</td><td>18</td><td>0</td><td>0.02721</td><td>24</td><td>1</td><td>Lcp1</td><td>Cryab-MGI:98397|Cryab-MGI:103264|Scin-MGI:95851|Scin-MGI:1923959|Dcn-MGI:98397|Igf2-MGI:104798|Lcp1-MGI:95851|Lcp1-MGI:1919583|Lcp1-MGI:97167|Lcp1-MGI:98397|Lcp1-MGI:1923959|Lcp1-MGI:1346861|Lcp1-MGI:1096342|Lcp1-MGI:1352745|Lcp1-MGI:96052|Lcp1-MGI:103264|Ptn-MGI:95851|Ptn-MGI:98397|Inpp5d-MGI:98397|Inpp5d-MGI:96052|Itgb3-MGI:97167|Itgb3-MGI:98397|Itgb3-MGI:1923959|Itgb3-MGI:1096342</td></tr>
<tr><td>GO biological process</td><td>GO:0036120</td><td>cellular response to platelet-derived growth factor stimulus</td><td>17</td><td>0</td><td>0.02641</td><td>22</td><td>1</td><td>Itgb3</td><td>Cryab-MGI:98397|Scin-MGI:1923959|Scin-MGI:1345963|Dcn-MGI:98397|Lcp1-MGI:95602|Lcp1-MGI:98397|Lcp1-MGI:1923959|Lcp1-MGI:97860|Lcp1-MGI:1345963|Ptn-MGI:95602|Ptn-MGI:98397|Inpp5d-MGI:95602|Inpp5d-MGI:98397|Inpp5d-Itgb3|Itgb3-MGI:1096393|Itgb3-MGI:107821|Itgb3-MGI:106590|Itgb3-MGI:95602|Itgb3-MGI:98397|Itgb3-MGI:1923959|Itgb3-MGI:97599|Itgb3-MGI:1352757</td></tr>
</table>

<table>
<tr><th></th><th></th><th></th><th></th><th></th><th></th><th></th><th></th><th></th><th></th></tr>
<tr><td>GO biological process</td><td>GO:0070700</td><td>BMP receptor binding</td><td>11</td><td>0</td><td>0.02597</td><td>14</td><td>0</td><td></td><td>Cryab-MGI:98397|Col2a1-MGI:88180|Col2a1-MGI:103302|Dcn-MGI:98397|Igf2-MGI:88177|Igf2-MGI:88182|Lcp1-MGI:98397|Ptn-MGI:98397|Inpp5d-MGI:98397|Itgb3-MGI:88177|Itgb3-MGI:88180|Itgb3-MGI:105057|Itgb3-MGI:103302|Itgb3-MGI:98397</td></tr>
<tr><td>GO biological process</td><td>GO:0042567</td><td>insulin-like growth factor ternary complex</td><td>4</td><td>0</td><td>0.02551</td><td>5</td><td>0</td><td></td><td>Igf2-MGI:96432|Igfbp6-MGI:96440|Igfbp6-MGI:96432|Igfbp6-MGI:96438|Itgb3-MGI:96438</td></tr>
<tr><td>GO biological process</td><td>GO:0071437</td><td>invadopodium</td><td>9</td><td>0</td><td>0.02494</td><td>11</td><td>0</td><td></td><td>Scin-MGI:1923959|Scin-MGI:1913963|Lcp1-MGI:1923959|Lcp1-MGI:97167|Lcp1-MGI:1352745|Lcp1-MGI:98931|Lcp1-MGI:1913963|Itgb3-MGI:103098|Itgb3-MGI:1923959|Itgb3-MGI:97167|Itgb3-MGI:98931</td></tr>
<tr><td>GO biological process</td><td>GO:0031994</td><td>insulin-like growth factor I binding</td><td>11</td><td>0</td><td>0.02411</td><td>13</td><td>2</td><td>Igfbp6|Itgb3</td><td>Igf2-MGI:96437|Wnk4-MGI:96575|Igfbp6-MGI:96440|Igfbp6-MGI:96438|Inpp5d-MGI:96575|Inpp5d-Itgb3|Itgb3-MGI:96605|Itgb3-MGI:96433|Itgb3-MGI:96613|Itgb3-MGI:96608|Itgb3-MGI:96575|Itgb3-MGI:96438|Itgb3-MGI:96437</td></tr>
<tr><td>GO biological process</td><td>GO:0045159</td><td>myosin II binding</td><td>6</td><td>0</td><td>0.0238</td><td>7</td><td>0</td><td></td><td>Scin-MGI:95851|Eps8l2-MGI:1349482|Lcp1-MGI:95851|Lcp1-MGI:2685570|Ptn-MGI:95851|Wnk4-MGI:1349482|Itgb3-MGI:1349482</td></tr>
<tr><td>GO biological process</td><td>GO:0031995</td><td>insulin-like growth factor II binding</td><td>6</td><td>0</td><td>0.0238</td><td>7</td><td>1</td><td>Igfbp6</td><td>Igf2-MGI:96437|Wnk4-MGI:96575|Igfbp6-MGI:96438|Inpp5d-MGI:96575|Itgb3-MGI:96575|Itgb3-MGI:96438|Itgb3-MGI:96437</td></tr>
<tr><td>GO biological process</td><td>GO:0007229</td><td>integrin-mediated signaling pathway</td><td>53</td><td>0</td><td>0.0231</td><td>60</td><td>1</td><td>Ptn</td><td>Cryab-MGI:98397|Cryab-Ptn|Tagln-MGI:95537|Col2a1-MGI:88453|Col2a1-MGI:95537|Ano1-MGI:96600|Dcn-MGI:106206|Dcn-MGI:88453|Dcn-MGI:98397|Sema3c-MGI:106206|Eps8l2-MGI:1888518|Eps8l2-MGI:98923|Lcp1-MGI:108295|Lcp1-MGI:103072|Lcp1-MGI:1888518|Lcp1-MGI:98397|Lcp1-MGI:95481|Lcp1-MGI:98923|Lcp1-MGI:2385001|Lcp1-MGI:1096342|Lcp1-MGI:87859|Ptn-MGI:98397|Wnk4-MGI:95481|Inpp5d-MGI:1860485|Inpp5d-MGI:99515|Inpp5d-MGI:99511|Inpp5d-MGI:1888518|Inpp5d-MGI:2147790|Inpp5d-MGI:108091|Inpp5d-MGI:98397|Inpp5d-MGI:1333854|Inpp5d-MGI:1277211|Inpp5d-MGI:98923|Inpp5d-MGI:95496|Inpp5d-MGI:98747|Inpp5d-MGI:96616|Inpp5d-MGI:95527|Inpp5d-MGI:2385001|Inpp5d-MGI:87859|Itgb3-MGI:96604|Itgb3-MGI:1860485|Itgb3-MGI:1099832|Itgb3-MGI:108295|Itgb3-MGI:99515|Itgb3-MGI:96600|Itgb3-MGI:2147790|Itgb3-MGI:98397|Itgb3-MGI:102700|Itgb3-MGI:104908|Itgb3-MGI:95496|Itgb3-MGI:96614|Itgb3-MGI:96616|Itgb3-MGI:1195267|Itgb3-MGI:1350360|Itgb3-MGI:95527|Itgb3-MGI:1096342|Itgb3-MGI:96610|Itgb3-MGI:96611|Itgb3-MGI:87859|Itgb3-MGI:2442114</td></tr>
<tr><td>GO biological process</td><td>GO:0031258</td><td>lamellipodium membrane</td><td>8</td><td>0</td><td>0.02295</td><td>9</td><td>1</td><td>Itgb3</td><td>Scin-MGI:2449316|Lcp1-MGI:2449316|Inpp5d-Itgb3|Itgb3-MGI:103098|Itgb3-MGI:96608|Itgb3-MGI:1346052|Itgb3-MGI:2449316|Itgb3-MGI:1916788|Itgb3-MGI:88338</td></tr>
<tr><td>GO biological process</td><td>GO:0051016</td><td>barbed-end actin filament capping</td><td>10</td><td>0</td><td>0.02244</td><td>11</td><td>0</td><td></td><td>Cryab-MGI:87918|Cryab-MGI:104652|Scin-MGI:97887|Scin-MGI:104684|Scin-MGI:700006|Scin-MGI:1346078|Scin-MGI:87918|Scin-MGI:104652|Scin-MGI:87919|Eps8l2-MGI:104684|Lcp1-MGI:87918</td></tr>
<tr><td>GO biological process</td><td>GO:0072111</td><td>cell proliferation involved in kidney development</td><td>10</td><td>0</td><td>0.02244</td><td>11</td><td>0</td><td></td><td>Cryab-MGI:95295|Col2a1-MGI:88180|Col2a1-MGI:103302|Igf2-MGI:88177|Itgb3-MGI:88177|Itgb3-MGI:97528|Itgb3-MGI:95295|Itgb3-MGI:88180|Itgb3-MGI:105373|Itgb3-MGI:97531|Itgb3-MGI:103302</td></tr>
<tr><td>GO biological process</td><td>GO:0051693</td><td>actin filament capping</td><td>15</td><td>0</td><td>0.02176</td><td>16</td><td>1</td><td>Scin</td><td>Cryab-MGI:87918|Cryab-MGI:104652|Scin-MGI:95851|Scin-MGI:97887|Scin-MGI:1346078|Scin-MGI:87918|Scin-MGI:87919|Scin-MGI:98809|Scin-MGI:107717|Scin-MGI:104684|Scin-MGI:700006|Scin-MGI:104652|Eps8l2-MGI:104684|Lcp1-MGI:95851|Lcp1-MGI:87918|Ptn-MGI:95851</td></tr>
</table>

<table>
<tr><td>GO biological process</td><td>GO:0031527</td><td>filopodium membrane</td><td>16</td><td>0</td><td>0.02168</td><td>17</td><td>1</td><td>Itgb3</td><td>Cryab-MGI:94909|Scin-MGI:2449316|Lcp1-MGI:97167|Lcp1-MGI:2449316|Wnk4-MGI:94909|Inpp5d-Itgb3|Itgb3-MGI:104631|Itgb3-MGI:103098|Itgb3-MGI:96602|Itgb3-MGI:94909|Itgb3-MGI:96608|Itgb3-MGI:97167|Itgb3-MGI:1261814|Itgb3-MGI:2685104|Itgb3-MGI:99435|Itgb3-MGI:2449316|Itgb3-MGI:1916788</td></tr>
<tr><td>GO biological process</td><td>GO:0008305</td><td>integrin complex</td><td>25</td><td>0</td><td>0.02122</td><td>26</td><td>1</td><td>Itgb3</td><td>Ano1-MGI:96600|Lcp1-MGI:96892|Inpp5d-MGI:96892|Inpp5d-MGI:96616|Inpp5d-Itgb3|Itgb3-MGI:96605|Itgb3-MGI:96604|Itgb3-MGI:96603|Itgb3-MGI:96599|Itgb3-MGI:96602|Itgb3-MGI:96608|Itgb3-MGI:96606|Itgb3-MGI:1338035|Itgb3-MGI:96600|Itgb3-MGI:96892|Itgb3-MGI:96614|Itgb3-MGI:96613|Itgb3-MGI:96616|Itgb3-MGI:96615|Itgb3-MGI:97631|Itgb3-MGI:104756|Itgb3-MGI:1350360|Itgb3-MGI:96610|Itgb3-MGI:96611|Itgb3-MGI:2442114|Itgb3-MGI:102700</td></tr>
<tr><td>GO biological process</td><td>GO:0006971</td><td>hypotonic response</td><td>9</td><td>0</td><td>0.0204</td><td>9</td><td>0</td><td></td><td>Ano1-MGI:2135960|Ano1-MGI:96600|Ano1-MGI:1862037|Lcp1-MGI:894806|Wnk4-MGI:108173|Wnk4-MGI:1858416|Wnk4-MGI:1862037|Itgb3-MGI:1858416|Itgb3-MGI:96600</td></tr>
<tr><td>GO biological process</td><td>GO:0008290</td><td>F-actin capping protein complex</td><td>7</td><td>0</td><td>0.0204</td><td>7</td><td>0</td><td></td><td>Cryab-MGI:87918|Cryab-MGI:104652|Scin-MGI:106222|Scin-MGI:87918|Scin-MGI:104652|Scin-MGI:87919|Lcp1-MGI:87918</td></tr>
<tr><td>GO biological process</td><td>GO:0004720</td><td>protein-lysine 6-oxidase activity</td><td>4</td><td>0</td><td>0.0204</td><td>4</td><td>2</td><td>Loxl4|Loxl2</td><td>Loxl4-MGI:96817|Loxl4-MGI:1337004|Loxl2-MGI:96817|Loxl2-MGI:1337004</td></tr>
<tr><td>GO biological process</td><td>GO:0002102</td><td>podosome</td><td>25</td><td>0</td><td>0.0204</td><td>25</td><td>1</td><td>Lcp1</td><td>Cryab-MGI:98397|Cryab-MGI:103264|Scin-MGI:95851|Scin-MGI:700006|Dcn-MGI:98397|Lcp1-MGI:1919583|Lcp1-MGI:98927|Lcp1-MGI:1346861|Lcp1-MGI:95851|Lcp1-MGI:87904|Lcp1-MGI:98397|Lcp1-MGI:1298393|Lcp1-MGI:1352745|Lcp1-MGI:103264|Ptn-MGI:95851|Ptn-MGI:98397|Inpp5d-MGI:98926|Inpp5d-MGI:107825|Inpp5d-MGI:2147790|Inpp5d-MGI:98397|Inpp5d-MGI:2147677|Itgb3-MGI:98926|Itgb3-MGI:107825|Itgb3-MGI:2147790|Itgb3-MGI:98397</td></tr>
<tr><td>GO biological process</td><td>GO:0048407</td><td>platelet-derived growth factor binding</td><td>12</td><td>0</td><td>0.0204</td><td>12</td><td>1</td><td>Col2a1</td><td>Col2a1-MGI:88468|Col2a1-MGI:88467|Col2a1-MGI:88457|Col2a1-MGI:88453|Dcn-MGI:88468|Dcn-MGI:88467|Dcn-MGI:88457|Dcn-MGI:88453|Sema3c-MGI:97527|Ptn-MGI:97527|Itgb3-MGI:97528|Itgb3-MGI:97531</td></tr>
<tr><td>GO biological process</td><td>GO:0033622</td><td>integrin activation</td><td>9</td><td>0</td><td>0.0204</td><td>9</td><td>0</td><td></td><td>Sema3c-MGI:95566|Lcp1-MGI:2385001|Inpp5d-MGI:2385001|Inpp5d-MGI:95566|Inpp5d-MGI:2147790|Itgb3-MGI:103556|Itgb3-MGI:1099832|Itgb3-MGI:95566|Itgb3-MGI:2147790</td></tr>
<tr><td>GO biological process</td><td>GO:0031528</td><td>microvillus membrane</td><td>18</td><td>0</td><td>0.01927</td><td>17</td><td>1</td><td>Itgb3</td><td>Eps8l2-MGI:1349482|Lcp1-MGI:98931|Wnk4-MGI:1349482|Wnk4-MGI:88276|Inpp5d-Itgb3|Itgb3-MGI:1342284|Itgb3-MGI:103098|Itgb3-MGI:1349482|Itgb3-MGI:96608|Itgb3-MGI:1928901|Itgb3-MGI:88323|Itgb3-MGI:106926|Itgb3-MGI:893578|Itgb3-MGI:892977|Itgb3-MGI:1351317|Itgb3-MGI:88276|Itgb3-MGI:98931</td></tr>
<tr><td>GO biological process</td><td>GO:0022612</td><td>gland morphogenesis</td><td>29</td><td>0</td><td>0.019</td><td>27</td><td>0</td><td></td><td>Sstr2-MGI:95294|Col2a1-MGI:88180|Col2a1-MGI:103302|Aldh1a3-MGI:97490|Aldh1a3-MGI:98371|Eps8l2-MGI:1349482|Lcp1-MGI:97167|Ptn-MGI:98958|Ptn-MGI:98371|Wnk4-MGI:105100|Wnk4-MGI:1349482|Wnk4-MGI:95522|Wnk4-MGI:97364|Inpp5d-MGI:105100|Inpp5d-MGI:98729|Inpp5d-MGI:103034|Inpp5d-MGI:95522|Inpp5d-MGI:97364|Itgb3-MGI:105100|Itgb3-MGI:97167|Itgb3-MGI:98729|Itgb3-MGI:88180|Itgb3-MGI:1349482|Itgb3-MGI:98371|Itgb3-MGI:95522|Itgb3-MGI:95294|Itgb3-MGI:103302</td></tr>
</table>

GO biological process	GO:0098636	protein complex involved in cell adhesion	28	0	0.01895	26	1	Itgb3	Ano1-MGI:96600|Lcp1-MGI:96892|Inpp5d-MGI:96892|Inpp5d-MGI:96616|Inpp5d-Itgb3|Itgb3-MGI:96605|Itgb3-MGI:96604|Itgb3-MGI:96603|Itgb3-MGI:96599|Itgb3-MGI:96602|Itgb3-MGI:96608|Itgb3-MGI:96606|Itgb3-MGI:1338035|Itgb3-MGI:96600|Itgb3-MGI:96892|Itgb3-MGI:96614|Itgb3-MGI:96613|Itgb3-MGI:96616|Itgb3-MGI:96615|Itgb3-MGI:97631|Itgb3-MGI:104756|Itgb3-MGI:1350360|Itgb3-MGI:96610|Itgb3-MGI:96611|Itgb3-MGI:2442114|Itgb3-MGI:102700
GO biological process	GO:0005520	insulin-like growth factor binding	14	0	0.01895	13	2	Igfbp6|Itgb3	Igf2-MGI:96437|Wnk4-MGI:96575|Igfbp6-MGI:96440|Igfbp6-MGI:96438|Inpp5d-MGI:96575|Inpp5d-Itgb3|Itgb3-MGI:96605|Itgb3-MGI:96433|Itgb3-MGI:96608|Itgb3-MGI:96613|Itgb3-MGI:96575|Itgb3-MGI:96438|Itgb3-MGI:96437
GO biological process	GO:0070293	renal absorption	14	0	0.01895	13	1	Wnk4	Scin-MGI:95851|Eps8l2-MGI:1349482|Lcp1-MGI:95851|Ptn-MGI:95851|Wnk4-MGI:107387|Wnk4-MGI:1349482|Wnk4-MGI:1333777|Wnk4-MGI:2445185|Wnk4-MGI:1314647|Wnk4-MGI:1927248|Wnk4-MGI:103201|Itgb3-MGI:1349482|Itgb3-MGI:103201
GO biological process	GO:0090193	positive regulation of glomerulus development	13	0	0.01883	12	1	Itgb3	Cryab-MGI:95295|Sstr2-MGI:87966|Col2a1-MGI:104327|Inpp5d-MGI:105304|Inpp5d-Itgb3|Itgb3-MGI:1097680|Itgb3-MGI:97902|Itgb3-MGI:106675|Itgb3-MGI:97528|Itgb3-MGI:95295|Itgb3-MGI:104327|Itgb3-MGI:87966
GO biological process	GO:0071679	commissural neuron axon guidance	13	0	0.01883	12	0		Cryab-MGI:103178|Scin-MGI:101864|Dcn-MGI:106206|Sema3c-MGI:106206|Wnk4-MGI:97281|Wnk4-MGI:99611|Inpp5d-MGI:97281|Inpp5d-MGI:99611|Itgb3-MGI:108476|Itgb3-MGI:103178|Itgb3-MGI:101864|Itgb3-MGI:105373
GO biological process	GO:0002162	dystroglycan binding	11	0	0.01855	10	0		Cryab-MGI:94909|Cryab-MGI:97175|Scin-MGI:101864|Lcp1-MGI:1923749|Lcp1-MGI:98927|Ptn-MGI:87961|Wnk4-MGI:94909|Wnk4-MGI:87961|Itgb3-MGI:94909|Itgb3-MGI:101864
GO biological process	GO:0033632	regulation of cell-cell adhesion mediated by integrin	11	0	0.01855	10	1	Piezo1	Scin-MGI:1298390|Wnk4-MGI:2442092|Inpp5d-MGI:1925723|Inpp5d-MGI:88332|Inpp5d-MGI:2147790|Itgb3-MGI:1298390|Itgb3-MGI:1925723|Itgb3-MGI:88323|Itgb3-MGI:88332|Itgb3-MGI:2147790
GO biological process	GO:0030835	negative regulation of actin filament depolymerization	21	0	0.01846	19	1	Scin	Cryab-MGI:101757|Cryab-MGI:87918|Cryab-MGI:104652|Scin-MGI:95851|Scin-MGI:97887|Scin-MGI:1346078|Scin-MGI:87918|Scin-MGI:87919|Scin-MGI:98809|Scin-MGI:1298390|Scin-MGI:107717|Scin-MGI:104684|Scin-MGI:700006|Scin-MGI:104652|Eps8l2-MGI:104684|Lcp1-MGI:95851|Lcp1-MGI:87918|Ptn-MGI:95851|Itgb3-MGI:1298390
GO biological process	GO:0033628	regulation of cell adhesion mediated by integrin	40	0	0.01836	36	2	Piezo1|Itgb3	Scin-MGI:1298390|Aldh1a3-MGI:88590|Lcp1-MGI:95481|Lcp1-MGI:2443583|Lcp1-MGI:96892|Lcp1-MGI:88508|Wnk4-MGI:95481|Wnk4-MGI:2442092|Inpp5d-MGI:1925723|Inpp5d-MGI:99515|Inpp5d-MGI:1926063|Inpp5d-MGI:104686|Inpp5d-MGI:99511|Inpp5d-MGI:96892|Inpp5d-MGI:88332|Inpp5d-MGI:2147790|Inpp5d-MGI:88508|Inpp5d-MGI:96055|Inpp5d-MGI:95278|Inpp5d-MGI:2147677|Inpp5d-Itgb3|Itgb3-MGI:1925723|Itgb3-MGI:88323|Itgb3-MGI:1298390|Itgb3-MGI:99515|Itgb3-MGI:2443583|Itgb3-MGI:97608|Itgb3-MGI:105376|Itgb3-MGI:96892|Itgb3-MGI:88332|Itgb3-MGI:2147790|Itgb3-MGI:96055|Itgb3-MGI:97902|Itgb3-MGI:1344418|Itgb3-MGI:1918089|Itgb3-MGI:95278
GO biological process	GO:0033630	positive regulation of cell adhesion mediated by integrin	19	0	0.01825	17	2	Piezo1|Itgb3	Lcp1-MGI:2443583|Inpp5d-MGI:1925723|Inpp5d-MGI:88332|Inpp5d-MGI:99515|Inpp5d-MGI:96055|Inpp5d-MGI:1926063|Inpp5d-Itgb3|Itgb3-MGI:1925723|Itgb3-MGI:88323|Itgb3-MGI:88332|Itgb3-MGI:2443583|Itgb3-MGI:99515|Itgb3-MGI:96055|Itgb3-MGI:97902|Itgb3-MGI:1344418|Itgb3-MGI:1918089|Itgb3-MGI:105376

<table>
<tr><th></th><th></th><th></th><th></th><th></th><th></th><th></th><th></th><th></th><th></th></tr>
<tr><td>GO biological process</td><td>GO:2001028</td><td>positive regulation of endothelial cell chemotaxis</td><td>16</td><td>0</td><td>0.01785</td><td>14</td><td>0</td><td></td><td>Cryab-MGI:103178|Cryab-MGI:96240|Aldh1a3-MGI:95516|Dcn-MGI:99879|Dcn-MGI:96240|Dcn-MGI:96683|Sema3c-MGI:96969|Wnk4-MGI:95522|Inpp5d-MGI:95522|Itgb3-MGI:97528|Itgb3-MGI:95516|Itgb3-MGI:99879|Itgb3-MGI:103178|Itgb3-MGI:95522</td></tr>
<tr><td>GO biological process</td><td>GO:0030643</td><td>cellular phosphate ion homeostasis</td><td>7</td><td>0</td><td>0.01749</td><td>6</td><td>0</td><td></td><td>Eps8l2-MGI:1349482|Wnk4-MGI:1891427|Wnk4-MGI:1349482|Wnk4-MGI:97370|Itgb3-MGI:1342284|Itgb3-MGI:1349482</td></tr>
<tr><td>GO biological process</td><td>GO:0016494</td><td>C-X-C chemokine receptor activity</td><td>7</td><td>0</td><td>0.01749</td><td>6</td><td>0</td><td></td><td>Ano1-MGI:109563|Itgb3-MGI:109562|Itgb3-MGI:109563|Itgb3-MGI:1277207|Itgb3-MGI:1934582|Itgb3-MGI:105303</td></tr>
<tr><td>GO biological process</td><td>GO:0034446</td><td>substrate adhesion-dependent cell spreading</td><td>41</td><td>0</td><td>0.01742</td><td>35</td><td>1</td><td>Itgb3</td><td>Cryab-MGI:892014|Cryab-MGI:98397|Dcn-MGI:106206|Dcn-MGI:98397|Sema3c-MGI:106206|Sema3c-MGI:95566|Lcp1-MGI:108295|Lcp1-MGI:98397|Lcp1-MGI:2385001|Lcp1-MGI:87859|Ptn-MGI:98397|Wnk4-MGI:96965|Inpp5d-MGI:96616|Inpp5d-MGI:96965|Inpp5d-MGI:2147790|Inpp5d-MGI:98397|Inpp5d-MGI:2385001|Inpp5d-MGI:107381|Inpp5d-MGI:95566|Inpp5d-MGI:87859|Inpp5d-Itgb3|Inpp5d-MGI:98664|Itgb3-MGI:96603|Itgb3-MGI:96608|Itgb3-MGI:108295|Itgb3-MGI:1916788|Itgb3-MGI:96616|Itgb3-MGI:97845|Itgb3-MGI:2147790|Itgb3-MGI:1195267|Itgb3-MGI:98397|Itgb3-MGI:95566|Itgb3-MGI:87859|Itgb3-MGI:109442|Itgb3-MGI:98664</td></tr>
<tr><td>GO biological process</td><td>GO:0072678</td><td>T cell migration</td><td>19</td><td>0</td><td>0.01718</td><td>16</td><td>0</td><td></td><td>Ano1-MGI:101924|Lcp1-MGI:97167|Lcp1-MGI:88508|Inpp5d-MGI:96616|Inpp5d-MGI:88508|Inpp5d-MGI:104686|Itgb3-MGI:1333797|Itgb3-MGI:96603|Itgb3-MGI:96392|Itgb3-MGI:96616|Itgb3-MGI:97167|Itgb3-MGI:1352450|Itgb3-MGI:1277207|Itgb3-MGI:101924|Itgb3-MGI:1096355|Itgb3-MGI:106185</td></tr>
<tr><td>GO biological process</td><td>GO:0005581</td><td>collagen trimer</td><td>25</td><td>0</td><td>0.01714</td><td>21</td><td>6</td><td>Col9a3|Col2a1|Col9a2|Col9a1|Col10a1|Dcn</td><td>Loxl4-MGI:96817|Col2a1-MGI:1858212|Col2a1-MGI:88457|Col2a1-MGI:104688|Col2a1-MGI:88458|Col2a1-MGI:88453|Col2a1-MGI:88468|Col2a1-MGI:88467|Col2a1-MGI:88446|Col2a1-MGI:2672118|Dcn-MGI:1858212|Dcn-MGI:88457|Dcn-MGI:104688|Dcn-MGI:88458|Dcn-MGI:88453|Dcn-MGI:109347|Dcn-MGI:88468|Dcn-MGI:88467|Dcn-MGI:88446|Dcn-MGI:2672118|Loxl2-MGI:96817</td></tr>
<tr><td>GO biological process</td><td>GO:0050966</td><td>detection of mechanical stimulus involved in sensory perception of pain</td><td>23</td><td>0</td><td>0.01685</td><td>19</td><td>1</td><td>Ano1</td><td>Ano1-MGI:95820|Ano1-MGI:109521|Ano1-MGI:3522699|Ano1-MGI:96824|Ano1-MGI:96654|Ano1-MGI:109563|Ano1-MGI:95821|Ano1-MGI:96600|Igf2-MGI:104798|Lcp1-MGI:95602|Ptn-MGI:95602|Wnk4-MGI:96654|Inpp5d-MGI:96824|Inpp5d-MGI:95602|Itgb3-MGI:96824|Itgb3-MGI:95602|Itgb3-MGI:103556|Itgb3-MGI:109563|Itgb3-MGI:96600</td></tr>
<tr><td>GO biological process</td><td>GO:0030834</td><td>regulation of actin filament depolymerization</td><td>34</td><td>0</td><td>0.0168</td><td>28</td><td>1</td><td>Scin</td><td>Cryab-MGI:101757|Cryab-MGI:87918|Cryab-MGI:109192|Cryab-MGI:104652|Scin-MGI:97887|Scin-MGI:87918|Scin-MGI:87919|Scin-MGI:98809|Scin-MGI:1298390|Scin-MGI:1915982|Scin-MGI:104684|Scin-MGI:1929270|Scin-MGI:95851|Scin-MGI:1346078|Scin-MGI:1923959|Scin-MGI:107717|Scin-MGI:700006|Scin-MGI:104652|Eps8l2-MGI:104684|Lcp1-MGI:87918|Lcp1-MGI:95851|Lcp1-MGI:1923959|Lcp1-MGI:109192|Ptn-MGI:95851|Inpp5d-MGI:1860485|Itgb3-MGI:1860485|Itgb3-MGI:1298390|Itgb3-MGI:1923959</td></tr>
<tr><td>GO biological process</td><td>GO:0032463</td><td>negative regulation of protein homooligomerization</td><td>11</td><td>0</td><td>0.01669</td><td>9</td><td>1</td><td>Cryab</td><td>Cryab-MGI:1890467|Cryab-MGI:98397|Dcn-MGI:98397|Lcp1-MGI:98397|Ptn-Cryab|Ptn-MGI:98397|Wnk4-MGI:1859285|Inpp5d-MGI:98397|Itgb3-MGI:98397</td></tr>
</table>

<table>
<tr><td>GO biological process</td><td>GO:0010543</td><td>regulation of platelet activation</td><td>33</td><td>0</td><td>0.01669</td><td>27</td><td>0</td><td></td><td>Apod-MGI:88057|Ano1-MGI:96824|Sema3c-MGI:97527|Lcp1-MGI:96892|Ptn-MGI:97527|Wnk4-MGI:108174|Inpp5d-MGI:1860485|Inpp5d-MGI:97601|Inpp5d-MGI:96824|Inpp5d-MGI:99515|Inpp5d-MGI:1347245|Inpp5d-MGI:95496|Inpp5d-MGI:96892|Itgb3-MGI:98280|Itgb3-MGI:1860485|Itgb3-MGI:88057|Itgb3-MGI:99670|Itgb3-MGI:88348|Itgb3-MGI:96824|Itgb3-MGI:97595|Itgb3-MGI:99515|Itgb3-MGI:1347245|Itgb3-MGI:103098|Itgb3-MGI:95496|Itgb3-MGI:96892|Itgb3-MGI:97528|Itgb3-MGI:97795</td></tr>
<tr><td>GO biological process</td><td>GO:0001954</td><td>positive regulation of cell-matrix adhesion</td><td>52</td><td>0</td><td>0.01648</td><td>42</td><td>1</td><td>Itgb3</td><td>Cryab-MGI:94909|Cryab-MGI:103178|Cryab-MGI:101757|Apod-MGI:97515|Scin-MGI:101815|Scin-MGI:101864|Dcn-MGI:106206|Dcn-MGI:96683|Sema3c-MGI:106206|Lcp1-MGI:97602|Lcp1-MGI:2443583|Lcp1-MGI:96650|Lcp1-MGI:87859|Wnk4-MGI:97602|Wnk4-MGI:94909|Inpp5d-MGI:1277162|Inpp5d-MGI:107381|Inpp5d-MGI:98664|Inpp5d-MGI:1925723|Inpp5d-MGI:98747|Inpp5d-MGI:88332|Inpp5d-MGI:1339753|Inpp5d-MGI:1928144|Inpp5d-MGI:87859|Inpp5d-Itgb3|Itgb3-MGI:99551|Itgb3-MGI:2443583|Itgb3-MGI:94909|Itgb3-MGI:103178|Itgb3-MGI:1344418|Itgb3-MGI:1201674|Itgb3-MGI:98664|Itgb3-MGI:1925723|Itgb3-MGI:104908|Itgb3-MGI:1352757|Itgb3-MGI:104631|Itgb3-MGI:88332|Itgb3-MGI:97845|Itgb3-MGI:1195267|Itgb3-MGI:1339753|Itgb3-MGI:101864|Itgb3-MGI:87859</td></tr>
<tr><td>GO biological process</td><td>GO:1900026</td><td>positive regulation of substrate adhesion-dependent cell spreading</td><td>37</td><td>0</td><td>0.01599</td><td>29</td><td>1</td><td>Itgb3</td><td>Scin-MGI:1931838|Scin-MGI:1915982|Scin-MGI:1923959|Dcn-MGI:106206|Sema3c-MGI:106206|Lcp1-MGI:1931838|Lcp1-MGI:88508|Lcp1-MGI:1923959|Lcp1-MGI:87859|Ptn-MGI:1931838|Wnk4-MGI:95556|Wnk4-MGI:109547|Inpp5d-MGI:104686|Inpp5d-MGI:3040696|Inpp5d-MGI:88508|Inpp5d-MGI:87859|Inpp5d-Itgb3|Inpp5d-MGI:109547|Itgb3-MGI:1931838|Itgb3-MGI:99670|Itgb3-MGI:107821|Itgb3-MGI:95556|Itgb3-MGI:97845|Itgb3-MGI:3040696|Itgb3-MGI:97302|Itgb3-MGI:1923959|Itgb3-MGI:1344418|Itgb3-MGI:87859|Itgb3-MGI:109547</td></tr>
<tr><td>GO biological process</td><td>GO:0090190</td><td>positive regulation of branching involved in ureteric bud morphogenesis</td><td>23</td><td>0</td><td>0.01597</td><td>18</td><td>0</td><td></td><td>Cryab-MGI:103178|Apod-MGI:1344337|Sstr2-MGI:87966|Col2a1-MGI:104327|Col2a1-MGI:88180|Aldh1a3-MGI:102780|Aldh1a3-MGI:106034|Aldh1a3-MGI:98371|Ptn-MGI:98371|Wnk4-MGI:1891468|Itgb3-MGI:103178|Itgb3-MGI:98371|Itgb3-MGI:1344337|Itgb3-MGI:98725|Itgb3-MGI:104327|Itgb3-MGI:1891468|Itgb3-MGI:88180|Itgb3-MGI:87966</td></tr>
<tr><td>GO biological process</td><td>GO:0042482</td><td>positive regulation of odontogenesis</td><td>13</td><td>0</td><td>0.01569</td><td>10</td><td>0</td><td></td><td>Sstr2-MGI:105923|Col2a1-MGI:1333772|Aldh1a3-MGI:104671|Dcn-MGI:97323|Igf2-MGI:88177|Inpp5d-MGI:1339753|Itgb3-MGI:88177|Itgb3-MGI:1339753|Itgb3-MGI:95283|Itgb3-MGI:98725</td></tr>
<tr><td>GO biological process</td><td>GO:0030320</td><td>cellular monovalent inorganic anion homeostasis</td><td>16</td><td>0</td><td>0.0153</td><td>12</td><td>1</td><td>Wnk4</td><td>Ano1-MGI:1862037|Eps8l2-MGI:1349482|Lcp1-MGI:95481|Wnk4-MGI:1352447|Wnk4-MGI:1349482|Wnk4-MGI:109482|Wnk4-MGI:95481|Wnk4-MGI:1862037|Wnk4-MGI:1891427|Wnk4-MGI:97370|Itgb3-MGI:1342284|Itgb3-MGI:1349482</td></tr>
<tr><td>GO biological process</td><td>GO:1990869</td><td>cellular response to chemokine</td><td>16</td><td>0</td><td>0.0153</td><td>12</td><td>0</td><td></td><td>Loxl4-MGI:96817|Ano1-MGI:101924|Loxl2-MGI:96817|Lcp1-MGI:1096342|Wnk4-MGI:1858416|Wnk4-MGI:2442092|Inpp5d-MGI:87940|Itgb3-MGI:1921396|Itgb3-MGI:1858416|Itgb3-MGI:101924|Itgb3-MGI:103556|Itgb3-MGI:1096342</td></tr>
<tr><td>GO biological process</td><td>GO:0033627</td><td>cell adhesion mediated by integrin</td><td>19</td><td>0</td><td>0.01503</td><td>14</td><td>1</td><td>Itgb3</td><td>Ano1-MGI:96600|Dcn-MGI:95489|Inpp5d-Itgb3|Itgb3-MGI:96605|Itgb3-MGI:96604|Itgb3-MGI:96603|Itgb3-MGI:96392|Itgb3-MGI:96608|Itgb3-MGI:96615|Itgb3-MGI:96610|Itgb3-MGI:2442114|Itgb3-MGI:96600|Itgb3-MGI:1096335|Itgb3-MGI:105376</td></tr>
</table>
